# A systematic review of interventions that impact alcohol and other drug-related harms in licensed entertainment settings and outdoor music festivals

**DOI:** 10.1186/s12954-024-00949-4

**Published:** 2024-02-21

**Authors:** Christopher Eassey, Caitlin E. Hughes, Phillip Wadds, Dominique de Andrade, Monica J. Barratt

**Affiliations:** 1 National Drug and Alcohol Research Centre, UNSW Sydney, Sydney, Australia; 2 Centre for Crime Policy and Research, Flinders University, Adelaide, Australia; 3 The School of Law, Society and Criminology, and Centre for Criminology, Law and Justice, Faculty of Law and Justice, UNSW Sydney, Sydney, Australia; 4 Griffith Criminology Institute, Griffith University, Brisbane, Australia; 5 School of Psychology, University of Queensland, Brisbane, Australia; 6 Centre for Drug Use, Addictive and Anti-Social Behaviour Research, School of Psychology, Deakin University, Geelong, Australia; 7 Social Equity Research Centre and Digital Ethnography Research Centre, RMIT University, 124 La Trobe Street, Melbourne, VIC 3000 Australia

**Keywords:** Harm reduction, Alcohol, Illicit drugs, Licensed entertainment settings, Music festivals/festivals, Systematic review

## Abstract

**Background:**

Harms associated with the use of alcohol and other drugs (AOD) in licensed entertainment settings (LES) and outdoor music festivals (OMF) are ongoing public health and criminal justice concerns. This systematic review provides a comprehensive, synthesized report on the evidence base of interventions that impact harm in these settings, and how they affect health, behavioral, and criminal justice outcomes.

**Methods:**

Nine databases were searched for experimental and observational studies published between 2010 and 2021. Studies were included if they were peer-reviewed, published in English, described interventions which could impact AOD-related harms in LES or OMF (and were delivered in these environments), and reported on health, criminal justice and/or behavioral outcomes. Methodological quality was assessed using the Effective Public Health Practice Project’s Quality Assessment Tool for Quantitative Studies and the Critical Appraisal Skills Program for qualitative studies. A narrative synthesis was conducted to synthesize outcomes across studies. The review protocol was registered in PROSPERO (CRD42020140004).

**Results:**

Of the 48,303 studies screened, 100 met the inclusion criteria. 86 focused solely on reducing alcohol-related harm, 7 on reducing illicit drug-related harm, and 7 on both. Most (*n* = 88) focused on LES and evaluated changes in laws and regulations (*n* = 28) and/or multicomponent interventions/policies (*n* = 41). Multicomponent interventions showed the best results for both health (62% positive) and criminal justice (84% positive) outcomes, with 71% of studies being rated as strong quality. There was also good evidence to support the careful application of trading hour restrictions and limited but promising evidence to support medical services and drug checking.

**Conclusion:**

The breadth, quality and volume of evidence regarding what works in reducing AOD-related harm in recreational settings have increased in the past decade, particularly regarding LES. Findings support onsite medical services (reducing ambulance transfer rates), multicomponent interventions targeting alcohol accessibility and availability (reducing assaults), and drug checking services, but suggest other interventions such as drug detection dogs may exacerbate harm. Further, higher quality research is required to address identified gaps in the evidence base, particularly on optimal interventions within OMF, around illicit drugs more broadly and in the Global South.

**Supplementary Information:**

The online version contains supplementary material available at 10.1186/s12954-024-00949-4.

## Introduction

Alcohol and other drug (AOD) use is common in licensed entertainment settings (LES) and outdoor music festivals (OMF) [1]. Findings from self-report and oral swab studies in Australia, Europe and the USA suggest that as much as 48% of patrons in LES and OMF have used illicit drugs on any given night [2–7]. Drugs commonly used in LES and OMF settings include a broad range of stimulants (e.g., cocaine, amphetamines), empathogens (e.g., MDMA), dissociatives (e.g., ketamine), psychedelics (e.g., LSD, psilocybin) and sedatives (e.g., cannabis and benzodiazepines) [8–10]. Risky alcohol use is also common among young adults in these settings [11–14]**.**

AOD use in LES and OMF has been associated with a diverse array of harms. These harms include excessive intoxication and overdose [15], poor mental health [16, 17], offending behaviors such as violence or public disorder offenses [18, 19], sexual risk-taking [1], alcohol- and other drug-affected driving [12, 20], sexual violence [21, 22], risk of victimization [23], and death [24]. As such, AOD use in these settings can place a significant burden on acute health services including ambulances and hospitals [25, 26]. To prevent and/or reduce the prevalence and impact of these harms, there have been growing number of services, programs, campaigns and interventions introduced around the world.

### Harm reduction in licensed entertainment settings and festivals

Harm reduction refers to reducing the health, criminal justice or social harms associated with AOD use without necessarily eliminating use [27]. A number of harm reduction interventions have been trialled and implemented in LES and OMF specifically, including drug checking [28], transport interventions [29, 30], onsite medical services [31], chill out/roaming support services [32], liquor licensing laws and regulations [33, 34], licensed venue staff training [35, 36], and policing and compliance strategies [37]. These interventions vary in their focus regarding the targeted population group, settings, targeted harm(s) and drug type. In addition, changes in drug-related harms may be an unintended consequence for some strategies that primarily target crime and antisocial behavior (e.g., police using drug detection dogs). In recent years, many of these individual measures have been combined to develop more complex, population-wide, multicomponent approaches that encourage change in local policies, structures, systems, and AOD use cultures. A detailed explanation of each intervention described in this study is provided in Table 1.

**Table 1:** Intervention types definitions

*Laws and regulations*	*Lockout*
	A “one-way door policy”, whereby entry or re-entry to a licensed venue is prohibited after a specified time, but patrons remaining inside the venue past this time can continue purchasing and consuming until the venue closes
	*Risk-based licensing (RBL)*
	Policies applied specifically to premises assessed as being of high risk of alcohol-related harms that may include tailored measures and/or liquor license fees that are levied according to assessed venue risks
	*Alcohol availability restrictions*
	These policies aim to restrict the availability of alcohol to consumers and include the banning the sale of "rapid intoxication liquor" (e.g., shots) after midnight or prohibiting the sale of spirits, aiming to mitigate alcohol-related harms by controlling the types and timing of alcohol consumption
	*Trade hour restrictions and extensions*
	Restrictions or extensions to the permissible hours during which alcohol can be sold or consumed, strategically impacting alcohol-related harms by controlling availability and consumption patterns to promote safer and more controlled recreational environments
	*Staggered closing times*
	Varied end-of-service hours for various licensed premises aiming to mitigate alcohol-related harms by reducing the simultaneous dispersal of alcohol-affected patrons onto the streets
	*Legal purchase age*
	The legally established minimum age at which individuals are permitted to buy alcohol, that aims to mitigate potential harm by restricting access and consumption among underage patrons. Minimum ages vary across the world
	*Smoking bans*
	Regulatory prohibitions on the consumption of tobacco (and sometimes nicotine through electronic nicotine devices). These bans are interrelated with alcohol and other drug-related harm mitigation by reducing exposure to second-hand smoke, risk of drink spiking when patrons left drinks unattended to smoke outside, and risk of overcrowding in smoking areas
	*Regulatory compliance and enforcement*
	The rigorous oversight and implementation of responsible service of alcohol (RSA) regulations by law enforcement, liquor licensing authorities, and venue personnel, that aims to mitigate AOD-related harms
*Policing strategies*	Strategies involving police, such as the use of drug detection dogs, implementation of patron bans, and the application of varying levels of policing enforcement responses
*Medical services*	Services such as onsite medical personnel, first aid resources, and rapid first response services. These services aim to contribute to enhanced care for attendees and mitigate the strain on local resources by reducing hospitalizations and ambulance transfers
*Chill/safe space and roaming support services*	Multi-faceted services that typically encompass drug education booths, peer led AOD care initiatives (e.g., quiet “chill” spaces for those recovering from the effects of substances), and the distribution of harm reduction resources like sunscreen, condoms, water, and earplugs. These initiatives align with onsite medical services by proactively offering assistance, promoting informed decision-making, and cultivating a safer, more supportive event environment
*Transport interventions*	These interventions encompass 24-h public transportation, designated driver initiatives, ride-share services, and accessible taxi stands, and are offered as strategic measures to curtail alcohol and drug-related harms by mitigating alcohol- and drug-impaired driving, ensuring the safe transportation of affected individuals, and concurrently diminishing incidents of aggression and assault within licensed entertainment areas
*Staff and venue intervention*	These interventions include staff RSA training, the voluntary use of hand-held breathalyzers to discourage excessive intoxication, in-bar media campaigns promoting water hydration, violence de-escalation training for bar staff, and the strategic deployment of capable guardians such as bouncers, door attendants, and barricades. Staff and venue interventions create safer environments and curb substance-related issues
*Drug checking/pill testing*	This intervention aims to analyze drug samples directly from the service user, return results to the service user, engage in information exchange between the service user and the service regarding the user and the content and strength/dose of the drug, and provide a tailored intervention
*Patron survey and assessment feedback*	This intervention includes a street intercept survey with personalized normative feedback on alcohol and other drug-related risks with tips to reduce related harm
*Multicomponent interventions*	*Sydney “lockout laws”*
	The “Lockout Laws” in Sydney, Australia, were a set of policy interventions packaged under the official title of the Plan of Management for the Sydney CBD Entertainment Precinct which included: (i) a 1:30am lockout at pubs, bars, registered clubs, nightclubs and karaoke bars; (ii) a 3am cessation of alcohol service in all venues; (iii) a prohibition on the granting of any new liquor licenses; (iv) a ban on takeaway alcohol sales across NSW after 10pm; (v) an extension of ‘banning orders’ on designated ‘trouble-makers’ to prevent them entering most licensed premises in the Kings Cross and Sydney’s CBD precincts; (vi) a ban on ‘shots’ and any ready-to-drink beverage with an alcohol by volume content of more than 5%; and (vii) the introduction of a risk-based license fee for all licensed premises based on license type, compliance history and trading hours
	*Queensland “tackling alcohol-fuelled violence” (TAFV) policy*
	The TAFV policy in Queensland, Australia, included the following in stages: (i) a restriction on the service of alcohol from 5 to 3am in the state’s 15 designated late-night entertainment precincts (Safe Night Precincts; SNPs); (ii) a ban on the sale of shots (or high-alcohol beverages); and (iii) mandatory ID scanners
	*Newcastle liquor licensing restrictions*
	The Newcastle Central Business District (CBD) multicomponent intervention included: (i) earlier closing (3am) and lockout (1am), relaxed to 3:30am and 1:30am months later, (ii) the requirement for licensees to adopt management plans, which were subject to compliance audits, and had a dedicated RSA officer from 11pm until closing, (iii) a ban on serving shots after 10pm, and cessation of selling alcohol 30 min before closing, (iv) a ban on drink stockpiling, and (v) requirements to adopt shared radio procedures and notify all staff of the conditions
	*US multicomponent interventions*
	Multicomponent interventions trialled in the USA have largely been designed with RSA and law/liquor licensing enforcement measures at their core
	*New Zealand’s sale and supply of alcohol act*
	The Sale and Supply of Alcohol Act included trading hour limits for on-licensed and off-licensed premises, new RBL fees, changes to licensing procedures, and the legal enforcement of one-way door policies
	*UK multicomponent studies*
	These largely include Cumulative Impact Zones (CIZ)—specific zones within night-time entertainment districts whereby local authorities may apply additional measures to reduce harm and increase public safety
	*SALUTT and STAD interventions in Northern Europe*
	The STAD in Sweden focused on RSA training, stricter enforcement of existing alcohol laws by bar staff and police, and the development of community coalition steering groups. The SALUTT intervention in Norway was modelled on STAD, with noted differences including an increased emphasis placed on dialog relative to sanctions, and a less central role played by the police regarding licensing and control

Given these types of interventions are increasingly implemented across the world, it is crucial for policymakers and service providers to understand the extent to which they are effective in achieving their intended outcomes, the mechanisms that drive outcomes, whether their effectiveness is limited to particular populations or settings, and/or any unintended consequences. There remain several gaps in knowledge that have not been addressed by existing reviews in the field [15, 33–38], as summarized in Additional file 1. First, despite the expansion of interventions that impact AOD-related harms, existing reviews are either outdated or limited in their focus to either LES or OMF settings. Second, they do not consider the full array of levers that could influence or affect harm reduction (i.e., laws, policies, services, training, education, policing, governance mechanisms). Third, most do not provide a methodological quality assessment of studies. Fourth, most existing reviews focus on alcohol or drug-related harm, but not both (despite the frequent simultaneous use of these substances in such settings). Finally, there has been significant expansion of interventions aimed at reducing harm in LES and OMF settings in recent years—the evidence from which is yet to be comprehensively synthesized or reviewed.

This review aims to address these critical knowledge gaps, providing an updated synthesis of interventions that impact harms in LES and OMF settings. We chose the term 'impact' rather than 'effectiveness' to encompass the various outcomes of these interventions—negative, null, or positive—and the diverse effects across domains like health, criminal justice, and behavioral outcomes. This choice allows us to evaluate not just how interventions reduce harm, but also their broader impact, including instances where they may increase harm. Further, the review utilizes appropriate tools to assess the quality of the existing evidence across a broad range of health, criminal justice, and behavioral outcomes. To the best of our knowledge, this review has the largest scope to date regarding intervention types and outcomes examined and, as such, provides evidence to inform AOD harm reduction policy and practice for LES and OMF settings.

## Method

### Reporting and protocol

The reporting for this systematic review was guided by the Preferred Reporting Items for Systematic Reviews and Meta-Analyses (PRISMA) [39] (also see Additional file 2, PRISM Checklist) and the guidelines provided in the Cochrane Consumers and Communication Review Group: Data synthesis and analysis [40]. The review protocol was registered in PROSPERO (CRD42020140004). The categories of outcome variables were largely predetermined and listed in the protocol based on prior reviews (e.g., 15, 35) and authors’ expert knowledge of the field. They were adjusted following the search strategy and data extraction completion to accommodate any unexpected/overlooked outcomes.

### Search strategy

The search strategy and syntax were developed, piloted, and refined in consultation with a library expert from the University of New South Wales. Medical subject headings (MeSH) and keywords were utilized and modified for each database as relevant to LES and OMF harm reduction services. These keywords were applied using PICO for quantitative studies—Population or problem, Intervention or exposure, Comparison, and Outcome [41]—and PICo for qualitative studies—Population or Problem, Interest, and Context [42]. The first search was conducted in January 2021 and covered articles from 2010 to 2020 inclusive. An updated search was conducted on January 10, 2022, to include all articles published in the year 2021. To ensure the review was comprehensive and captured pertinent studies across health, criminal justice and social science disciplines, we searched nine commonly used databases, including: MEDLINE, Embase, Cochrane Central Register of Controlled Trials, CINAHL, ProQuest Social Science database, Criminal Justice Abstracts, Web of Science, PsycINFO, and PubMed. The search was limited to title and abstract. An overview of the search strategy is provided in Table 2 (formatted for PubMed). The full search strategies for each database are listed in Additional file 3. Database search results were imported into the Covidence systematic review software for duplicate removal and screening.

**Table 2:** Search string (PubMed)

1. Drug use OR substance use OR illicit drug* OR illegal drug* OR narcotic* OR recreational drug* OR street drug* OR alcohol OR polydrug [Title/Abstract]
2. Designer drug* OR club drug* OR legal high* OR stimulant* OR hallucinogen*
3. Alcohol OR beverage* OR liquor* OR binge* OR drunk [Title/Abstract]
4. Ecstasy OR MDMA OR mushrooms OR psilocybin OR LSD OR amphetamines OR dexamphetamine OR methamphetamine OR cocaine OR cannabis OR mephedrone OR GHB OR ketamine OR poppers OR amyl nitrite OR phenethylamines OR DMT [Title/Abstract]
5. 1 OR 2 OR 3 OR 4
6. Substance Abuse Detection [MeSH]
7. Alcohol drinking/prevention and control [MeSH]
8. Substance-related disorders/prevention and control [MeSH]
9. Health promotion/methods [MeSH]
10. Risk Management [MeSH]
11. Laws OR policy OR policing OR governance OR intervention OR licens* OR education OR service* OR community OR framework
12. Medical intervention OR evidence based OR monitor OR risk reduction OR drug safety testing OR drug checking OR pill testing OR urban design OR environment* OR medical first aid OR multicomponent OR legislat* OR decriminalisation OR deterrence OR staff training OR treatment OR prevention OR evaluation OR outcome OR harm reduction OR drug policy OR alcohol policy OR health policy OR treatment OR prevention OR responsible service of alcohol OR lockouts OR chillout* OR peer group*
13. 6 OR 7 OR 8 OR 9 OR 10 OR 11 OR 12
14. Nightlife settings OR safer nightlife OR licensed venue* OR recreational setting* or licensed premise* OR entertainment precincts OR licensure OR pub OR club OR patron OR attendee* OR nightclub* OR disco* OR bar OR lounge OR festival* OR rave OR music
15. 5 AND 13 AND 14
16. Limit 15 to: language: English; publication date: 2010–2021

### Inclusion and exclusion criteria

Studies were included if they were: peer-reviewed, published in English, described interventions that could impact AOD-related harms in LES and OMF environments (and were delivered in these environments), and reported on health, criminal justice, and/or behavioral outcomes. Health outcomes included: hospitalization, road traffic accidents, ambulance presentations and deaths. Criminal justice outcomes included: assaults, driving offenses, general crime, public order offenses and liquor offenses. Behavioral outcomes included: risky AOD consumption practices (e.g., binge drinking, consuming high doses of illicit drugs, mixing alcohol and illicit drugs), aggression (i.e., not meeting the legal threshold of assault), overcrowding, and public transport use (i.e., potential to reduce drink- or drug-affected driving). Included studies could be experimental studies (randomized control trials), or observational studies, including quasi-experimental, interrupted time series (ITS), cross-sectional, pre-post, stepped design, case control, prospective cohort, implementation studies, and qualitative studies. Studies were excluded if they focused on participant groups that were adolescent/youth focused (e.g., under legal drinking age), or intervention environments that were not specifically late-night entertainment venues (e.g., liquor stores licensed for off-premises consumption). Studies were also excluded if they were grey literature, reports, dissertations, letters to editors, conferences proceedings or abstracts, study protocols, simulations, described AOD use but not AOD-related harms, or reported planned or intended behavioral change rather than actual behavior change. Grey literature was not included due to the decreased likelihood of peer review and methodological rigor, which effects the transparency and reproducibility of findings.

Behavioral outcomes in our study refer to the shifts in individual behaviors in nightlife and festival contexts that are not covered by standard health or criminal justice measures. These outcomes are key to assessing the impact of harm reduction interventions on behaviors and practices. While behavioral outcomes may slightly differ in how they are measured across studies, they must be identified by authors as a behavior that can be changed/manipulated to potentially reduce harm. Despite most studies defining these outcomes in slightly different ways, the concept of binge drinking in particular has a commonly used definition in research. That is, the consumption of four or more standard drinks in a drinking session for women, and five or more for men [43].

### Screening and data extraction

The database searches resulted in the identification of 84,524 potentially relevant articles. Following the removal of 36,221 duplicates, 48,303 titles and abstracts were screened by the lead author (CE) using Covidence software [44]. This resulted in 442 potentially eligible articles, with a decision regarding full-text screening on 120 of these articles made in consultation with at least one other author. A total of 337 eligible articles were full text screened by the lead author (CE), with each article being fully screened by a second reviewer (either author PW, MB, or CH). Discrepancies between reviewers existed for approximately 10% of these eligible articles, with disagreements being resolved through one-on-one or team discussion. Data extraction for all articles was conducted by the lead author (CE) with duplicate extractions conducted for each article by either PW, MB or CH. Any differences in extractions were discussed during regular team meetings to resolve discrepancies and develop new exclusion criteria as needed. The following data were extracted from each study: author, year of publication, country, study setting, intervention measures, method of analysis, health, criminal justice, and behavioral outcomes (as listed above), the direction of the effect (i.e., positive, negative, null, or mixed) and size of effect (if reported).

### Methodological quality assessment tools

Included quantitative studies were assessed for risk of bias and methodological quality using the Effective Public Health Practice Project (EPHPP) quality assessment tool [45]. This tool was chosen due to its high construct validity, content validity and inter-rater reliability [45]. Further, it consists of assessment criteria (including risk of bias) that accommodates all types of quantitative studies (not just randomized controlled trials). Included qualitative and mixed methods studies were assessed using the Critical Appraisal Skills Programme (CASP) appraisal tool [46]. Using these tools, the methodological rigor of each study was assessed by the lead author (CE) and a second rater (either PW, MB, CH, or DD). Conflicts were resolved by consensus. DD triple-checked data extraction and quality assessment for 10% of studies.

Using the EPHPP tool, each quantitative study was rated as strong, moderate, or weak based on six criteria: selection bias, study design, confounders, blinding, data collection method and withdrawals. Based on the tool guidelines, an overall rating was given for each study. A ‘strong’ overall rating was applied to studies that had no weak ratings for any criterion. Those with a ‘moderate’ overall rating had one weak rating, and those with a ‘weak’ overall rating had two or more weak ratings. In the rare case that all criteria had a moderate rating, we gave an overall rating of moderate (as opposed to assigning a ‘strong’ rating as recommended by the guidelines).

The CASP tool [46] assesses ten criteria, e.g. appropriateness of research design. Each criterion was scored either “yes” = 2, “no” = 0 and “can’t tell” = 1. If ≥ 8 of the criteria on the checklist were met, the study was rated as “good” quality; if 5–7 were met, it was rated as “fair” quality; and if < 5 were met, it was rated as “poor” quality.

There was significant heterogeneity in included studies’ interventions, study design, analyses and outcomes, resulting in limited studies that estimated the same population parameter [47]. Given this, and the inclusion of both quantitative and qualitative studies in this review, we could not systematically extract included studies’ effect sizes, nor conduct a meta-analysis. Instead, a narrative synthesis was employed to systematically identify common themes and findings (see, e.g., 48, 49). Herein, this review’s results are structured around intervention type, as suggested in the guidelines provided by the Cochrane Consumers and Communication Review Group [40].

## Results

### Search results

**Fig. 1:**
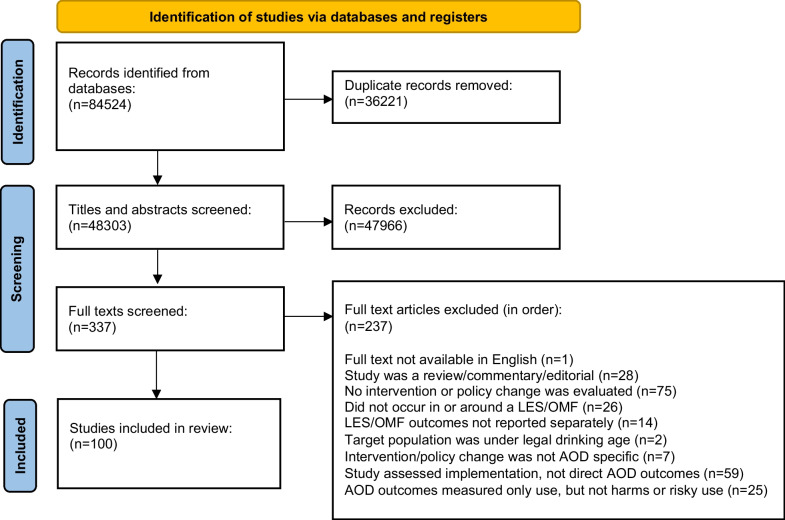
PRISMA flow diagram of study retrieval process. *Note*. LES: Licensed entertainment settings; OMF: Outdoor music festivals; AOD: Alcohol and other drugs

The literature search located 84,524 publications, with 100 included in the systematic review. The article identification and selection process are detailed in Fig. 1, following PRISMA guidelines [39].

Key characteristics of the 100 included studies are summarized in Additional file 4. A detailed overview by study is available in Additional file 5. Most studies were conducted in Australia (41%), USA (18%) or the United Kingdom (UK;18%) and focused on harm reduction interventions in LES (88%), with only 11% of studies conducted at OMF, and 1% conducted in both LES and OMF. Studies predominantly focused on reducing harms attributable to alcohol specifically (86%). The majority (59%) of studies assessed single interventions (including laws and regulations, medical services, policing strategies, chill out spaces), while the remaining 41% reported on multicomponent interventions. Of the included studies, most (84%) were strictly quantitative, 6% were strictly qualitative, and 9% used mixed methods. Most studies reported the impacts of the interventions on criminal justice outcomes (65%), followed by health-related outcomes (40%) and behavioral outcomes (20%). The methodological quality of 93 quantitative (or mixed methods) studies was assessed using the EPHPP tool (see Table 3). Of those, 26 were rated methodologically weak, 16 moderate, and 51 strong.

**Table 3:** Methodological quality ratings using EPHPP criteria

Study	Selection bias	Study design	Confounders	Blinding*	Data Collection	Withdrawals *	Global rating
Archer et al. 2012 [50]	S	W	W	N/A	S	N/A	W
Athanasopoulos et al. 2021 [51]	S	M	M	N/A	M	N/A	S
Baldin et al. 2018 [52]	S	S	S	M	M	W	M
Bassols et al. 2018 [53]	S	M	S	N/A	S	N/A	S
Bernat et al. 2013 [54]	S	M	M	N/A	S	N/A	S
Brännström et al. 2016 [55]	S	W	M	N/A	M	N/A	M
Briggs et al. 2014 [56]	S	M	M	N/A	M	N/A	S
Brown et al. 2011 [57]	M	W	W	N/A	M	N/A	W
Burgason et al. 2017 [58]	S	M	M	N/A	S	N/A	S
Carvalho et al. 2014 [59]	M	W	W	N/A	M	N/A	W
Chamlin et al. 2014 [60]	S	M	S	N/A	M	N/A	S
Charlebois et al. 2017 [61]	W	M	S	W	S	N/A	W
Coomber et al. 2021 [62]	S	M	M	N/A	S	N/A	S
Curtis et al. 2017 [63]	S	M	M	N/A	M	N/A	S
Curtis et al. 2019 [64]	S	M	M	N/A	S	N/A	S
Curtis et al. 2019 [29]	S	W	W	N/A	M	N/A	W
Curtis et al. 2019 [30]	M	W	W	N/A	M	N/A	W
Curtis et al. 2021 [65]	S	M	W	N/A	S	N/A	M
de Andrade et al. 2016 [25]	S	M	S	N/A	S	N/A	S
de Andrade et al. 2021 [66]	S	M	M	N/A	S	N/A	S
de Goeij et al. 2015 [67]	S	M	S	N/A	M	N/A	S
de Vocht et al. 2016 [68]	S	M	M	N/A	S	N/A	S
de Vocht et al. 2017 [69]	S	M	M	N/A	M	N/A	S
de Vocht et al. 2017 [70]	S	M	S	N/A	S	N/A	S
de Vocht et al. 2020 [71]	S	S	S	N/A	S	N/A	S
Devilly et al. 2019 [72]	S	W	M	N/A	M	N/A	M
Donnelly et al. 2017 [73]	S	M	M	N/A	M	N/A	S
Doran et al. 2021 [74]	S	W	W	N/A	W	N/A	W
Dutch et al. 2012 [75]	S	W	W	N/A	M	N/A	W
Fell et al. 2017 [76]	M	M	M	M	S	N/A	S
Ford et al. 2018 [77]	M	W	W	N/A	M	N/A	W
Friedman et al. 2019 [31]	S	W	W	N/A	S	N/A	W
Fulde et al. 2015 [78]	S	W	W	N/A	S	N/A	W
Garius et al. 2020 [79]	W	M	W	N/A	S	N/A	W
George et al. 2018 [80]	M	M	W	N/A	M	N/A	M
Green et al. 2014 [81]	S	M	M	N/A	S	N/A	S
Grigg et al. 2018 [82]	W	W	M	N/A	W	N/A	W
Gruenewald et al. 2015 [83]	M	W	M	N/A	M	N/A	M
Ham et al. 2021 [84]	S	S	S	M	S	S	S
Hickey et al. 2012 [85]	W	M	W	N/A	M	M	W
Hoffman et al. 2017 [86]	S	M	M	N/A	S	N/A	S
Humphreys et al. 2013 [87]	S	M	S	N/A	M	N/A	S
Humphreys et al. 2014 [88]	S	M	W	N/A	S	N/A	M
Jackson et al. 2011 [89]	S	M	S	N/A	S	N/A	S
Kazbour et al. 2010 [90]	W	W	W	N/A	W	N/A	W
Khurana et al. 2021 [91]	S	M	S	N/A	S	N/A	S
Kirby et al. 2011 [92]	M	M	M	N/A	M	N/A	M
Klein et al. 2013 [93]	S	M	M	N/A	S	N/A	S
Kypri et al. 2011 [94]	S	M	M	N/A	M	N/A	S
Kypri et al. 2014 [95]	S	M	M	N/A	M	N/A	S
Kypri et al. 2020 [96]	S	M	S	N/A	M	N/A	S
Livingston et al. 2021 [26]	S	M	S	N/A	S	N/A	S
Lund et al. 2015 [97]	S	W	W	N/A	S	N/A	W
Mazerolle et al. 2012 [98]	S	M	M	N/A	M	N/A	S
Measham 2019 [28]	S	W	W	N/A	M	N/A	W
Measham & Turnbull 2021 [99]	S	M	W	N/A	W	N/A	W
Menéndez et al. 2015 [100]	S	M	M	N/A	M	N/A	S
Menéndez et al. 2015 [101]	S	M	M	N/A	S	N/A	S
Menéndez et al. 2017 [102]	S	M	M	N/A	S	N/A	S
Miller et al. 2011 [103]	S	M	M	N/A	S	N/A	S
Miller et al. 2012 [104]	S	M	M	N/A	S	N/A	S
Miller et al. 2014 [105]	S	M	S	N/A	S	N/A	S
Miller et al. 2020 [106]	S	M	M	N/A	S	N/A	S
Monezi et al. 2017 [107]	M	M	W	N/A	W	M	W
Moore et al. 2012 [108]	M	S	S	M	M	S	S
Moore et al. 2017 [109]	S	S	M	S	M	M	S
Munn et al. 2016 [110]	M	W	W	N/A	W	N/A	W
Navarro et al. 2013 [111]	S	S	S	W	M	W	W
Nepal et al. 2019 [112]	S	M	M	N/A	M	N/A	S
Norström et al. 2013 [113]	M	M	M	N/A	M	N/A	M
Norström et al. 2018 [114]	S	M	M	N/A	M	N/A	S
Palk et al. 2010 [115]	S	W	W	N/A	M	N/A	W
Palk et al. 2012 [116]	S	M	W	N/A	S	N/A	M
Paschall et al. 2021 [117]	M	W	W	N/A	W	N/A	W
Pliakas et al. 2018 [118]	S	M	M	N/A	M	N/A	S
Ragnarsdóttir et al. 2011 [119]	S	M	W	N/A	M	N/A	M
Randerson et al. 2018 [120]	M	M	W	N/A	M	N/A	M
Rivara et al. 2012 [121]	M	W	W	N/A	W	N/A	W
Rossow et al. 2012 [122]	S	M	M	N/A	M	N/A	S
Rowe et al. 2012 [123]	M	M	W	N/A	M	N/A	M
Skardhamar et al. 2016 [124]	M	M	M	N/A	M	N/A	S
Taylor et al. 2019 [125]	S	M	M	N/A	S	N/A	S
Taylor et al. 2020 [126]	S	M	M	N/A	S	N/A	S
Taylor et al. 2021 [127]	S	M	M	N/A	S	N/A	S
Taylor et al. 2021 [128]	S	M	M	N/A	S	N/A	S
Tesch et al. 2018 [129]	M	W	M	N/A	M	N/A	M
Tomedi et al. 2018 [130]	M	M	M	N/A	M	N/A	M
Trolldal et al. 2013 [131]	S	M	M	N/A	M	N/A	S
Ward et al. 2018 [32]	S	W	W	N/A	M	N/A	W
Wiggers et al. 2021 [132]	M	M	M	N/A	M	N/A	M
Wood et al. 2010 [133]	S	W	W	N/A	W	m	W
Xu et al. 2012 [134]	S	M	M	N/A	S	N/A	S
Young-Wolff et al. 2013 [135]	S	M	S	N/A	M	N/A	S
Zawisza et al. 2020 [136]	W	W	W	N/A	W	N/A	W
Zhang et al. 2015 [137]	S	M	S	N/A	S	N/A	S

*Note*. S: Strong; M: Moderate; W: Weak; N/A: Not applicable

*Ratings for blinding and withdrawals were only applicable where the study was an RCT design

There were 13 qualitative and mixed methods studies that were assessed using the CASP tool (see Table 4). Of those, 2 were rated strong quality, 8 moderate quality, and 3 weak quality. A summary of study outcomes regarding the general direction of intervention effects (i.e., positive effect, negative effect, null effect or mixed effect) is provided in Additional file 6.

**Table 4:** Methodological quality ratings using CASP criteria

1st Author, year of study	CASP criterion											
	1	2	3	4	5	6	7	8	9	10	Total score	Quality rating
Brown et al. 2011 [57]	2	2	2	1	2	1	1	0	2	1	14	Fair
Carvalho et al. 2014 [59]	2	1	1	2	2	0	0	1	2	1	12	Fair
Farrimond et al. 2018 [138]	1	2	1	1	1	0	1	1	1	1	10	Fair
Forsyth et al. 2012 [139]	2	2	2	1	2	0	1	0	2	2	14	Fair
Hughes et al. 2018 [140]	2	1	1	2	2	0	1	0	2	2	13	Fair
Kirby et al. 2011 [92]	2	1	1	1	1	0	0	0	2	1	9	Poor
Malins 2019 [17]	2	2	2	2	2	1	1	2	2	2	18	Strong
Miller et al. 2020 [141]	2	2	1	2	2	1	1	1	2	2	16	Strong
Palk et al. 2012 [116]	1	2	2	1	2	0	0	1	1	2	12	Fair
Randerson et al. 2018 [120]	0	1	1	0	1	0	0	0	0	1	4	Poor
Ward et al. 2018 [32]	2	1	1	1	2	0	1	1	2	1	12	Fair
Zawisza et al. 2020 [136]	2	0	0	2	1	0	0	0	1	1	7	Poor

*Note.* Criteria include: 1) clear statement of aims; 2) appropriateness of qualitative methodology; 3) appropriateness of research design; 4) appropriateness of recruitment strategy; 5) appropriateness of data collection; 6) consideration of relationship between researcher and participants; 7) consideration of ethical issues; 8) rigor of analysis; 9) clear statement of findings; and 10) value of research. Scores included: “yes” = 2; “no” = 0 and “can’t tell” = 1. Overall quality ratings included: “good” (≥ 8); “fair” (5–7) or “poor” (< 5) (Casp 2013)

### Laws and regulations

There were 28 studies evaluating stand-alone interventions that included the introduction of laws or liquor licensing regulations, such as one-way door policies, changes in trading hours, liquor sale restrictions, or risk-based licensing (RBL) schemes [25, 53, 54, 56, 57, 64, 67, 81, 83, 87, 88, 91–93, 98, 104, 108, 112, 115, 116, 119, 122, 125, 129, 130, 135, 139, 141]. Of these studies, 22 were quantitative [25, 53, 54, 56, 64, 67, 81, 83, 87, 88, 91, 93, 98, 104, 108, 112, 116, 122, 125, 129, 130, 135], 4 used mixed methods designs [57, 92, 116, 119], and 2 were qualitative [139, 141]. Of the 22 quantitative studies, 17 were rated as strong using the EPHPP [25, 53, 54, 56, 64, 67, 81, 87, 91, 93, 98, 104, 108, 112, 122, 125, 135], 4 were rated as moderate [83, 88, 129, 130], and 1 was rated as weak [115]. Three of the four mixed methods studies were given a moderate EPHPP rating [92, 116, 119], and one was rated weak [57]. Eleven studies reported on health outcomes [25, 53, 54, 57, 64, 67, 81, 104, 119, 135, 141], 18 studies reported on criminal justice outcomes (particularly assaults) [25, 56, 57, 87, 88, 91–93, 98, 108, 112, 115, 116, 119, 122, 125, 129, 139], and 4 studies reported on behavioral outcomes [83, 116, 130, 139]. Additional studies examined the effectiveness of some of the stand-alone interventions listed above as part of multicomponent interventions in Sydney, Newcastle and Queensland, Australia; see multicomponent interventions sub-Sects. "Sydney “lockout laws”" and "Queensland “tackling alcohol-fuelled violence” policy". A detailed synthesis of findings for each of the main types of stand-alone interventions regarding laws and regulations is provided below.

#### Alcohol availability studies

Two strong-rated studies investigated the impacts of reduced alcohol availability [91, 125]. Different outcomes were observed. First, a study by Taylor and colleagues [125] found that a ban on the sale of “rapid intoxication liquor” (e.g., shots) after midnight in Queensland, Australia, did not affect rates of police-recorded serious assaults. Second, a study by Khurana et al. [91] investigated the impact of a ban on hard liquor sales from local bars in Kerala, India, with only five-star hotel bars permitted to sell hard liquor. The authors found that the policy had no effect on verbal insults but reduced sexual assaults significantly (but not rapes, possibly due to increased reporting). Sixteen additional studies examined the effectiveness of reduced alcohol availability as part of a multicomponent intervention in Sydney [51, 73, 78, 96, 100–102, 140], Newcastle [86, 94, 95] and Queensland [26, 62, 66, 72, 128]; see multicomponent interventions sub-Sects. "Sydney “lockout laws”" and "Queensland “tackling alcohol-fuelled violence” policy".

#### Lockout/one-way door policy studies

Five Australian studies (three rated strong [25, 98, 104], one moderate [116], and one weak [115]) investigated the impact of venue lockouts on alcohol-related harms [25, 98, 104, 115, 116]. All reported on a 3am lockout. The results were variable, including significant reductions in harms [98], significant increases [104], no change [25] and mixed effects [115, 116]. Eleven additional studies examined the effectiveness of a lockout as part of a multicomponent intervention in Sydney [51, 73, 78, 96, 100–102, 140] and Newcastle [86, 94, 95], finding significant reductions in assaults in both cities; see multicomponent interventions sub-Sects. "Sydney “lockout laws”" and “Queensland “tackling alcohol-fuelled violence” policy”.

#### Risk-based licensing (RBL) schemes

Four studies examined the impacts of RBL schemes [64, 108, 112, 141]. Two strong Australian studies [64, 112] and one strong Welsh study [108] found that RBL had no significant impact on the incidence of police-recorded assaults nor emergency department (ED) presentations for injuries during high alcohol hours (HAH; considered 8 pm–6am Friday and Saturday nights) except for a reduction in ED injury presentations among men aged 20–39 years in Victoria only [64]. These findings were generally supported by those of Miller and colleagues’ strong-rated qualitative study [141], which reported that while licensees generally supported RBL, they did not perceive RBL to have an impact on the level of alcohol-related harm and required modifications to be more effective. Twelve additional studies examined the effectiveness of liquor licensing changes as part of a multicomponent intervention [51, 63, 73, 76–78, 96, 100–102, 105, 140] see multicomponent sub-Sects. “Sydney “lockout laws””, “New Zealand’s sale and supply of alcohol act” and “Other multicomponent interventions”.

#### Change in trading hours

Ten studies investigated the impact of changes in licensed venue trading hours on alcohol-related harm outcomes [53, 57, 67, 81, 87, 88, 92, 119, 122, 129].

Five studies investigated the impacts of trading hour extensions with mixed findings [67, 81, 87, 88, 119]. Three of these studies examined the effect of 24-h trading licenses [87, 88, 119]. A moderate-rated before-and-after study [119] by Ragnarsdóttir et al. showed that extending trading hours to 24/7 in Reykjavik, Iceland’s licensed entertainment precincts increased police-reported violent offenses and ED admissions. In contrast, two studies by Humphreys and colleagues, one strong [87] and one of moderate quality [88], found that extending trading hours from 11 pm to 24/7 in Manchester, England, did not result in changes in violence, though there was some evidence of a shift in weekend violence to later hours [54]. Smaller extensions in trading hours were introduced in two strong-rated studies in Amsterdam [67] and England and Wales [81]. Findings showed contrasting results, with the extension in Amsterdam being associated with an increase in alcohol-related injuries, while England and Wales experienced a decrease in road traffic accidents, especially on Friday and Saturday nights among younger drivers.

Four other studies across Europe examined the impact of both trading hour restrictions and extensions (when closing times were staggered) following policy changes that allowed licensed venues in Preston [92] and Hartlepool [57] in England, or municipalities in Bavaria, Germany [129] or Norway [122] to determine venue opening and closing hours. Two moderate-rated studies [92, 129] and one weak quality study [57] found that when these policies led to a slight increase in trading hours, there was an observed decrease in alcohol-related crimes [92]; violence against the person [57, 129] and criminal damage [57]. In contrast, Rossow and colleagues’ [122] strong-rated study reported a statistically significant increase in assaults following closing hour extensions in Norway. Brown and colleagues [57] also reported an increase in the number of ambulance attendances and public order offenses (antisocial behavior) in England. Furthermore, Tesch and colleagues [129] found that restricted trading hours led to an increase in night-time violence in Bavarian towns with higher levels of day-time violence.

A further strong study in Spain found reduced trading hours linked to decreased alcohol-related hospitalizations [53].

#### Legal purchase age

One moderate-rated study examined the impacts of a legal purchase age policy. A before-and-after study design was used to examine the impact of a reduction in the legal purchase age for alcohol from 20 to 18 years in New Zealand [83]. Finding showed that the policy resulted in a threefold increase in rates of problems per drinking occasion at pubs and nightclubs, across all drinking quantities.

#### Smoking ban

Five studies described various impacts of smoking bans in licensed venues, including four strong-rated quantitative studies from the USA [54, 56, 93, 135] and one fair rated observational study from Scotland [139]. Young-Wolff and colleagues [135] reported the only positive health outcome, with higher likelihood of alcohol use disorder remission following the introduction of a ban [135]. Two studies reported a decline in violence/assaults following a ban [56, 139], while one study reported no impact on the rate of assaults [93]. Mixed results were also reported regarding sexual assaults, with Forsyth and colleagues [139] reporting an increase in sexual assaults in Scotland, while no change was reported following the introduction of a ban in Minnesota, USA. [93]. Forsyth and colleagues [139] also reported mixed findings for behavioral outcomes, observing an increase in risky alcohol consumption practices, and a decrease in overcrowding in licensed venues following a ban. Null findings were reported in USA-based studies for traffic-related health outcomes (alcohol-related road traffic accidents or car crash fatalities, [54]) and other crime outcomes (general crime, or public order offenses [93]).

#### Regulatory compliance and enforcement

A moderately rated before-and-after study by Tomedi and colleagues [130] found that enhanced enforcement of laws prohibiting the sale of alcohol to intoxicated patrons in on-premised licensed venues significantly reduced the proportion of adults that engaged in risky consumption practices (i.e., drinking eight or more standard drinks) in these settings during their most recent drinking episode in New Mexico, USA. Six additional studies examined the effectiveness of enhanced regulatory compliance and enforcement as part of a multicomponent intervention [55, 71, 113, 131, 134, 137]; see Sects. “UK multicomponent interventions” and “SALUTT and STAD interventions in Northern Europe”.

### Drug checking/pill testing

Two cross-sectional weak-rated studies by Measham examined the effectiveness of drug checking at OMFs in the UK [28, 99]. Findings indicated that drug checking was associated with reduced dose size and increased rates of the disposal of dangerous drugs [99], as well as a 95% reduction in AOD-related hospital presentations [28]. One additional study examined the effectiveness of drug checking as part of a multicomponent intervention at a OMF [110]; the results of this study are described in the ‘Multicomponent intervention’ Sect. “Other multicomponent interventions”.

### Transport interventions

There were six studies that examined the effectiveness of transport interventions [29, 30, 89, 90, 106, 121]. Three studies examined the impact of extending public transport hours, including one strong-rated study that investigated the impacts of a gradual extension of public transport operating hours in Washington DC in the USA from midnight to 3am [89], and two weak-rated studies by Curtis and colleagues [29, 30] which investigated the impacts of 24-h public transport in Melbourne, Australia. Findings showed that extending public transport hours had limited impact on a range of outcomes including driving under the influence (DUI) arrests (however areas with bars within walking distance to transit stations noted decreases in DUI arrests), total alcohol-related arrests for public nuisance and disorder [89], alcohol-related injuries, police-recorded assaults, ambulance attendances, and road traffic accidents on Friday and Saturday nights [29], with some evidence of displacement of road crashes over adjacent hours early Sunday morning rather than an impact of the intervention [29]. Three studies based in the USA examined the effects of an intervention that included a designated driver/ride sharing measure. One weak-rated study investigating the effects of designated drivers at a college bar in Tallahassee, and one strong-rated study in Ohio that evaluated a ride sharing service found such measures led to an increase in the proportion of patrons riding with/acting as a designated driver (12–24%; [90]), or stating that they would be less inclined to drive after drinking (71%; [106]). Studies reported mixed results regarding any changes in the number of alcohol-attributable crashes (or single vehicle night-time crashes as a proxy) following the interventions. While Miller and colleagues [106] reported no significant change following a rideshare promotion, Rivara et al. [121] reported a substantial reduction in single vehicle night-time crashes among 21–34-year-old drivers as part of their weak-rated study, conducted in Seattle, USA that consisted of a multi-pronged social marketing campaign promoting taxi use and designated drivers. Two additional studies examined the effectiveness of safe taxi ranks (i.e., ranks manned by security staff) as part of a multicomponent intervention [63, 105]. The results of these studies are described in the ‘Multicomponent intervention’ Sects. “UK multicomponent interventions” and “SALUTT and STAD interventions in Northern Europe”.

### Policing strategies

There were six studies that examined the effectiveness of policing strategies [17, 65, 82, 85, 123, 127]. Studies were limited to examining the effects of strategies on criminal justice and behavioral outcomes (i.e., not health outcomes). Three cross-sectional studies (two rated weak [82, 85] and one strong [17]) examined the impacts of drug detection dogs at OMF or LES in Australia. Each showed the presence of drug detection dogs led to increased risky consumption and behaviors, with 10% [82] to 36% [85] of participants reporting they concealed drugs internally, or ‘panic’ consumed some/all their drugs in response to the presence of drug detection dogs [17]. In addition, Hickey and colleagues [85] reported that 13–43% of participants (among different subgroups) had been arrested, cautioned, or fined following positive notifications from drug detection dogs.

Two studies examined the effects of police-imposed bans within licensed entertainment precincts on criminal justice outcomes [65, 127]. The first study by Curtis et al. [65], which had a moderate rating, reported a significant increase in antisocial behavior charges, specifically public order offenses, following the introduction of 72-h police-imposed bans in Victoria, Australia. In contrast, a strong-rated study investigating the impact of 10-day police-imposed bans, along with mandatory ID scanners, in three licensed entertainment precincts in Queensland found no evidence of changes in serious assaults, common assaults, or offenses related to public order/good order [127]. A further moderate-rated study by Rowe et al. [123] found that one of three levels of policing enforcement responses (letters, incident reports or feedback meetings) was associated with significant reductions in police incidents and rates of intoxicated patrons in high-risk licensed venues in regional New South Wales, Australia.

### Medical services

There were five weak-rated studies that examined the impact of medical services at OMFs on health outcomes in Australia [75], Canada [97], the UK [50, 133] and the USA [31]. Four were cross-sectional studies [31, 75, 97, 133] and one was a prospective cohort study [50]. Four out of the five studies [31, 50, 75, 97] demonstrated a significant reduction in ambulance transfer rates by 65–78%, due to the availability of medical personnel on site at the OMF. Similar reductions were reported by Wood and colleagues [133] who compared transfer rates from an OMF and the OMF after party event, reported 45% less transfers for AOD toxicity from the after party, where an ambulance treatment tent was present [133].

### Chill/safe spaces and roaming support services

There were five studies that examined the effectiveness of chill/safe spaces and roaming support services, finding mixed impacts on criminal justice and health outcomes [32, 59, 74, 79, 126]. Four were rated as weak [32, 59, 74, 79] and one as strong [126]. Two Australian studies showed that the introduction of a chill/safe space [32] or roaming street support services [126] in licensed entertainment precincts had no significant impact on hospital admissions [32, 126] or ambulance attendances [126]. The impact of support services on assaults in Australian cities was mixed, with some evidence of significant reductions in general and sexual assaults in Sydney [74] and serious assaults during HAH in Cairns, [126], while Ward and colleagues [32] reported an increase in violence in taxi queues in Melbourne. Doran and colleagues [74] also reported a reduction in road traffic accidents (12%) and thefts (40%), while Ward [32] found no significant association between the introduction of a mobile van and chill/safe space and the proportion of police-reported incidents (public order, theft, property damage or drug offenses) during HAH. In contrast, an evaluation of a roaming support service in licensed entertainment precincts in two cities in the UK found the introduction of the service was associated with an increase in police-recorded crime levels (including violence and sexual assaults) [79]. The only study to examine a safe space initiative at a multiday festival was in Portugal by Carvalho and colleagues [59], which found that patrons presenting to the service with difficult psychedelic and emotional experiences showed a significant positive effect on resolving their mental health episode (*p* < 0.05), thus confirming crisis resolution.

### Staff and venue interventions

There were five studies that examined the effectiveness of staff and venue interventions across a range of late-night entertainment settings [61, 84, 109, 136, 138], showing conflicting impacts on a range of outcomes. Two strong-rated studies by Ham et al. [84] and Moore et al. [109] examined the impact of staff training in responsible service of alcohol (RSA) and conflict resolution, showing no significant change in the number of assaults [84] and an increase in reported assaults following visits and risk audits by Environmental Promotion Officers [109]. Ham et al. [84] also reported no statistically significant changes in the number of reported brawls but did find a decrease (16%) in reports of public disorder, with no evidence of displacement. In a weak-rated study by Charlebois and colleagues [61], the promotion of free water led to a reduction in incidence of hazardous drinking (78%), compared to the control bar participants (87%).

A qualitative moderate-rated study explored the impacts of the voluntary adoption of hand-held breathalyzers on the doors of licensed venues in the UK [138]. Mixed findings were reported, with some security staff reporting reduced aggression at the door, while others reported that the intervention was not useful. Finally, a weak-rated observational study conducted by Zawisza et al. [136] [136]examined the impact that the presence of place managers and capable guardians (e.g., police, security staff, bar staff) in licensed entertainment precincts (Arkansas, USA) had on incidents of aggression. The authors observed a reduction in the number of incidents of aggression during a 6-week period [136].

### Patron surveys and assessment feedback

There were two studies that examined the impact of patron surveys and assessment feedback in licensed entertainment precincts on risky consumption practices [52] and driving offenses [107]. The moderate-rated RCT study by Baldin and colleagues [52] found that their web-based survey with personalized normative feedback on alcohol-related risks and tips to reduce harms led to a significant reduction in weekly binge drinking among participants at six-month follow-up of 38% (*p* = 0.026); however no significant effect was observed for the control group. In contrast, the weak-rated study by Monezi and colleagues [107] found that their patron survey (which included a module on how alcohol consumption affects driving capacity) had no significant impact on drink driving behaviors.

### Multicomponent interventions

There were 41 studies that examined the effectiveness of multicomponent interventions that utilized at least two or more strategies of those outlined above [26, 51, 55, 58, 60, 62, 63, 66, 68–73, 76–78, 80, 86, 94–96, 100–103, 105, 110, 111, 113, 114, 117, 118, 120, 124, 128, 131, 132, 134, 137, 140]. A multi-pronged approach typically requires a program of multiple, co-ordinated measures rather than ‘stand-alone’ interventions, and an emphasis on encouraging change in local policies, structures, systems, and AOD use cultures. Twenty-nine studies were rated as strong [26, 51, 58, 60, 62, 63, 66, 68–71, 73, 76, 86, 94–96, 100–103, 105, 114, 118, 124, 128, 131, 134, 137], six as moderate [55, 72, 80, 113, 120, 132], and five as weak [77, 78, 110, 111, 117]. One qualitative study was rated as fair [140]. Most notable are the three major multicomponent interventions that have been conducted in Sydney, Newcastle and across Queensland, Australia, with comprehensive evaluations of strong quality showing all to be largely effective in reducing police-recorded assaults, hospitalizations, and ambulance callouts. Only one study in this category examined the effect of a multicomponent measure at a festival [110].

#### Sydney “lockout laws”

Eight studies reported on the effectiveness of the “lockout laws” in Sydney, Australia [51, 73, 78, 96, 100–102, 140]. Of these, six strong-rated studies examined changes in police-reported assaults, all showing statistically significant declines over time that ranged from 22 to 49% [51, 73, 96, 100–102]. Two of the more recent studies showed that the declines in violence in Sydney CBD and Kings Cross were sustained between 2014 and 2021 [51, 96]. The decline in assaults was also reflected in a decline in hospitalizations, as reported in Fulde and colleagues’ [78] weak-rated study. This declining trend in violence was echoed by residents, patrons and music industry stakeholders in the lockout zone during focus groups with Hughes and colleagues [140] as part of their moderate-rated qualitative study. However, participants also reported possible displacement of violence to adjacent suburbs. Evidence of violence displacement was also found in a strong-rated quantitative study by Donnelly et al. [73] with a 12% increase in assaults within the Proximal Displacement Areas, and a 17% increase in assaults within the Distal Displacement Areas. The displacement was significant and increased over time, albeit with an overall net reduction of non-domestic alcohol-related violence across target and adjacent precincts [73].

#### Queensland “tackling alcohol-fuelled violence” policy

Five studies evaluated the effectiveness of the TAFV Policy implemented in SNPs in Queensland, Australia, in reducing alcohol-related harms [26, 62, 66, 72, 128]. Four strong studies indicated a significant reduction in the number of assaults [62, 128], hospitalizations [26], and ambulance callouts [66]. The moderate quality study by Devilly and colleagues [72] reported no meaningful change in public order offenses and an increase in drug offenses.

#### Newcastle liquor licensing restrictions

Five studies evaluated the effectiveness of legislative changes in Newcastle, Australia [86, 94, 95, 105, 132]. Four were rated strong [86, 94, 95, 105] and one moderate-rated study [132] assessed community perceptions and experiences of crime, as well as support for restrictions. Miller et al. [105] compared the Newcastle CBD intervention with Geelong’s own set of interventions which included: Geelong—(I) ID scanners, (ii) increased policing and CCTV, (iii) taxi ranks, and (iv) risk-based licensing. Similarly, Kypri et al. [95] compared the Newcastle intervention with Hamilton. The legislative changes led to significant long-term declines in police-recorded assaults [94, 95] and alcohol-related injury presentations to hospital [86, 105]. Comparison to a control site found that reduced trading hours rather than the lockout was the key mechanism to change [95]. Results of a survey of Newcastle residents further supports these findings, highlighting a significant decrease in general crime, risky alcohol consumption and witnessing or being involved in physical violence [132].

#### US multicomponent interventions

Six studies reported on multicomponent interventions in the USA [58, 60, 76, 80, 134, 137]. Multicomponent interventions trialled in the USA have largely been designed with RSA and law/liquor licensing enforcement measures at their core. In Zhang and colleagues’ strong study [137] they reported a reduction in violent crime in Atlanta, USA, following the introduction of restricted trading hours combined with a reduced density of alcohol outlets, and the enforcement of laws prohibiting alcohol sales to minors. In contrast, two strong studies examined the impact of increased trading hours combined with enhanced law enforcement in licensed entertainment precincts in San Marcos, Texas, USA [60] and Little Rock, Arkansas, USA [58]. While Chamlin et al. [60] found the intervention to have no effect on public order offenses or complaints of DUI, Burgason et al. [58] reported a 23% reduction in reported violent crime with their intervention also including improved lighting and video surveillance and increased penalties.

Three studies based in the USA examined road traffic accidents and DUI related arrests and accidents following interventions that focused on improving RSA training and increasing onsite/liquor licensing law enforcement. In Fell and colleagues’ [76] strong-rated before-and-after controlled study targeting problem bars in New York and Ohio, they found no statistically significant changes in road traffic accidents pre-post study or between intervention and control bars, However, findings showed a decline in reported DUI arrests post-intervention and a significant short-term decline in self-reports of drink driving in New York. Similarly in South Carolina, USA, a moderate-rated non-randomized controlled trial which comprised of a RSA practices toolkit, onsite law enforcement and a media campaign in licensed entertainment precincts, resulted in a significant reduction in alcohol-involved crashes and DUI arrests in the year following the intervention [80]. Similar to Fell and colleagues’ [76] study, Xu et al.’s [134] strong-rated study targeted problem alcohol outlets in New Orleans. Additional enforcement staff combined with increased license fee and expanded powers for the alcohol license board were found to have no impact on the crime rate over two years post-intervention [134].

#### New Zealand’s sale and supply of alcohol act

One weak study [77] and one moderate study [120] examined the impact of the Act on ED attendances [77] and drink driving [120]. The cross-sectional study by Ford et al. [77] reported a non-significant reduction in ED attendees, while Randerson et al. [120] found a 24% reduction in apprehension of drivers with a BAC of 0.008, and 35% drivers reported drinking less before driving after the law change.

#### UK multicomponent interventions

Five strongly rated studies, all conducted in UK, examined the impact of introducing cumulative impact zones (CIZs) and the impact of implementing new local licensing guidelines and increased venue inspections [68–71, 118]. The study designs included data linkage methods [68], a quasi-randomized control trial [71], a before-and-after study [69] and two interrupted time series (ITS) analyses [70, 118]. CIZs were found to have a positive impact on alcohol-related hospital admissions and crime, particularly alcohol-related violent crimes and sexual assaults (but not public order offenses). For example, one strong before-and-after study by De Vocht et al. [69] examined the impact on alcohol-related hospital admissions, reporting reductions following the introduction of CIZs and intense liquor license scrutiny, measured as the number of refusals of new liquor license applications. In Pliakas and colleagues’ [118] strong-rated study, they found no impact on alcohol-related ambulance callouts. The quasi-randomized control trial [71] found that closing a nightclub and improvements in liquor licensing guidance and inspections were collectively associated with a reduction in antisocial behavior/public order incidents, but not other crime (including assaults) or acute health service attendance.

#### SALUTT and STAD interventions in Northern Europe

Five studies evaluated the STAD and modified versions such as SALUTT, aimed at reducing alcohol-related harms in LES [55, 113, 114, 124, 131]. The moderate- and strong-rated ITS studies evaluating STAD in Stockholm and Visby in Sweden both reported significant reductions in police-recorded violence in the entertainment precincts of 29% and 70%, respectively [113, 114]. This was despite the closing time in Visby being extended from 2 to 3am. These findings were supported by the work of Trolldal et al. [131] and Brännström et al. [55], who conducted strong- and moderate-rated studies examining the impact of STAD (and modified versions) much larger areas of Sweden (237 and 288 municipalities, respectively). Both studies found STAD led to significant reductions in police-recorded assault. However, Trolldal and colleagues [131] identified the implementation of the community coalition steering group as the key driver of the reduction (rather than the RSA training or supervision components of the intervention). In contrast to findings of the STAD studies, a strong-rated evaluation found the SALUTT intervention to have no statistically significant effect on violence in and around licensed premises [124].

#### Other multicomponent interventions

Two strong-rated studies in Victoria, Australia, examined the impact of multi-pronged community-based interventions on ED presentations [63, 103] and police-recorded assaults [63]. While the Fremantle liquor accord regulations included a broad range of measures including patron bans, RSA guidelines, one-way door policy, awareness campaigns, liquor license freeze, safe taxi rank, policing operations, night watch radio program, ID scanners, and RBL [63], the Geelong-based intervention included measures more heavily focused on increasing licensed venue and police collaboration and enforcement. ITS analyses showed that neither intervention had a significant effect on the count of assaults, or rate of ED presentations, at any research sites during peak weekend times. While findings by Miller et al. [103] indicated that ID scanners and a safe drinking awareness campaign were associated with an increase in ED attendances, authors suggest these findings to be coincidental rather than correlational.

A weak-rated study by Navarro et al. [111] examining a multi-stakeholder/multicomponent intervention in regional NSW that included a letter from the mayor to licensees asking to brief security staff and ensure RSA, local media briefings, increased police visibility, incident feedback through media and stakeholder meetings. The intervention had no effect of the intervention on assaults but a small statistically significant effect on alcohol-related sexual assaults.

A weak-rated study in Mexico found reduced trading hours linked to decreased alcohol-related assaults [117]. The intervention involved a combination of restricted bar opening hours and the introduction of a 10PM cut-off for alcohol sales by off-premise stores.

One weak study examined the effect of a multicomponent intervention at a festival, on both alcohol and illicit drug harms, reporting a reduction in ambulance attendances and acuity, and a decrease in the utilization for local, community-based health services [110]. It is important to note that isolating specific outcomes for individual interventions within multicomponent studies, such as Munn and colleagues [110], can be challenging due to their inherent design focus on collective effectiveness rather than singular components.

## Discussion

This systematic review synthesized the impact of interventions on AOD-related harms in LES and OMF alongside their impacts on health, criminal justice, and behavioral outcomes. A dominant majority of studies focused on interventions in LES (88%) when compared to OMF settings (11%), or both (1%). A similar majority of studies examined reducing harms associated with alcohol consumption (86%) compared to illicit drug consumption (7%) or both (7%). Further, multicomponent interventions (39%) were common compared with stand-alone interventions such as policing strategies (6%), medical service provision (5%), chill spaces and roaming support services (5%), and drug checking services (2%), and there was a considerably stronger emphasis on evaluating criminal justice outcomes (65%) than health outcomes (40%) and behavioral outcomes (18%).

This review provides promising evidence into the effectiveness of different interventions that may impact AOD-related harms, including highlighting what works and what does not. Multicomponent interventions showed the best results for both health and criminal justice outcomes, with 28/39 (72%) of studies being of strong quality, and 33/39 (85%) of studies reporting at least one positive outcome (e.g., decreased hospitalizations and assaults). Further, the evidence available regarding medical services and drug checking at OMF, while limited, suggests that they are promising approaches to decrease hospitalizations, ambulance attendances and the overall burden on local healthcare infrastructure. There was also good evidence to support the careful application of trading hour restrictions [53, 115–117, 122], but less so for staggered closing times [88, 92], mandatory ID scanners and police-imposed patron bans [65, 127].

When applied as standalone measures, laws and regulations interventions showed mixed effects (see Additional file 6), with some unintended consequences. There was no supporting evidence for the use of police-issued patron bans [65, 127], however those examined were short-term and the examination of longer-term bans is required. There was also no supporting evidence for the use of drug detection dogs [17, 82, 85]: a significant finding given their increased use across many countries with the aim of managing public safety. The literature canvassed in this review suggests that LES patrons and festivalgoers often engage in risky drug consumption practices when police and dogs are present, due to fear of criminal justice repercussions, with their presence also acting as a barrier to medical help-seeking [17, 82, 85].

This review builds on the findings of prior reviews in the field. Reviews from over a decade ago (for example see Akbar et al. [15]) identified this topic as one that is under-researched. However, the large number of studies identified through this review highlight a period of intense research investment since the review by Akbar and colleagues [15], which only included 14 studies, provides promising evidence on some interventions and services, and consolidates the evidence on several interventions previously tagged as “promising,” such as multicomponent interventions. This review also highlights the significant increase in high-quality research that has been conducted in recent years in entertainment precincts, and the continued lack of research (including high-quality research) being conducted in other high-risk settings such as OMF.

This review has highlighted continued gaps in the existing evidence base. Of note is the heavy focus on: 1) harm associated with LES (with few in OMF settings), 2) alcohol-related harm (rather than other drug-related harm), and 3) criminal justice outcomes (46%), specifically assault and violence. This focus is problematic as it reduces the capacity to assess impacts on health or across multiple outcome domains. More broadly, we see considerable variability in outcomes used by different studies, reducing the capacity to conclusively identify whether the interventions reduce harm, in what ways and if there are any unintended effects. Improving the study designs such that future harm reduction evaluations assess impacts across multiple outcome domains (health, criminal justice and behavioral) and using consistent and high-quality research methods is recommended. Furthermore, while studies focused on the evaluation of medical service provision at OMF demonstrated promising findings, there is a clear need for more high-quality research to be conducted in this domain.

In addition, this review highlights the volume of high-quality evaluations of multicomponent interventions implemented in LES. While these interventions are largely proving successful, there is heterogeneity regarding the measures included in multicomponent interventions internationally. This heterogeneity, and the implementation design, results in an inability to decipher the key drivers of the success. Adding to this ambiguity is the conflicting evidence regarding some of the measures included in multicomponent interventions when delivered as stand-alone interventions. This highlights the need for more comprehensive evaluations of multicomponent interventions to identify what works to improve outcomes, how and for whom.

### Limitations

This review has four main limitations. First, we acknowledge the extensive heterogeneity in study designs, strategies, sample sizes, and outcomes, which has been a significant factor in our methodology and analysis. This diversity, encompassing both quantitative and qualitative research methodologies, presented unique challenges in synthesizing the data, consequently preventing a meta-analysis from being conducted. We have intentionally included a broad range of interventions, study designs, and impacts to capture the comprehensive scope of the subject matter. However, this inclusivity also brought with it challenges of balancing and weighing the contributions of diverse study types. This aspect, in particular, should be considered when interpreting the findings of our review. We refer the reader to our earlier discussion where we justify the inclusion of this wide spectrum of studies and acknowledge the implications of their heterogeneity on our results. Second, the study was limited by design to English language publications only. Third, an overwhelming majority of studies were conducted in developed countries (*n* = 91) such as Australia, the USA or the UK which have more similar drinking cultures. This means that we are unable to explore differences in cultural norms that may affect AOD use and harms in different milieus particularly beyond the Global North. Moreover, including publications only in English and largely from developed countries impacts the generalizability and translatability of the research findings. Fourth, this review did not include grey literature, potentially increasing publication bias given researchers may be less inclined to publish null or negative findings in academic journals [142]. However, approximately half of all health, criminal justice and behavioral outcomes reported in studies in this review found null and negative effects (see Additional file 6), suggesting that publication bias is likely to be minimal.

### Implications for policy and practice

This review provides an up-to-date summary for policymakers of what works in LES and OMF to reduce AOD-related harms. Firstly, it highlights that there are interventions that can be deployed in LES and OMF to improve health, criminal justice and behavioral outcomes—whether they be overdose, injuries, mental health conditions, hospitalizations, assaults, or road traffic accidents. Secondly, it highlights the benefits of maintaining and expanding investment in key harm reduction approaches, namely onsite medical services and multicomponent interventions. Based on results presented in this review, each of these intervention types can be deemed as effective tools for harm reduction. Thirdly, findings of this review highlight a number of areas where caution is recommended. These include the deployment of drug detection dogs at OMF, whereby their presence was found to be related to increase risky consumption practices and potential harms. Finally, this review clearly demonstrates the importance of continuing to invest in high quality study design and evaluations to ensure that harm reduction interventions are effective.

## Conclusion

To the best of our knowledge, this is the most up-to-date and comprehensive review of strategies that aim to impact AOD-related harm in LES and OMF around the world. Findings provide consistent support for onsite medical services for reducing ambulance transfer rates, multicomponent interventions targeting alcohol accessibility and availability for reducing assaults, and promising behavioral outcomes for patrons who use drug checking services. Furthermore, findings of this review suggest that policing strategies rarely reduce criminal justice outcomes and, in some instances, lead to an exacerbation of negative health outcomes. This review thus holds clear implications for policy and practice in these settings, and highlights the need for studies that address identified evidence gaps in OMF settings, around illicit drugs more broadly and in the Global South. Continuing to grow this evidence is important as harm reduction measures become increasingly prevalent across the world.

### Supplementary Information

**Additional file 1:** Review findings summary (2010-2021) for studies reporting health, criminal justice and behavioral outcomes of harm reduction strategies in licensed entertainment settings and outdoor music festivals.

**Additional file 2:** PRISMA 2020 Checklist.

**Additional file 3:** Database search strings.

**Additional file 4:** Descriptive statistics for studies included in review (*n* = 100).

**Additional file 5:** Summary of studies included in the review on the effectiveness of AOD harm reduction strategies in licensed entertainment settings and outdoor music festivals (2010–2021) (*n* = 100), grouped by intervention type.

**Additional file 6a:** Health Outcome Effects. **6b:** Criminal Justice Outcome Effects. **6c:** Behavioural Outcome Effects.

## Data Availability

The dataset supporting the conclusions of this article is included within the article’s additional files.
