# Supplementary material for: A systematic review of interventions that impact alcohol and other drug-related harms in licensed entertainment settings and outdoor music festivals
Source: Harm Reduct J. 2024 Feb 21;21:47. doi: 10.1186/s12954-024-00949-4 (PMC10882826; doi:10.1186/s12954-024-00949-4)
Supplement: Supplementary file 1 — Additional file 1: Review findings summary (2010-2021) for studies reporting health, criminal justice and behavioral outcomes of harm reduction strategies in licensed entertainment settings and outdoor music festivals. [file 12954_2024_949_MOESM1_ESM.docx]

**Additional File 1. Review findings summary (2010-2021) for studies reporting health, criminal justice and behavioral outcomes of harm reduction strategies in licensed entertainment settings and outdoor music festivals.**

| **Study** | **Intervention details** | **Studies/**  **designs** | **Outcomes** | | | **Outcome/conclusion**  **details** | **Identified gaps** |
| --- | --- | --- | --- | --- | --- | --- | --- |
|  |  |  | ***Health*** | ***Criminal Justice*** | ***Behavioural*** |  |  |
| Akbar et al. (2011) (1)  Systematic review  1998 – 2010  **Poly-substance use and related harms: A systematic review of harm reduction strategies implemented in recreational settings** | *Intervention:* Community programmes and in recreational settings addressing alcohol and drug-related harm.  *Setting:* late-night licensed venues and festivals | 14 studies,  5/14 (1 RCT) were controlled | Hospital and ED admissions | Assaults | Nil | Unclear | Polysubstance use, No risk-of-bias or methodological quality assessment conducted, outdated, poor quality studies, lacks clarity on outcomes, no synthesis of findings, 86% of studies focused on RSA training |
| Bolier et al. (2011) (2)  Systematic review  1990 – 2010  **Alcohol and drug prevention in nightlife settings: a review of experimental studie**s | *Intervention:* community interventions, RSA training, education, policy; addressing alcohol and drugs  *Setting:* late-night licensed venues. | 17 studies, experimental designs  10/17 (4 RCT) were controlled | Risky drinking, injury | Violent crime | Underage drinking, Service to intoxicated patrons | Community interventions can have preventative effect; policy RSA interventions effective when enforced.  Need more evaluations of preventive drug interventions, and AOD education interventions | Quality of studies not high; Doesn't cover festivals, only nightlife settings, outdated, studies predominantly took place in USA and Australia |
| Jones et al. (2011) (3)  Systematic review  1990 –July 2008  **Reducing harm in drinking environments: A systematic review of effective approaches** | *Intervention:* Interventions in bar setting - RSA, patron interventions, policing and enforcement, multi-component  *Setting:* late-night licensed venues. | 39 studies, 23 studies rated with EPHPP (14 RCT or CCT) | Injuries, pedestrian injuries, health service utilization, excessive alcohol consumption | Violence, assaults, aggression, crime, road traffic crashes, underage sales |  | Multi-component interventions can result in positive effect across a range of outcomes. | Outdated, alcohol only, limited studies of strong quality. |
| Liu et al. (2016) (4)  **Systematic review of Australian policing interventions to reduce alcohol-related violence - A maxillofacial perspective** | *Intervention:* Policing interventions targeting alcohol-related violence (falling into one of the three categories: front-line strategy; monitoring, regulation and enforcement strategy; and collaborative partnership) *Setting:* night-time economy settings in Australia | 10 studies, 10 studies rated with EPHPP  6/10 (2 RCT) were controlled studies | Hospital admissions, hospital injury-related presentations | Police-recorded assaults | 1 study: AUDIT scores, binge drinking rates | The overall evidence base to support Australian policing interventions was found to be poor and was limited by the low-quality study design observed in the majority of the included studies. However, there is some evidence to suggest interventions involving proactive policing to be more effective than traditional reactive policing. | The overall quality of the included studies was poor with seven studies rated poor, two moderate and one strong. The study was generally limited to 2 outcomes – hospital admissions, and assaults. |
| Nepal et al. (2018) (5)  Introduction of Lockouts – 1^st^ June 2017  **Effectiveness of lockouts in reducing alcohol-related harm: Systematic review** | *Intervention*: lockouts *Setting:* licensed premises in Australia | 8 studies, 8 studies assessed with Cochrane Effective Practice and Organization of Care Guide. No RCTs (7 before-and-after, 1 ITS) | ED attendances | Assaults, Drink-driving offence*s* | Alcohol-related disorders | There isn’t good evidence that lockouts prevent alcohol-related harm. Research concerning the effectiveness of lockouts is inconclusive. | Only 8 studies included. The study was limited to lockouts in licensed venues only. 1 study rated as high risk of bias due to displacement, 3 rated as high risk of bias due to confounding, 3 rated as high risk of bias due to no adjustment for seasonality. |
| Taylor et al. (2018) (6)  Peer Reviewed: 2003 - 27th of March/3rd of April 2017  Grey Lit - 2003- 30th of June and the 7th of July, 2017  **A mapping review of evaluations of alcohol policy restrictions targeting alcohol-related harm in night-time entertainment precincts** | *Intervention*: Alcohol restrictions (outlet density, trading hours, lockouts, price restrictions, patron bans, restrictions on drink types for sale) *Setting*: Night-time entertainment precincts | 48 studies. No quality assessment was conducted. | Injury rates | Assault rates |  | Outlet density, trading hours, and price restrictions all had evidence that suggested high levels of effectiveness in NEPs and would be suitable for inclusion in a theoretical model. | Studies limited to night-time entertainment precincts. Only measured injury and assault rates. No risk-of-bias or methodological quality assessment conducted; only quantitative studies included. |
| Wilkinson et al. (2016) (7)  **Impacts of changes to trading hours of liquor licences on alcohol-related harm: a systematic review 2005-2015** | *Intervention:* Changes to trading hours *Setting:* Licensed venues and off-premises outlets | 21 studies – 10/21 were controlled studies. Zero RTCs | Motor vehicle crashes, alcohol-related morbidity, general injury | Drink driving, violence, assault |  | A series of robust, well-designed studies from Australia demonstrate that reducing the hours during which on-premises alcohol outlets can sell alcohol late at night can substantially reduce rates of violence. | In several cases, studies that used a before and after design did not collect information on how widespread the actual implementation of permitted extensions in closing times was. Most studies did not include control sites or measures. Differences in study results appeared to be more strongly related to the specific setting than the study quality. Most of the studies from the past 10 years have focused on urban areas |

References

1. Akbar T, Baldacchino A, Cecil J, Riglietta M, Sommer B, Humphris G. Poly-substance use and related harms: A systematic review of harm reduction strategies implemented in recreational settings. Neurosci Biobehav Rev. 2011;35(5):1186-202.

2. Bolier L, Voorham L, Monshouwer K, van Hasselt N, Bellis M. Alcohol and drug prevention in nightlife settings: A review of experimental studies. Subst Use Misuse. 2011;46(13):1569-91.

3. Jones L, Hughes K, Atkinson AM, Bellis MA. Reducing harm in drinking environments: A systematic review of effective approaches. Health & Place. 2011;17(2):508-18.

4. Liu T, Ferris J, Higginson A, Lynham A. Systematic review of Australian policing interventions to reduce alcohol-related violence - A maxillofacial perspective. Addict Behav Rep. 2016;4:1-12.

5. Nepal S, Kypri K, Pursey K, Attia J, Chikritzhs T, Miller P. Effectiveness of lockouts in reducing alcohol-related harm: Systematic review. Drug Alcohol Rev. 2018;37(4):527-36.

6. Taylor N, Miller P, Coomber K, Mayshak R, Zahnow R, Patafio B, et al. A mapping review of evaluations of alcohol policy restrictions targeting alcohol-related harm in night-time entertainment precincts. Int J Drug Policy. 2018;62:1-13.

7. Wilkinson C, Livingston M, Room R. Impacts of changes to trading hours of liquor licences on alcohol-related harm: A systematic review 2005-2015. Public Health Res Pract. 2016;26(4):Article 2641644.
