# Supplementary material for: A systematic review of interventions that impact alcohol and other drug-related harms in licensed entertainment settings and outdoor music festivals
Source: Harm Reduct J. 2024 Feb 21;21:47. doi: 10.1186/s12954-024-00949-4 (PMC10882826; doi:10.1186/s12954-024-00949-4)
Supplement: Supplementary file 3 — Additional file 3: Database search strings. [file 12954_2024_949_MOESM3_ESM.docx]

**Additional File 3 – Database search strings**

**PubMed/Medline**

| 1 | (Drug use OR substance use OR illicit drug* OR illegal drug* OR narcotic* OR recreational drug* OR street drug* OR alcohol OR polydrug) |
| --- | --- |
| 2 | (Designer drug* OR club drug* OR legal high* OR stimulant* OR hallucinogen*) |
| 3 | (Alcohol[Title/Abstract] OR beverage*[Title/Abstract] OR liquor*[Title/Abstract] OR binge*[Title/Abstract] OR drunk[Title/Abstract]) |
| 4 | (Ecstasy[Title/Abstract] OR MDMA[Title/Abstract] OR mushrooms[Title/Abstract] OR psilocybin[Title/Abstract] OR LSD[Title/Abstract] OR amphetamines[Title/Abstract] OR dexamphetamine[Title/Abstract] OR methamphetamine[Title/Abstract] OR cocaine[Title/Abstract] OR cannabis[Title/Abstract] OR mephedrone[Title/Abstract] OR GHB[Title/Abstract] OR ketamine[Title/Abstract] OR poppers[Title/Abstract] OR amyl nitrite[Title/Abstract] OR phenethylamines[Title/Abstract] OR DMT[Title/Abstract]) |
| 5 | (#1 OR #2 OR #3 OR #4) |
| 6 | Substance Abuse Detection [MeSH Major Topic] |
| 7 | ("alcohol related disorders/prevention and control"[MeSH Major Topic]) |
| 8 | ("substance related disorders/prevention and control"[MeSH Major Topic]) |
| 9 | "health promotion/methods" [MeSH Major Topic] |
| 10 | Risk Management [MeSH Major Topic] |
| 11 | (Laws OR policy OR policing OR governance OR intervention OR licens* OR education OR service* OR community OR framework) |
| 12 | (medical intervention OR evidence-based OR monitor OR risk reduction OR drug safety testing OR drug checking OR pill testing OR urban design OR environment* OR medical first aid OR multicomponent OR legislat* OR decriminalization OR deterrence OR staff training OR treatment OR prevention OR evaluation OR outcome OR harm reduction OR drug policy OR alcohol policy OR health policy OR treatment OR prevention OR responsible service of alcohol OR lockout OR chillou site* OR peer group*) |
| 13 | #6 OR #7 OR #8 OR #9 OR #10 OR #11 OR #12 |
| 14 | (Nightlife settings OR safer nightlife OR licensed venue* OR recreational setting* or licensed premise* OR entertainment precincts OR licensure OR pub OR club OR patron OR attendee* OR nightclub* OR disco* OR bar OR lounge OR festival* OR rave OR music) |
| 15 | 5 AND 13 AND 14 |
| 16 | Limit 15 to: language: English; publication date: 2010–2021 |

**Embase/ PsycINFO**

| 1 | ("Drug use" or "substance use" or "illicit drug*" or "illegal drug*" or narcotic* or "recreational drug*" or "street drug*" or alcohol or polydrug).mp. |
| --- | --- |
| 2 | ("Designer drug*" or "club drug*" or "legal high*" or stimulant* or hallucinogen*).mp. |
| 3 | (alcohol or beverage* or liquor* or binge* or drunk).ti,ab. |
| 4 | (ecstasy or mdma or mushrooms or psilocybin or lsd or amphetamines or dexamphetamine or methamphetamine or cocaine or cannabis or mephedrone or ghb or ketamine or poppers or amyl nitrite or phenethylamines or dmt).ti,ab. |
| 5 | 1 or 2 or 3 or 4 |
| 6 | substance abuse/co, di, pc, rh, th |
| 7 | drinking behavior/ae, co, dt, ep, pc, th |
| 8 | drug dependence/ |
| 9 | health promotion/ |
| 10 | risk reduction/ or health behaviour/ |
| 11 | (laws or policy or policing or governance or intervention or licens* or education or service* or community or framework).mp. |
| 12 | ("Medical intervention" or evidence-based or monitor or "risk reduction" or "drug safety testing" or "drug checking" or "pill testing" or "urban design" or environment* or "medical" or "first aid" or multicomponent or legislat* or decriminalisation or deterrence or "staff training" or treatment or prevention or evaluation or outcome or "harm reduction" or "drug policy" or "alcohol policy" or "health policy" or treatment or prevention or "responsible service of alcohol" or lockouts or "chillout site*" or "peer group*").mp. |
| 13 | 6 or 7 or 8 or 9 or 10 or 11 or 12 |
| 14 | ("Nightlife settings" or "safer nightlife" or "licensed venue*" or "recreational setting*" or "licensed premise*" or "entertainment precincts" or licensure or pub or club or patron or attendee* or nightclub* or disco* or bar or lounge or festival* or rave or music).mp. |
| 15 | 5 and 13 and 14 |
| 16 | limit 15 to (english language and yr="2010 - 2021") |

**Cochrane Central Register of Controlled Trials**

| 1 | Drug use OR "substance use" O "illicit drug*" OR "illegal drug*" OR narcotic* OR "recreational drug*" OR "street drug*" OR alcohol OR polydrug |
| --- | --- |
| 2 | Designer drug* OR "club drug*" OR "legal high*" OR stimulant* OR hallucinogen* |
| 3 | (Alcohol OR beverage* OR liquor* OR binge* OR drunk):ti,ab |
| 4 | (Ecstasy OR MDMA OR mushrooms OR psilocybin OR LSD OR amphetamines OR dexamphetamine OR methamphetamine OR cocaine OR cannabis OR mephedrone OR GHB OR ketamine OR poppers OR amyl nitrite OR phenethylamines OR DMT):ti,ab |
| 5 | #1 OR #2 OR #3 OR #4 |
| 6 | Substance Abuse Detection/ |
| 7 | Health Promotion/ Methods/ |
| 8 | Alcohol Drinking/ Prevention and Control/ |
| 9 | Substance-related disorder/ prevention and control/ |
| 10 | Risk Management/ |
| 11 | Laws OR policy OR policing OR governance OR intervention OR licens* OR education OR service* OR community OR framework |
| 12 | Medical intervention OR evidence-based OR monitor OR "risk reduction" OR "drug safety  testing" OR "drug checking" OR "pill testing" OR "urban design" OR environment* OR "medical" or "first aid" OR multicomponent OR legislat* OR decriminalisation OR deterrence OR "staff training"  OR treatment OR prevention OR evaluation OR outcome OR "harm reduction" OR "drug policy"  OR "alcohol policy" OR "health policy" OR treatment OR prevention OR "responsible service of  alcohol" OR lockouts OR "peer group*" |
| 13 | #6 OR #7 OR #8 OR #9 OR #10 OR #11 OR #12 |
| 14 | Nightlife settings or "safer nightlife" or "licensed venue*" or "recreational setting*" or "licensed premise*" or "entertainment precincts" or licensure or pub or club or patron or attendee* or nightclub* or disco* or bar or lounge or festival* or rave or music |
| 15 | #5 AND #13 AND #14 |
| 16 | limit 15 to (english language and yr="2010 - 2021") |

**CINAHL/ Web of Science**

| 1 | TX "Drug use" or "substance use" or "illicit drug*" or "illegal drug*" or narcotic* or "recreational drug*" or "street drug*" or alcohol or polydrug |
| --- | --- |
| 2 | TX "Designer drug*" or "club drug*" or "legal high*" or stimulant* or hallucinogen* |
| 3 | TI ( alcohol or beverage* or liquor* or binge* or drunk ) OR AB ( alcohol or beverage* or liquor* or binge* or drunk ) |
| 4 | TI ( ecstasy or mdma or mushrooms or psilocybin or lsd or amphetamines or dexamphetamine or methamphetamine or cocaine or cannabis or mephedrone or ghb or ketamine or poppers or amyl nitrite or phenethylamines or dmt ) OR AB ( ecstasy or mdma or mushrooms or psilocybin or lsd or amphetamines or dexamphetamine or methamphetamine or cocaine or cannabis or mephedrone or ghb or ketamine or poppers or amyl nitrite or phenethylamines or dmt ) |
| 5 | S1 OR S2 OR S3 OR S4 |
| 6 | TX laws or policy or policing or governance or intervention or licens* or education or service* or community or framework |
| 7 | TX (Medical intervention or evidence-based or monitor or "risk reduction" or "drug safety testing" or "drug checking" or "pill testing" or "urban design" or environment* or "medical" or "first aid" or multicomponent or legislat* or decriminalisation or deterrence or "staff training" or treatment or prevention or evaluation or outcome or "harm reduction" or "drug policy" or "alcohol policy" or "health policy" or treatment or prevention or "responsible service of alcohol" or lockouts or "chillout site*" or "peer group*") |
| 8 | S6 OR S7 |
| 9 | "Nightlife settings" or "safer nightlife" or "licensed venue*" or "recreational setting*" or "licensed premise*" or "entertainment precincts" or licensure or pub or club or patron or attendee* or nightclub* or disco* or bar or lounge or festival* or rave or music |
| 10 | S5 AND S8 AND S9 Limiters - Date Published: 20100101-20211231; Language: English |

**ProQuest Social Science database**

(((TIAB("Drug use" OR "substance use" OR ("illicit drug" OR "illicit drugs") OR ("illegal drug" OR "illegal drugs") OR narcotic* OR ("recreational drug" OR "recreational drugs") OR ("street drug" OR "street drugs") OR alcohol OR polydrug) OR TIAB(("designer drug" OR "designer drugs") OR ("club drug" OR "club drugs") OR ("legal high" OR "legal highs") OR stimulant* OR hallucinogen*) OR noft(alcohol OR beverage* OR liquor* OR binge* OR drunk) OR noft(ecstasy OR mdma OR mushrooms OR psilocybin OR lsd OR amphetamines OR dexamphetamine OR methamphetamine OR cocaine OR cannabis OR mephedrone OR ghb OR ketamine OR poppers OR amyl nitrite OR phenethylamines OR dmt)) AND (TIABSU(Laws OR policy OR policing OR governance OR intervention OR licens* OR education OR service* OR community OR framework) OR TIABSU("medical intervention" OR "evidence-based" OR monitor OR "risk reduction" OR "drug safety testing" OR "drug checking" OR "pill testing" OR "urban design" OR environment* OR medical OR "first aid" OR multicomponent OR legislat* OR decriminalization OR deterrence OR "staff training" OR treatment OR prevention OR evaluation OR outcome OR "harm reduction" OR "drug policy" OR "alcohol policy" OR "health policy" OR treatment OR prevention OR "responsible service" OR lockout OR "chillout site*" OR ("peer group" OR "peer groups"))) AND TIABSU("Nightlife settings" OR "safer nightlife" OR ("licensed venue" OR "licensed venues") OR "recreational setting*" OR ("licensed premises") OR "entertainment precincts" OR licensure OR pub OR club OR patron OR attendee* OR nightclub* OR disco* OR bar OR lounge OR festival* OR rave OR music)) NOT TIAB(Animal* OR Mice* OR Mouse OR Cancer* OR Rat* OR pregnancy OR binge-eating OR transplant OR "treatment-resistant" depression OR immunotherapy OR acupuncture OR patholog* OR chemotherap* OR Child*)) AND pd(20100101-20211231)

**Criminal Justice Abstracts**

| 1 | TX "Drug use" or "substance use" or "illicit drug*" or "illegal drug*" or narcotic* or "recreational drug*" or "street drug*" or alcohol or polydrug |
| --- | --- |
| 2 | TX "Designer drug*" or "club drug*" or "legal high*" or stimulant* or hallucinogen* |
| 3 | TI ( alcohol or beverage* or liquor* or binge* or drunk ) OR AB ( alcohol or beverage* or liquor* or binge* or drunk ) |
| 4 | TI ( ecstasy or mdma or mushrooms or psilocybin or lsd or amphetamines or dexamphetamine or methamphetamine or cocaine or cannabis or mephedrone or ghb or ketamine or poppers or amyl nitrite or phenethylamines or dmt ) OR AB ( ecstasy or mdma or mushrooms or psilocybin or lsd or amphetamines or dexamphetamine or methamphetamine or cocaine or cannabis or mephedrone or ghb or ketamine or poppers or amyl nitrite or phenethylamines or dmt ) |
| 5 | S1 OR S2 OR S3 OR S4 |
| 6 | TX laws or policy or policing or governance or intervention or licens* or education or service* or community or framework |
| 7 | TX (Medical intervention or evidence-based or monitor or "risk reduction" or "drug safety testing" or "drug checking" or "pill testing" or "urban design" or environment* or "medical" or "first aid" or multicomponent or legislat* or decriminalisation or deterrence or "staff training" or treatment or prevention or evaluation or outcome or "harm reduction" or "drug policy" or "alcohol policy" or "health policy" or treatment or prevention or "responsible service of alcohol" or lockouts or "chillout site*" or "peer group*") |
| 8 | S6 OR S7 |
| 9 | "Nightlife settings" or "safer nightlife" or "licensed venue*" or "recreational setting*" or "licensed premise*" or "entertainment precincts" or licensure or pub or club or patron or attendee* or nightclub* or disco* or bar or lounge or festival* or rave or music |
| 10 | S5 AND S8 AND S9 Limiters - Date Published: 20100101-20211231; Language: English |
