# Supplementary material for: A systematic review of interventions that impact alcohol and other drug-related harms in licensed entertainment settings and outdoor music festivals
Source: Harm Reduct J. 2024 Feb 21;21:47. doi: 10.1186/s12954-024-00949-4 (PMC10882826; doi:10.1186/s12954-024-00949-4)
Supplement: Supplementary file 4 — Additional file 4: Descriptive statistics for studies included in review (n = 100). [file 12954_2024_949_MOESM4_ESM.docx]

Additional File 4. Descriptive statistics for studies included in review (n=100)

| VARIABLE | | COUNT  N=100 | |
| --- | --- | --- | --- |
| **COUNTRY** | |  | |
| Australia | | 41 | |
| UK | | 19 | |
| US | | 18 | |
| Sweden | | 4 | |
| New Zealand | | 3 | |
| Brazil | | 2 | |
| Canada | | 2 | |
| Norway | | 2 | |
| Colombia | | 1 | |
| Germany | | 1 | |
| Iceland | | 1 | |
| India | | 1 | |
| Mexico | | 1 | |
| Netherlands | | 1 | |
| Portugal | | 1 | |
| Spain | | 1 | |
| Multiple (Australia and Canada) | | 1 | |
|  | |  | |
| **AOD** | |  | |
| Alcohol | | 86 | |
| Illicit Drugs | | 7 | |
| Both | | 7 | |
|  | |  | |
| **SETTING** | |  | |
| Licensed entertainment settings | | 88 | |
| Outdoor music festival | | 11 | |
| Both | | 1 | |
|  | |  | |
| **INTERVENTION** | |  | |
| Laws and Regulations | | 28 | |
| *Alcohol availability* | | 2 | |
| *Lockouts/one-way door policy* | | 5 | |
| *Risk Based Licensing* | | 4 | |
| *Change in trading hours* | | 10 | |
| *Legal purchase age* | | 1 | |
| *Smoking ban* | | 5 | |
| *Regulatory compliance and enforcement* | | 1 | |
| Drug checking/Pill testing | | 2 | |
| Transport intervention | | 6 | |
| Policing strategy | | 6 | |
| Medical services | | 5 | |
| Chill/safe space and roaming support services | | 5 | |
| Staff and venue intervention | | 5 | |
| Patron survey and assessment feedback | | 2 | |

| Multicomponent Interventions | | | | 41 | | |
| --- | --- | --- | --- | --- | --- | --- |
| *Sydney “lockout” Laws* | | | | 8 | | |
| *Queensland “Tackling Alcohol-Fuelled Violence” Policy* | | | | 5 | | |
| *Newcastle Liquor Licensing Restrictions* | | | | 5 | | |
| *United States Multicomponent Interventions* | | | | 6 | | |
| *New Zealand’s Sale and Supply of Alcohol Act 2012* | | | | 2 | | |
| *UK Multicomponent Interventions* | | | | 5 | | |
| *SALUTT and STAD Interventions in Northern Europe* | | | | 5 | | |
| *Other Multicomponent Interventions* | | | | 5 | | |
|  | | | |  | | |
| **STUDY DESIGN** |  | | | | | |
| Quantitative | 85 | | | | | |
| Qualitative | 6 | | | | | |
| Mixed methods | 9 | | | | | |
|  |  | | | | | |
| Interrupted time series | 38 | | | | | |
| Before-and-after | 29 | | | | | |
| Cross-sectional (one group) | 8 | | | | | |
| Randomized controlled trials | 5 | | | | | |
| Non-randomized controlled trials | 2 | | | | | |
| Prospective cohort | 2 | | | | | |
| Cross-sectional (multi group) | 1 | | | | | |
| Case Series | 1 | | | | | |
| Quasi-randomized controlled trials | 1 | | | | | |
| Other | 13 | | | | | |
|  |  | | | | | |
| **SUMMARY OF OUTCOMES** |  | | | | | |
| Health | 40 | | | | | |
| *Positive effect* | 21 | | | | | |
| *Negative effect* | 4 | | | | | |
| *No effect* | 15 | | | | | |
| *Mixed effect* | 2 | | | | | |
| Criminal Justice | 65 | | | | | |
| *Positive effect* | 47 | | | | | |
| *Negative effect* | 10 | | | | | |
| *No effect* | 44 | | | | | |
| *Mixed effect* | 5 | | | | | |
| Behavioural | 20 | | | | | |
| *Positive effect* | 10 | | | | | |
| *Negative effect* | 9 | | | | | |
| *No effect* | 0 | | | | | |
| *Mixed effect* | 1 | | | | | |
|  |  | | | | | |
