# Supplementary material for: A systematic review of interventions that impact alcohol and other drug-related harms in licensed entertainment settings and outdoor music festivals
Source: Harm Reduct J. 2024 Feb 21;21:47. doi: 10.1186/s12954-024-00949-4 (PMC10882826; doi:10.1186/s12954-024-00949-4)
Supplement: Supplementary file 5 — Additional file 5: Summary of studies included in the review on the effectiveness of AOD harm reduction strategies in licensed entertainment settings and outdoor music festivals (2010–2021) (n = 100), grouped by intervention type. [file 12954_2024_949_MOESM5_ESM.docx]

**Additional File 5: Summary of studies included in the review on the effectiveness of AOD harm reduction strategies in licensed entertainment settings and outdoor music festivals (2010-2021) (n=100), grouped by intervention type**

| Rating | Authors, Country | Design | Substance | Setting/Sample | Measures | Method of Analysis | Outcomes |
| --- | --- | --- | --- | --- | --- | --- | --- |
| **Drug Checking/Pill Testing** | | | | | | | |
| W | Measham (2019), UK (1) | Before and after | Illicit drugs | Four-day outdoor music festival in July 2016.  N=230 drug checking service clients | Drug checking service involving brief interventions delivered by qualified health professionals | Descriptive statistics | **Health:**  *Hospital Admissions*  95% reduction in drug-related hospital admissions in 2016 compared to 2015 |
| W | Measham (2021), UK (2) | Before and after | Illicit drugs | Three single day outdoor music festivals in 2017:  1. Two electronic music festivals with predominantly young attendees.  2. One family-oriented festival with a broad range of music and entertainment  N=1,482 drug checking service clients who provided samples for analysis and completed surveys, and N=130 drug checking service clients who completed online follow-up survey post-festival | Drug checking service involving brief interventions delivered by qualified health professionals | Descriptive statistics | **Behavioural:**  *Risky Consumption Practices*  1 in 5 service users whose sample was identified as other than expected handed over further substances of concern for destruction by police; 3 in 10 whose sample was identified as other than expected disposed or intended to dispose further substances of concern.  2 in 5 service users whose sample was identified as expected stated they intended to take a smaller dose of further substances.  For follow-up respondents whose test identified the expected substance but at a higher strength than anticipated, 59.4% (n=19) took a lower dose and 12.5% (n=4) did not take any more of the substance*.* |
| **Policing Strategy** | | | | | | | |
| M | Curtis et al.  (2022), Australia (3) | Before and after | Alcohol | One LEP in Victoria  N=104 patrons received police-imposed bans | 72 h police-imposed bans from 1 of 20 designated areas in urban centres and entertainment precincts | Wilcoxon Signed-Rank Tests.    2010-2017 | **Criminal Justice:**  *Assault, Violence, General Crime, Public Order Offense*  7 years of data: No statistically significant difference in median number of charges for antisocial behaviours pre-post the ban.  4 years of data: Median number of charges 2 yrs. pre vs 2 yrs. post ban – Significant increase post ban in antisocial behaviour generally (*z*= −2.50, *p=*0.01); and for public order offences (*z*= −3.22, *p<*0.01). |
| W | Grigg et al.  (2018), Australia (4) | Cross-sectional – one group | Illicit Drugs | Outdoor music festivals in Victoria or Western Australia  N=1,967  Med age=20 yrs.  59% Male  Subsamples:   1. Participants expecting drug detection dogs at a festival (n=647) 2. Participants who carried drugs in (n=418) 3. Participants who had drugs on their person when seeing a dog (n=189) | The use of specially trained drug detection dogs | Descriptive statistics   - Chi-square tests | **Behavioural:**  *Risky Consumption Practices*  In response to drug detection dogs at an outdoor music festival  Sample 1:  10% took less easily detected drugs and 7% took drugs before entering.  Sample 2:  10% concealed them internally and 1% swallowed them to retrieve them inside.  Sample 3:  10% reported ‘panic’ consuming drugs in response.  The proportion of participants reporting internal concealment of drugs was statistically significantly greater for those expecting drug detection dogs versus those who were not (p=0.001).  The proportion of participants reporting obtaining drugs inside to avoid detection was significantly greater for those expecting dogs to be present (p<0.001). |
| W | Hickey et al.  (2012)  Australia (5) | Cross-sectional – one group | Illicit Drugs | Nightclubs and outdoor music festivals across Australia  N=2,127  60% Male (SD±6)  Med Age=25 yrs. | The use of specially trained drug detection dogs | Descriptive data analysis  2008–2010 | **Criminal Justice Outcomes:**  *Drug Offences*  Among those who did receive a positive notification from a sniffer dog when they had drugs on them during the past 6 months, (13–43%) reported that they had been arrested/cautioned/fined due to this search. No significant differences noted across the three samples.  **Behavioural Outcomes:**  *Risky Consumption Practices*  Of participants who had sighted a sniffer dog while in possession, 36% consumed the drugs straight away. |
| S | Malins  (2019), Australia (6) | Semi-structured in-depth interviews | Illicit Drugs | Outdoor music festivals, licensed entertainment venues, and public transport spaces in NSW, Victoria and Qld  N=22 participants who had been searched by drug detection dogs.  Sub-samples  n=4 for 18–24 yrs. old, n=11 for 25–29; n=4 for 30–39; n=2 for 40–49 and n=1 for 60+. | The use of specially trained drug detection dogs | Deleuzo-Guattarian assemblage-thinking and thematic analysis  February 2016–August 2018 | **Behavioural Outcomes:**  *Risky consumption practices*  People made several adaptions toevade detection by drug dogs, all of them considered comparatively risky consumption or purchase practices. These included internal concealment (arranged before the gates), pre-loading drugs (consuming them prior to entry), choosing to purchase drugs inside the venue, and panic consumption of all drugs in possession upon seeing drug dogs at the gates. |
| M | Rowe et al.  (2012), Australia (7) | Before and after | Alcohol | Licensed entertainment premises in NSW  N=21 area commands non-metropolitan NSW | Venues received one of three levels of police response:  1. Letters  2. Incident reports, covert audits and  3. Feedback meetings. | Descriptive statistics  December 2002 to July 2003 | **Criminal Justice Outcomes:**  *Other offences – Police Incidents* (includes incidents of violence, disorder, and motor vehicle crashes). No effect except for a nonsignificant reduction in police incidents (1.24 per premises at baseline to 1.11 at follow-up). At high-risk venues there were significant reductions in police incidents (from 7.08 to 5.65 patrons (p=0.03)) and a significant reduction in the rate of intoxicated patrons involved in such incidents, from 5.50 to 4.40 (p=0.05). |
| S | Taylor et al.  (2021),  Australia (8) | ITS | Alcohol | LEPs in Brisbane  Across all precincts (combined) min and max events were:  N=1137–4573 police banning orders  N=242–807 serious assaults  N=134–489 common assaults  N=1669–7762 good order offences | 1. Minimum 10-day police-issued patron bans (bans patron from entering or remaining in the designated licensed premises or NEP for the ban duration)  2. Mandatory ID scanners for all licensed venues open after 12am, to be used from 10pm | ARIMA models  1st October 2014–31st June 2018 | **Criminal Justice:**  *Assault:*  Number of police-issued patron bans did not significantly predict changes in serious assaults, common assaults or good order offences in weekend following ban in Brisbane CBD, Fortitude Valley and Surfer’s Paradise.  *Serious assaults:*  Brisbane CBD/ Fortitude Valley: No seasonality found; ARIMA (0,0,1) Q=44.16, *p*=0.30.  Surfer’s Paradise: No seasonality found; ARIMA (0,0,1) Q=44.16, *p*=0.30  *Common assaults:*  Brisbane CBD/ Fortitude Valley: Seasonality found; ARIMA (0,0,0), Q=41.21, *p*=0.42  Surfer’s Paradise: No seasonality found; ARIMA (0,0,0), Q=26.01, *p=*0.96  *Good order offences:*  Brisbane CBD/Fortitude Valley: No seasonality found; ARIMA (0,1,1), Q=38.47, *p=*0.54  Surfer’s Paradise: No seasonality found: ARIMA (1,0,0), Q=30.79, *p=*0.85 |
| **Medical Service** | | | | | | | |
| W | Archer et al. (2012), UK (9) | Prospective Cohort | Alcohol and illicit drugs | Outdoor music festival in South UK  N=28 (26%) patients were seen with acute AOD toxicity. | Physician-led medical facility comparable to a small ED | Descriptive statistics and Mann–Whitney U tests.  Study does not specify year range | **Health:**  *Ambulance attendances*  There were fewer patients transferred to hospital from the festival due to the availability of medical personnel onsite. Specifically, n=16 were transferred under the ambulance referral criteria, and n=4 transfers were required in the context of onsite medical personnel. |
| W | Dutch et al. (2012), Australia (10) | Cross-sectional - one group | Illicit drugs | 24 outdoor music festivals in Victoria  N=61 patients with GHB intoxication | St John’s Ambulance medical assistance teams | Descriptive statistics and chi-square tests  January 2010–May 2011 | **Health:**  *Hospitalisations and ambulance attendances*  Medical teams were able to avoid hospital transfer via ambulance in 65% of patients. |
| W | Friedman et al. (2019), USA (11) | Cross-sectional - one group | Alcohol and illicit drugs | Outdoor music festival in New York, USA over 4 years  N=54 patients received emergency medical care over 4 years. | Emergency care delivered by a collegiate-based, volunteer first-response service in coordination with a contracted, private ambulance service | Descriptive statistics  2014–2017 | **Health:**  *Ambulance attendances*  May have reduced strain on local medical resources. Mutual aid from the county emergency medical services was requested for only 22.2% of cases. |
| W | Lund et al. (2015), Canada (12) | Cross-sectional - one group | Alcohol and illicit drugs | Outdoor music festival  N=70 patient encounters | Higher-level care medical service (i.e., clinical monitoring, diagnostic decision making, and critical care experience) | Descriptive statistics  Study does not specify year range | **Health:**  *Ambulance attendances*  The ambulance transfer rate with first aid only would have been 1.98; ambulance transfer rate with higher-level care model was 0.52. Presence of on-site higher-level care team had significant positive effect on avoiding ambulance transfers. |
| W | Wood et al. (2010), UK (13) | Cross-sectional - one group | Alcohol and illicit drugs | Outdoor music festival and an outdoor after party event  N=227 patient presentations | St. John’s Ambulance medical services incl. critical medical intervention tents (advanced treatment centres) | Descriptive statistics  Study does not specify year range | **Health:**  *Hospitalisations*  There was a significantly lower transfer rate of individuals with drug or drug/alcohol toxicity from the main outdoor day event, where there was physician level cover, compared to the outdoor after party event where there was not (10.5 vs. 55.5%, p=.002). |
| **Chill/Safe spaces and Roaming Support Services** | | | | | | | |
| W | Carvalho et al. (2014), Portugal (14) | Before and after | Illicit drugs | Outdoor music festival in Idanha-a-Nova  N=176 Kosmicare clients | Kosmicare project: Crisis intervention related to use of psychoactive substances, harm reduction and risk minimisation strategies (information and outreach, chill-out, drug use paraphernalia, testing) and a care space for people undergoing difficult psychedelic and emotional experiences. | Descriptive statistics, SWOT analysis and content analysis | **Health:**  *Mental Health*  A pre-post mental state evaluation showed a statistically significant decrease in mental state exam symptoms, indicating successful crisis resolution. Qualitative analyses shows that 76% of crisis episodes obtained resolution. |
| W | Doran et al. (2021), Australia (15) | Before and after | Alcohol | LEPs in New South Wales - Town Hall, Kings Cross, and Darling Harbour  N=66,455 people supported by TKSS ambassadors | Take Kare Safe Space Program (TKSS)  1. Street patrols offering assistance  2. Static safe spaces for rest and recovery | Descriptive analysis | Of all incidents, serious risk of harm was averted from the following in 20% of incidents:    **Health:**  *Road Traffic Accidents:* 12%  **Criminal Justice:**  *Assault:* 17%  *Sexual Assault:* 8%  *Theft:* 40% |
| W | Garius et al. (2020), UK (16) | Before and after | Alcohol | LEPs and an outdoor music festival in two unnamed cities | Drinkaware Crew: Patrols of two people trained to provide assistance for intoxicated patrons. | Standard mean difference tests  2015–2017 | **Criminal Justice:**  *Assault/Sexual Assault*  Overall, analysis suggests an inconclusive effect of the initiative in the test venue in City A, and a negative effect at the test venues in City B. |
| S | Taylor et al. (2020), Australia (17) | ITS | Alcohol | LEP in Cairns, Queensland.  N=190 licensed venues | Street service care, incl.:  1. Street patrols offering assistance  2. Static safe spaces for rest and recovery  3. First aid | ARIMA models  1 January 2009 to 31 December 2017 | **Health:**  *Hospitalisations*  No significant impact during HAH  *Ambulance attendances*  No significant impact during HAH  **Criminal Justice:**  *Assault*  Significant reduction with a 1 month lagged impact during HAH (*B*=−1.66, p=0.02, 95% CI −3.02, −0.30) |
| W | Ward et al. (2018), Australia (18) | Mixed methods -Observations and street intercept surveys with venue patrons | Alcohol | LEP in Melbourne, Australia  n=16 informant interviews | A volunteer-staffed, mobile van, and secured rest and recovery space, incl. first aid. | An intrinsic case-study approach, including:   - Document reviews - Qualitative interviews - Observation - Secondary data analysis   2010–2015 | **Health:**  *Hospitalisations*  No significant impact on the proportion of young people presenting for alcohol-related harms  **Criminal Justice:**  *Assault*  No significant reduction in the proportion of police reports during HAH for alcohol-related incidents  *Violence*  Presence of security sometimes inadvertently resulted in escalation |

| **Transport Intervention** | | | | | | | |
| --- | --- | --- | --- | --- | --- | --- | --- |
| W | Curtis et al. (2019), Australia (19) | Before and after | Alcohol | Melbourne City-Wide Public Transportation | The introduction of 24hr public transport | Descriptive statistics:   - T-tests - Mann-Whitney *U­*-tests   2015–2016 | 2015-2016 comparison  **Health:**  *Road traffic accidents*  No reduction in average number of crashes on Friday/Saturday nights. Significant increase in crashes Sunday morning between 1-2am, p=.002 (this increase is likely due to time displacement)  *Ambulance attendances*  No significant change in the mean number of ambulance attendances for each HAH.  **Criminal Justice:**  *Assaults*  Slight increase in police-recorded assaults during HAH during three quarters of 2016 (slight decrease in July-September) |
| W | Curtis et al. (2019), Australia (20) | Before and after and cross-sectional | Alcohol and illicit drug | Nightclubs and Public Transport Hub in vicinity of nightclubs in Melbourne, Victoria  N=505 venue observation checklists  N=207 survey participants | The introduction of 24hr public transport | Pre-post venue observations and cross-sectional street intercept patron surveys | **Health:**  *General Injuries*  No reported change in additional alcohol-related injuries since the introduction of 24-h public transport.  **Behavioural:**  *Aggression*  3% reported experiencing more aggression and unwanted sexual attention.  *Using Public Transport*  56% reported increasing their train use. |
| S | Jackson et al. (2011), USA (21) | Before and after | Alcohol | City-wide licensed premises in Washington D.C. | Gradually extended metro system end of service time, from:   - Midnight to 1 am - 1 am to 2 am - 2 am to 3 am | Difference in difference in difference analysis  1999–2007 (intervention occurred staggered between November 5th 1999 and July 4th 2003) | **Criminal Justice:**  *Driving Offences*  Neighbourhoods with ≥ 1bar and a nearby metro station experienced ~ 14% reduction in the probability of a DUI arrest, following the expansion of the service by 3 hrs.  *Liquor Offences*  Neighbourhoods with ≥ 1bar and a nearby metro station experienced an increase in number of alcohol-related arrests, following the expansion of the service by 3 hours. |
| W | Kazbour et al. (2010), USA (22) | (Repeated) Cross-sectional study | Alcohol | One licensed bar in Florida  Sample size not reported | Measures targeting designated drivers (and passengers).  Stages:  1. Free soft drinks  2. Free pizza  3. Free pizza and gas | Descriptive statistics  Thursday and Friday nights from 12:00 a.m. until 2:00 a.m. across 8 weeks for a total of 16 sessions in 2000. | **Criminal Justice:**  *Drink Driving*  Post-intervention, proportion of patrons riding with/acting as a designated driver increased.  (0.5% pre - 12% post, with high of 24%) |
| S | Miller T et al. (2020), USA (23) | Other - ITS and cross-sectional surveys | Alcohol | LEP in Columbus, Ohio  N=19,649 coupon redeemers | Intervention administered in partnership with a national ridesharing service:  1. Thursdays at 2pm, 2,000 two-trip ride coupon codes with a $30 maximum value could be downloaded by Columbus area residents  2. Codes were valid for rides to/from either of the city’s two main bar and restaurant zones on Thursday, Friday, or Saturday of that same week, expiring on Sunday. | ARIMA models  A 17-week intervention period in 2017 (September 7 – December 31).  ARIMA model is from January 2016–December 2018 | **Criminal Justice:**  *Driving Offenses*  While there was a small change in crash incidence, the ARIMA models did not detect a change in:   1. Number of crashes when operating vehicle while impaired (OVI) 2. Percentage of crashes that were OVI 3. Ratio of night-time single vehicle crashes versus multiple vehicle daytime crashes.   **Health:**  *Road Traffic Accidents*  Coupon users avoided an estimated 3.2 impaired driving crashes or 1 per 4,309 trips (78.9% of alcohol-involved crashes were alcohol attributable). The associated savings were 1.11 years of healthy life.  **Behavioural:**  *Using public transport/ridesharing*  70.8% said that coupon availability decreased the likelihood that they would have driven after drinking that day. |
| W | Rivara et al. (2012), USA (24) | Cross-sectional multigroup | Alcohol | Licensed venues (bars) in Seattle, Washington. | Social marketing campaign, “Last Call”. Key features included:  1. Use of taxi stands to promote taxi use.  2. Point-of-sale information to patrons at partner bars.  3. Mass media campaign to support the designated driver/safe ride home message. | Descriptive statistics and multivariate analysis  2001–2008 | **Health:**  *Road traffic accidents*  Reduction in single vehicle night-time collisions 21–34-year-old drivers. The rate per 100,000 of single vehicle night-time crashes (a proxy for alcohol involved crashes) among 21–34-year-old drivers decreased substantially in Seattle over the last 2 yrs., while climbing in Portland and staying level in Spokane. |
| **Laws and Regulations** | | | | | | | |
| S | Bassols et al. (2018), Spain (25) | ITS | Alcohol | Licensed venues (bars) across Spain  N=870,061 observations | Reduction in trading hours - from 6am to 2-3:30am | Descriptive statistics, differences-in-differences framework, linear regressions  1990–2011 | **Health:**  *Hospitalisations*  Significant reduction (16.5%) in alcohol-related hospitalisations during weekends for men.  *OHS/Workplace Injuries*  Decrease in the number of working accidents in 2.62 per 100,000 employed individuals, and  9% reduction in workplace accidents (15% for women; 7% for men). |
| S | Bernat et al. (2013), USA (26) | ITS | Alcohol | Bars and restaurants in NY and California  N=312 monthly observations | 100% smoke-free restaurant and bar laws | ITS models (i.e., Box-Jenkins ARIMA models)  1982–2008 | **Health:**  *Road Traffic Accidents and Deaths*  No effect on rates of alcohol-related car crashes or car crash fatalities |
| S | Briggs et al. (2014), USA (27) | ITS | Alcohol | All bars and taverns in Cedar Falls and Waterloo, Iowa  N=2,557 offences on 2,188 residential city blocks; N=3,583 calls reporting assaults | 100% smoke-free restaurant and bar laws | Multivariate logistic regression models  1st January–15th November 2009 | **Criminal Justice:**  *Assaults*  Before the law took effect, the presence of at least one bar on a block or adjoining block increased the expected number of assaults by 110%. Following the implementation of the measure, this increased the expected number of assaults by 73.2%. Overall, the law diminished the strength of the relationship between bar presence and assaults*.* |
| W | Brown et al. (2011), UK (28) | Before and after | Alcohol | LEP in Hartlepool, England  N=325 survey respondents | Local variation in licensing hours to allow for staggered closing times | Descriptive statistics  2005 and November 2009 | **Health:**  *Ambulance attendances*  Ambulance services reported an increase in the number of incidents in the early hours of the morning.  **Criminal Justice:**  *Assaults and public order offences*  Violence against the person decreased by 14%, criminal damage decreased by 15%, and antisocial behaviour increased by 4% in the town centre between 8pm and 4.59am. |
| S | Burgason et al. (2017), USA (29) | Before and after | Alcohol | Late night bars and nightclubs in Little Rock, Arkansas | 1. Minimum of two law enforcement officers at exterior premises from 12am until closing; same number of security personnel inside the venue.  2. Adequate lighting and video surveillance and signage prohibiting loitering.  3. Penalties for non-compliance with mandates.  4. Penalties for continued violent offending.  5. Police power to intervene if law violations continue incl. suspension or revocation of license to operate.  6. Mandated closing time: 3am and 5am. | RTMDx (risk terrain modelling diagnostics software); year-long post-ordinance model.   - Pre-ordinance RTM from November 2013–October 2014. - Post-ordinance November 2014–October 2015, for comparison. | **Criminal Justice:**  *Violence*  23% reduction in reported violent crime from 2014 to 2015. The intervention strategies were effective to alter the guardianship at these establishments and reduced the relative risk of violence occurring in close proximity. |
| S | Curtis et al. (2019), Australia (30) | ITS | Alcohol | All licensed venues in Victoria and Queensland  Victoria: N=507,870 ED presentations during HAH; 639,635 during LAH  Queensland: N=164,411 ED presentations during HAH; 487,144 during LAH | RBL | ITS analysis, incl.:   - Prais regression models, and Breusch-Godfrey tests to assess residual autocorrelation   Victoria: 2000–2015  Queensland: 2007–2015 | **Health:**  *Hospitalisations*  No overall reduction in incidence of ED injury presentations during HAH in Queensland (β=0.003; 95% CI: −0.010, 0.003, p=0.318) or Victoria (β=−0.010; 95% CI: -0.021, 0.001, p=0.087). Post-hoc subgroup analyses showed reductions in ED injury presentations among men aged 20–39 yrs in Victoria (β=−0.026; 95% CI: -0.012, −0.040, p-0.0003) but not Queensland. |
| S | de Andrade et al. (2016), Australia (31) | ITS | Alcohol | LEP in Surfers Paradise  N=974 crime incidents at 106 point-specific addresses and 22 street-specific addresses | 3am lockout | ITS analyses   - Prais regression models   July 2003–June 2006 | **Criminal Justice:**  *General Crime*  No significant change in natural logged weekend counts of crime pre-post intervention.  *Violence*  No significant change in natural logged weekend counts of incidents of violence pre-post intervention.  **Health:**  No significant change in natural logged weekend counts of ambulance attendances for head and neck injuries, and severe intoxication pre-post intervention. |
| S | de Goeij et al. (2015), Netherlands (32) | ITS | Alcohol | LEPs in Amsterdam  2006–2009: N=1,043 ambulance attendances  2009–2011: N=837 ambulance attendances | Trading hour extensions (by 1 hour, or 2 hours for daytime venues on weeknights) | A segmented time–series design:   - Five nested Poisson regression models   2006–2009  2009–2011 | **Health:**  *Ambulance attendances*  A 1-hour extension of alcohol outlet closing times in some of Amsterdam’s nightlife areas was associated with 34% more alcohol-related injuries. |
| M | Forsyth et al. (2012), UK (33) | Other – Observational fieldwork and patron interviews | Alcohol and Illicit Drugs | Eight nightclubs in Glasgow  N=32 nightclub patrons | Smoking ban in all enclosed spaces, incl. licensed premises | Descriptive analysis and unspecified analysis of qualitative interviews  24^th^ February 2006 – 27^th^ May 2006 | **Criminal Justice:**  *Violence*  Violent incidents declined from 22-12, although the authors noted this may be due to weather, examination time or chance. Observations of violence rated “severe” increased post ban, possibly due to strain on security staff.  *Sexual Assault*  Patron interviews suggested that the ban may have inadvertently created opportunities for drink spikers (and thus sexual assault), because smokers often left their drinks unattended when they went outside to smoke on the streets (a by-law in Glasgow prohibits the consumption of alcohol in public places).  **Behavioural:**  *Risky Consumption Practices*  Some patrons may finish their alcoholic drinks more rapidly to prevent drink spiking or theft while they are outside smoking.  *Overcrowding*  Overcrowding decreased, seemingly due to the drop in patron attendances. |
| S | Green et al. (2014), UK (34) | Before and after | Alcohol | LEPs in England and Wales | Trade hour restrictions – Licensed venues permitted to trade later, until 5 am (previously 11pm) | A flexible difference in difference model  2002–2008 | **Health**  *Road Traffic Accidents*  Decline in accidents in intervention site, larger decline on Friday and Saturday nights, concentrated in younger drivers. |
| M | Gruenewald et al. (2015), NZ (35) | Before and after | Alcohol | All licensed entertainment venues and alcohol outlets in New Zealand  N=16,240 survey respondents in total | Lower legal purchase age of alcohol from 20 yrs. to 18 yrs. | Logistic regression  Censored Tobit regression analyses  1995, 2000, 2004 | **Behavioural:**  *Risky consumption practices*   - 3-fold increase in rates of problems per drinking occasion at pubs / nightclubs, across all drinking quantities.     Significantly associations with:   - more frequent drinking at pubs/nightclubs among the newly of-age 18–19-year-olds (β=15.26, *p=*0.009). - moderated drinking quantities at pubs/nightclubs (β=–0.94, *p=*0.034). - problems related to a change in drinking contexts (G2 ≥ 27.45, P ≤ 0.002), specifically when drinking in pubs/nightclubs (β=0.09, P< 0.001) for 16-19 yrs. old. |
| S | Humphreys et al. (2013), UK (36) | ITS | Alcohol | City of Manchester | Extended trading hours (up to 24 hrs (from 11 pm)): on-premises | ARIMA models  1st February 2004–31st December 2007 | **Criminal Justice:**  *Assault*  No evidence of effect on overall volume of violence. There was a temporal shift of weekend violence into later parts of the night (initial increase of 27.5% between 3:00am-6:00am (v=0.2433, 95% CI=0.06, 0.42); increased to 36% by the end of the study period (d=20.897, 95% CI=21.02, 20.77)). |
| M | Humphreys et al. (2014), UK (37) | Before and after | Alcohol | Premises clusters within City of Manchester (not LES) | Extended trading hours (up to 24 hrs (from 11 pm)): on-premises | Spatial auto-repressive regression models  Ordinary Least Squares Linear Regression Models  2004–2008 | **Criminal Justice:**  *Assault*  No significant change in rates of violence. 1.5% increase in violence in the first year post-implementation (0.3 crimes per 1000 h, per km2), followed by a 12% reduction on average baseline rates of violence (−2.5 crimes per 1000 h, per km2) in the second year post-implementation. |
| S | Khurana et al. (2022), India (38) | Before and after | Alcohol | Bars that sell hard liquor in Kerala (treatment), and Karnataka and Tamil Nadu (controls). | Banning local bars from selling hard liquor, and only allowing bars in five-star hotels to sell it. | Difference-in-differences estimation  2010–2015 and study data extended 2016-2018 | **Criminal Justice:**  *Violence, Sexual Assault, and related offenses.*  No significant effect on verbal insults.  Significant reduction (22%) in sexual assault cases reported by women post-closure of hard liquor bars.  Non-significant increase in rapes, possibly due to increased reporting. |
| M | Kirby et al. (2011), UK (39) | Other – Mixed methods incl. semi-structured interviews NTE stakeholders, and police data. | Alcohol | Interviews were conducted with Preston (England) NTE stakeholders:  N=3 police  N=2 licensees  N=5 paramedics  N=1 nurse | The licensing act (2003):  1. Removing trading hour constraints for all licensed premises  2. Staggering closing times | Thematic Analysis  Interviews:2009  Crime Data: 2000–2009 | **Criminal Justice:**  *General Crime*  Alcohol-related crimes declined steeply in 2005 (the year the Act was introduced), staying at much lower levels than before the Act e.g., rates peaked at 2004 (2113), then 2007 (1189), 2008 (1219) and 2009 (1046) respectively (60% decrease). Since the introduction of the Act a larger proportion of those offences are occurring after 3am. Paramedics noted that busy periods for alcohol-related injuries used to be between 11pm and midnight and were now more staggered (11pm-6am). |
| S | Klein et al. (2013), USA (40) | ITS | Alcohol | On-premises alcohol-licensed venues in St Paul, Minnesota.  N=23,978 serious crimes and n=49,560 less serious crimes | Clean indoor air (CIA) policy. | ITS analysis  2003–2017 | **Criminal Justice:**  *Assault, Sexual assault, General Crime, Public order offence, Liquor offence*  No significant change in weekly serious crime. Less serious crime did not change significantly in either the initial effects (*p*=0.20) or the rate of change over time (*p*=0.67). |
| S | Mazerolle et al. (2012), Australia (41) | ITS | Alcohol | QLD LEPS - Fortitude Valley and Airlie Beach  N=140 licensed premises in Fortitude Valley  N=101 licensed premises in Airlie Beach | Liquor amendment act 2006 – 3 am lockout legislation | Generalized linear models.  January 1996 to August 2008. | **Criminal Justice:**  *Assault*  Airlie Beach: Significant reduction in mean number of violent incidents inside licensed venues from 0.55/month to 0.22 month (*p*=0.06; considered significant).  Fortitude Valley: Significant reduction in mean number of violent incidents inside licensed venues from 2.8/month to 1.3/month (*<*0.001). No evidence of impact on violence on streets and footpaths outside licensed premises, where more than 80% of entertainment district violence occurred. |
| S | Miller et al. (2012), Australia (42) | ITS | Alcohol | Licensed entertainment venues in Ballarat (lockout policy) and Geelong (no lockout policy), Victoria  N=337 cases for Ballarat and n=766 cases for Geelong | 3am lockout of licensed venues | ITS analysis   - Durbin-Watson statistic - Ljung-Box statistic   1 July 1999–31 July 2009 | **Health:**  *Hospitalisations*  A small reduction in alcohol-related assaults and intoxication rates within Ballarat occurred before and after the introduction of the lockout (R^2^=0.25, p<0.05). However, after this decline these rates steadily increased (R^2^=0.74, p<0.05), surpassing Geelong by 2005. No discernible long-term impact on alcohol-related ED attendances in Ballarat. |
| S | Miller P. et al (2020), Australia and Canada (43) | Qualitative study with semi-structured key informant interviews | Alcohol | LEPs in:   - Australia - Victoria, Queensland, ACT - Canada – Ontario   N=28 key informants (4 govt policy makers, 4 liquor licensing representatives, 4 local council members, 8 police officers, 6 licensees, 1 academic,  and 1 community advocate) | RBL:  Liquor licencing conditions, incl. fees in some jurisdictions, are based on outlet characteristics judged to be indicators of risk for violence and social disorder. | Thematic Analysis  In-depth interview between October 2016 and April 2018 | **Health:**  *Other*  RBL was generally supported by informants, however, many did not perceive that it had been successful in reducing alcohol-related harm. |
| S | Moore et al. (2012), UK (44) | Randomised controlled trial | Alcohol | LEPs in five Welsh towns:  One large cosmopolitan city that attracts drinkers from across the UK,  A large city with a traditional NTE,  Three smaller towns with well-defined NTE areas | RBL:  1. Action plans to change operating procedures e.g., reducing capacity, changing how security staff are deployed, checking patrons’ age at the door.  2. Improved staff training  3. Covering aspects of the internal and the external environment e.g., addressing poor visibility. | Andersen-Gill model  Standard multilevel mixed-effects linear regression (under powered).  March 2008–February 2009 | **Criminal Justice:**  *Assault*  The introduction of RBL led to no significant change in the number of police recorded assaults. |
| S | Nepal et al.  (2019), Australia (45) | ITS | Alcohol | Licensed premises in Queensland | RBL:  1. Licensees pay an annual base fee plus a risk loading that reflects trading hours and compliance history.  2. Licensing authorities assess the risk that outlets pose to the community, and consider the type of outlet, location, occupancy, activities, and trading hours.  3. Applicants are evaluated on their experience, training, and past conduct, incl. any record of liquor licence infractions. | Breusch–Godfrey test  Pre-intervention period of 2004–2008  Post-intervention period 2009–2014 | **Criminal Justice:**  *Assault*  RBL was not associated with any significant change in the incidence of assault attributed to drinking in licensed premises (β=-8.21; 95% CI: -18.4, 2.01). |
| W | Palk et al. (2010), Australia (46) | Before and after | Alcohol | Licensed entertainment premises  N=478 incidents recorded in/around n=121 licensed premises | Introduction of a lockout policy:  1.Patrons prevented from entering late night liquor trading establishments 3 am - 5 am  2. Patrons inside the licensed premise may remain inside and continue to consume alcohol until 5 am closing | Descriptive statistics   - Chi-square analyses   29 March 2004–2 May 2004 | **Criminal Justice:**  *Assault, sexual assault, driving offenses, general crime, public order offense, liquor offenses*  No statistically significant changes evident for offences against the person, property, stealing or traffic offences.  Sexual offences requiring police attendance dropped by 33.7%  Significantly fewer street disturbances (12.3%), χ2(1, N=321)=5.39; OR=1.75, *p*=.02).  Alcohol-related disturbances were reduced by 6.2%” χ2=15.98, OR=1.2, *p*<0.0001. |
| M | Palk et al. (2012), Australia (47) | Before and after | Alcohol | Licensed entertainment premises  Gold Coast and Brisbane City/Fortitude Valley  Gold Coast: N=12,801 incidents  Brisbane/ Fortitude Valley: N=9,117 incidents | Introduction of the lockout policy:  1. Patrons prevented from entering late night liquor trading establishments from 3am-5am  Patrons inside the licensed premise may remain inside and continue to consume alcohol until 5 a.m. closing | Descriptive statistics:  Chi-squared test  Thematic analysis  February 2004-June 2005 | **Criminal Justice:**  *Driving offenses; sexual assault and related offenses, general crime, public order offense, personal trauma offenses*  Gold Coast  Significantly fewer traffic offences (12.9%) between 3am-6am, χ2 (1, N=157)=4.19, p=.03, OR=0.41.  Sexual offences reduced significantly by 33.7%, χ2 (1, N=80)=8.49, p=.004, OR=4.4.  Reductions (not statistically significant) evident for offences against the person (10.8%), property (0.8%), and stealing (1.7%).  Fortitude Valley  Street disturbances significantly reduced by 16%, χ2 (1, N=130)=3.20, p=.05, OR 0.52.  Alcohol-related disturbances/ disputes significantly decreased (29.0%), χ2 (1, N=57)=4.31, p=.03, OR=0.30, for 12am-3am, but increased significantly (33.8%) 3am- 6am.  Personal trauma offences significantly decreased by 14.6%, χ2 (1, N=85)=4.32, p=.03, OR=0.33, during all other time periods.  No evidence of displacement of alcohol-related offences to non-lockout time periods. Police reported no corresponding overall decreases in offending rates during the current study period.  **Behavioural:**  *Overcrowding*  Lockout possibly created two rush hours that led to overcrowding at entry points to licensed premises and long waiting lines for taxis. |
| M | Ragnarsdóttir et al. (2011), Iceland (48) | Before and after | Alcohol | LEP in Reykjavik’s City Centre  2 studies:  *1^st^ study involved statistics obtained from police reports and the emergency ward of Landspitalinn University Hospital in 2000.  *2^nd^ study involved stakeholder interviews in 2008 | 24h trading hours in Reykjavik’s City Centre | Descriptive statistics  1999–2000 | **Criminal Justice:**  *Assault*  Rise in harmful events in the city centre during the studied time period. In the latter study, the number of incidents of violent offences reported by police increased from 1999–2000.  **Health:**  *Hospitalisations*  ED admissions increased between the two studies. ED workload greatest around the closing time of the bars /restaurants (3am–5am). In the latter study, half of the admissions to ED came from the city centre, with most common reason for presentation being AOD harm. The number of admissions peaked at 4.30am. |
| M | Randerson et al. (2018), NZ (49) | Before and after | Alcohol | Not specified | Sale and Supply of Alcohol Act 2012 (SSAA), included:  1. Trading hour limits of 8am-4am for on-licenses, and 7am-11pm for off-licenses, and a provision enabling territorial authorities to develop a local alcohol policy through public consultations.  2. New RBL fees  3. Small changes to licensing procedure.  4. Requirement to consider the impact of alcohol outlets on amenity and good order near location.  5. Make one-way door policies legally enforceable. | A mixed methods study incorporating key informant interviews (thematic analysis) and administrative data  2013–2015 | **Criminal Justice:**  *Driving Offenses*  24% reduction in apprehension of drivers with a BAC of 0.008, and 35% drivers reported drinking less before driving after the law change.  Drink driving officers also reported a slight improvement in compliance after the law change, finding lower breath alcohol levels in general and more people deciding not to drink at all before driving**.** |
| S | Rossow et al. (2012), Norway (50) | ITS | Alcohol | Licensed premises (pubs, bars, and nightclubs)  18 Norwegian Cities   - 10 with restricted closing hours - 3 with extended closing hours - 5 with extensions then restrictions. | Trading hour restrictions or extensions, for on-premise sales, typically less than 2 hours. | Pooled cross-sectional time-series   - ARIMA - Box–Ljung Q statistics   Q1 2000–Q3 2010 | **Criminal Justice:**  *Assaults*  Each 1-hour extension of closing hours was associated with a statistically significant increase of 4.8 assaults (95% CI 2.60, 6.99) per 100 000 inhabitants per quarter (i.e., an increase of about 16%). |
| S | Taylor et al. (2019), Australia (51) | ITS | Alcohol | Designated safe night precincts (SNPs) and other local govt areas (LGAs) in Queensland | Liquor sales restrictions - state-side restriction on serving rapid intoxication liquor after 00:00 | ARIMA analysis  January 2009–July 2018 | **Criminal Justice:**  *Assaults*  Monthly police-recorded serious assaults did not significantly change within SNPs or LGAs following the introduction of liquor restrictions. Overall, the restrictions did not result in a clear, unique reduction in serious assault trends |
| M | Tesch et al. (2018), Germany (52) | Other - “A quasi-experimental design with data from 13 Bavarian towns with and without restrictive opening hours and a negative binomial panel model” | Alcohol | LEPs/13 Bavarian bars | Extended and restricted trading hours  Each municipal parliament is free to choose exact opening hours including the option of liberalised opening hours (closing at 5am-6am to clean venues). Consequently, opening hours vary 1am-2am weekdays, and 3am-4am on weekends across the towns that decided to restrict them. | Descriptive statistics, and Poisson–Gamma mixture distribution  2002–2013 | **Criminal Justice:**  *Violence*  Extended trading hours reduces nighttime violent incidents by about 21% (1 – 0.809) if other variables are held constant.  Restricted opening hours led to an increase in night-time violence in towns with higher levels of daytime violence. After controlling for daily violence and an interaction between policy regime and level of violence, restricted opening hours are only beneficial within settings that experience low levels of daily violence. |
| M | Tomedi et al. (2018), USA (53) | Before and after | Alcohol | Licensed entertainment settings in New Mexico | Enhanced enforcement on binge drinking intensity among adults drinking in licensed on-premises locations | Descriptive statistics  Pre-intervention 2004–2005  Post-intervention 2007–2008 | **Behavioural:**  *Risky Consumption Practices*  The proportion of binge drinkers in licensed locations who consumed 8+ drinks on a binge drinking occasion decreased from 42.1% in 2004–2005 to 22.6% in 2007–2008 (adjusted OR=0.4; 95% CI=0.2–0.9), while the proportion in unlicensed locations was unchanged. |
| S | Young-Wolff et al. (2013), USA (54) | Prospective cohort | Alcohol | Bars and restaurants in the District of Columbia  Wave I  Wave II Delaware, New York, Connecticut, Maine, Massachusetts, Rhode Island, Vermont, Washington | Smoke-free policies in licensed indoor public venues (including bars and restaurants) | Logistic regression  “Waves I (2001–2002) and II (2004–2005) predicted changes in DSM-IV AUD status (remission, onset, recurrence) in current drinkers at Wave I (n=19,763) and participants who drank in public ≥once per month (n=5913)” | **Health:**  *Other – Alcohol Use Disorder*  For past-year drinkers  The legislation was associated with a significantly greater likelihood (*p*≤0.05) of AUD remission for all participants.  Smokefree legislation was protective against first AUD onsets (p≤0.05) for women, and 40–49 year olds.  Among smokers, the legislation was associated with less risk of AUD onset, and greater risk of an AUD recurrence.  For public drinkers  The legislation was associated with a significantly greater likelihood (*p*≤0.05) of AUD remission for:   - all participants, smokers (OR=1.88; 95% CI=1.16, 3.04) - men (OR=1.83; 95% CI=1.55, 2.15) - participants aged 18–29 (OR=1.75; 95% CI=1.13, 2.70) - participants aged 30-39 (OR=1.37; 95% CI=0.99, 1.88)   Smoke-free legislation was protective against first AUD onset overall, and for all subgroups except participants aged 40 or older, and was not associated with AUD recurrence in the subset of public drinkers. |
| **Multicomponent Intervention** | | | | | | | |
| S | Athanasopoulos et al. (2022), Australia (55) | ITS | Alcohol | LEPs:   - Kings Cross and Sydney CBD - Rest of NSW as comparator | Introduced end of February 2014  1. 1:30 AM lockout  2. 3 AM cessation of alcohol service  3. Prohibition on the granting of any new liquor licences  4. Ban on takeaway alcohol after 10.00 PM  5. Extension of ‘banning orders’ on ‘troublemaker’ patrons.  6. Ban on ‘shots’ and ready-to-drink beverages.  7. Introduction of RBL fee. | Time series analysis  Structural break analysis  Police recorded non-domestic assaults January 2009–March 2019 | **Criminal Justice:**  *Assault*  From January 2014 onwards, assaults fell immediately by 22% (a downward step) in Kings Cross (90% CI=15 to 28) and by 33% in the CBD (90% CI=19 to 47). Continued decline in Kings Cross (trend-break coefficient=−0.094, 90% CI=−0.192 to 0.005). Evidence of displacement of assaults to neighbouring precincts. However, there was net effect of reduced assaults post intervention. |
| M | Brännström et al. (2016), Sweden (56) | ITS | Alcohol | 288 Swedish municipalities | 1. Community coalitions steering group  2. On licensed premises supervision by police/municipality officials to ensure compliance regarding RSA | Fixed-effects spatial panel regression model with spatially lagged  dependent and independent variables  1996–2009 | **Criminal Justice:**  *Assault*  The direct, indirect, and total effects were −1.8% (95% CI=−4.4% to 0.8%), −5.8% (95% CI=−11.5% to −0.1%) and −7.6% (95% CI=−13.2% to −2.2%), respectively.  Averaged over time and across all municipalities, implementing one additional programme component reduces violent assaults in one typical municipality by nearly 8%. |
| S | Chamlin et al. (2014), USA (57) | ITS | Alcohol | Bars and taverns in San Marcos, Texas | Pre/post stage 1:  1. Extended hours of trade - midnight until 2 a.m.  2. Increase in patrol strength in the downtown district for June 2007–June 2009  Pre/post stage 2:  1. Termination of increased police patrols June 2007–May 2010 | ARIMA models  June 2007–May 2011  Unpublished calls for service (police data) including civilian complaint and police observations (not necessarily all arrests) | **Criminal Justice:**  *Public order offense*  Stage 1 intervention (but not stage 2) was associated with increase in verbal disturbance complaints (ω1o=0.50, p<.01).  Stage 1 intervention associated with an increase in physical disturbance complaints (ω1o=0.71, p<.001, an increase of 54%) and an increase in public intoxication complaints (ω1o=1.35, p<.05).  Stage 2 produced a further increase of 72% in physical disturbance complaints.  **Behavioural:**  *DUI*  Stage 1 intervention (but not stage 2) was associated with an increase in DUI complaints (ω1o=0.65, p<.01). |
| S | Coomber et al. (2021), Australia (58) | ITS | Alcohol | 5 LEPs in Queensland | TAFV policy  Stages:  1. Restricting alcohol sales to 3 am in designated precincts (July 2016)  2. Limiting annual number of extended trading permits (i.e., trading until 5 am). (February 2017)  3. Mandatory networked ID scanners (July 2017) | ARIMA/SARIMA models  July 2009–June 2019 | **Criminal Justice:**  *Assaults*  Significant declines in number of serious assaults in SNPs reported in:  Fortitude Valley. Across all HAH combined after every stage and cumulatively (SARIMA(1,0,0)(1,0,0,12); July 2016=−2.82, *p<*0.01; February 2017=−3.83, *p<*0.001; July 2017=−3.84, *p<*0.001; TAFV=−3.87, *p*<0.001). 8pm–11.59pm after stage 2,3 and cumulatively (SARIMA(0,0,0)(0,0,0,12); February 2017=−0.94, *p<*0.01; July 2017=−0.88, *p<*0.05; TAFV=−0.82, *p<*0.05). 3am–5.59am after every stage and cumulatively (SARIMA(0,0,0)(0,0,0,12); July 2016=−1.74, *p<*0.001; February 2017=−1.99, *p<*0.001; July 2017=−1.87, *p<*0.001; TAFV=−2.10, *p<*0.001).  15 Precinct Suburbs Combined.  3am–5.59am after every stage and cumulatively (SARIMA(1,0,0)(1,0,0,12); July 2016=−4.35, *p<*0.001; February 2017=−4.68, *p<*0.001; July 2017=−4.69, *p<*0.001; TAFV=−5.22, *p<*0.001).  Surfers Paradise. Across all HAH combined after stage 1 (SARIMA (3,1,0)(3,1,0,12); July 2016=−4.11, *p<*0.05).  Toowoomba. Across all HAH combined after every stage and cumulatively (SARIMA(0,0,0)(0,0,0,12); July 2016=−0.91, *p<*0.01; February 2017=−0.99, *p<*0.01; July 2017=−0.83, *p<*0.05; TAFV=−1.03, *p*  < 0.01).  No significant changes in Cairns or Townsville SNPs at any stage or cumulatively. |
| S | Curtis et al. (2017), Australia (59) | ITS | Alcohol | LEPs  Two in metropolitan areas: Melbourne CBD, Frankston  Two regional towns:  Geelong and Shepparton | The Fremantle liquor accord regulations included:  1. Patron bans  2. Responsible practice guidelines  3. Lockouts  4. Shatterproof glass  5. Education campaigns  6. Party bus legislation  7. Liquor licence freeze  8. Safe taxi rank  9. Policing operations  10. Night watch radio program  11. ID scanners  12. Fining strategy  13. RBL | ARIMA time series analysis  2000−2015 | **Criminal Justice:**  *Assault*  No significant effect on the count of assaults in any research sites during HAH2.  **Health:**  *Hospitalisations*  No significant effect on the rate of HAH1 ED presentations across all research sites |
| S | de Andrade et al. (2021), Australia (60) | ITS | Alcohol | LEPs  15 Queensland precinct suburbs | TAFV  1. Restricted alcohol sales to 3 am in designated precincts (July 2016)  2. Limited annual number of extended trading permits (i.e., trading until 5 am). (February 2017)  3. Mandatory networked ID scanners (July 2017) | Descriptive statistics and ARIMA/SARIMA models  July 2011–June 2019 | **Health:**  *Ambulance Callouts*  Average number of monthly callouts in HAH reduced by:   1. 26.2% in Fortitude Valley 2. 21.1% in Surfers Paradise 3. 4.3% in 15 precinct suburbs combined   Significant declines in monthly callouts:  *Fortitude Valley*  12am−2:59am and all HAH combined for cumulative policy stages; and 3am−5.59am at every stage and cumulatively.  *15 Precinct Suburbs Combined*  3am−5.59am after stage 3 |
| S | de Vocht et al. (2016), UK (61) | Other – Data Linkage | Alcohol | LEPs in England and Wales  N=319 Lower Tier Local Authorities | Cumulative impact zones (CIZs; areas in which applicants for a new alcohol licence must demonstrate how they will avoid threatening the licensing objectives) and licensing scrutiny (refusals of new license applications by lower tier local authorities) | Hierarchical growth modelling  Home Office Alcohol and Late-Night Refreshment Licensing data 2007–2012  Alcohol-related hospital admission and alcohol-related crime rates 2009–2015 | **Health:**  *Hospitalisations*  In 2009-2015: average decrease in alcohol-related hospital admissions of 0.5% per year.  Greater reduction in alcohol-related hospital admissions observed in local govt areas with CIZs and more intense licensing scrutiny (p=0.006).  Annual –2% change in alcohol-related hospital admission rates in the areas with the highest intensity policies (p<0.05; 95% CI: −3% to −2%). |
| S | de Vocht et al. (2017), UK (62) | Before and after | Alcohol | LEPs in England and Wales  N=284 Lower Tier Local Authorities | Cumulative impact zones (CIZs; areas in which applicants for a new alcohol licence must demonstrate how they will avoid threatening the licensing objectives) and licensing scrutiny (refusals of new license applications by lower tier local authorities) | Growth curve analysis  2009−2015 | **Criminal Justice:**  *Assaults, sexual assaults and related offences, and public order offences* In the most ‘intense’ areas, alcohol-related violent crime rates reduced (~ 6.1 per 1000 people in 2009 to 4.9 per 1000 people in 2013, and back to 5.2 per 1000 people in 2014). There were smaller reductions in the ‘passive’ areas (3.9/1000 in 2009 to 3.3/1000 in 2013 and to 3.5/1000 in 2014) .  For alcohol-related sexual assaults, until 2013 rates decreased in most areas with 0.10/1000 people across the period in ‘passive’ areas compared with a reduction from 0.15 to 0.14 /1000 people in the most ‘intense’ areas). Rates increased most rapidly post-2013 in areas with most intense policy.  For public order offences, there were similar trends of stronger reductions in areas with more intense policies until 2013, but no steep increase post-2013 was observed. |
| S | de Vocht et al. (2017), UK (63) | ITS | Alcohol | Nightclubs and restaurants in 91 principal authorities in England - 5 intervention areas (Kingston upon Thames, Derby, Enfield, Southwark and North Tyneside); and 86 control sites. | Cumulative impact zones (CIZs; areas in which applicants for a new alcohol licence must demonstrate how they will avoid threatening the licensing objectives) and licensing scrutiny (refusals of new license applications by lower tier local authorities) | Novel Bayesian synthetic time series  Home Office Alcohol and Late-Night Refreshment Licensing data for 2007–2012  2009–2015 alcohol-related hospital admissions, violence and sexual crimes, and anti-social behaviour obtained from the Local Alcohol Profiles for England i.e 2007−2015 | **Criminal Justice:**  *Assault*  Reduction in violent crimes, especially up to 2013 (–4.6%, 95%CI −10.7% to 1.4%).  *Sexual assault*  Weak evidence of an effect on sexual crimes up 2013 (–8.4%, 95%CI −21.4% to 4.6%).  *Public Order Offense*  No evidence of an effect on antisocial behaviour because of a change in reporting.  **Health:**  *Hospitalisations*  Reduction in alcohol-related hospital admissions of 6.3% (95% CI: −12.8% to 0.2%) |
| S | de Vocht et al. (2020), UK (64) | Quasi-randomized control trial | Alcohol | Three English local areas of 1,000−15,000 people each. | 3 natural experiments in 3 local areas:  Case Study 1: Liquor licensing decision to revoke license and close a nightclub.  Case Study 2: Liquor licensing decision to close a restaurant and cocktail bar.  Case Study 3: Development of new local licensing guidance and increased venue inspectors. | Bayesian structural time–series  Case Study 1: January 2010−September 2014  Case Study 2: 2015−2017  Case Study 3: 2008−2014 | **Criminal Justice:**  *Assault and Sexual Assault*  Study 3: No significant changes, but small increase in domestic violence incidents when program finished.  *General Crime*  Study 1 & 2: No significant changes  *Public Order Offense*  Study 1: Initial, but temporary reduction in reported antisocial behaviour. 4-month impact of 18% (95% CI=37%, 4%), equating to 60 averted incidents.  Study 2: No significant change.  Study 3: Weak evidence of a reduction in drunk and disorderly behaviour incidents (12-month impact of -4.2%; 95% CI=109% +23%), equating to <1 averted incident/month.  Study 3: Weak - moderate evidence of a small impact on reported drunk and disorderly behaviour; equating to 1-2 averted incidents/month.  *Other Offenses (antisocial behaviour)*  Study 1: Strong evidence of an immediate, but short-term impact.  **Health:**  *Hospitalisations and Ambulance Attendances*  Study 1: No significant changes |
| M | Devilly et al. (2019), Australia (65) | Before and after | Alcohol | LEPs | TAFV policy  Stages:  1. Restricting alcohol sales to 3 am in designated precincts (July 2016)  2. Limiting annual number of extended trading permits (i.e., trading until 5 am). (February 2017)  3. Mandatory networked ID scanners (July 2017) | - Descriptive statistics - Mann-Whitney U-Test - Effect Sizes - t-tests   Study 2: 31st October 2015 to 26th June 2016; Study 3: Thursday, Friday, and Saturday nights from 16th September 2016 to 7 July 2017. | **Criminal Justice:**  *Assault*  No significant pre-post change in count of assaults  *Public Order Offense*  No significant pre-post change in count of public order offences  *Drug Offenses*  Increase recorded  2016: 24/313 (7.67%) versus  2017: 35/267 (13.11%). |
| S | Donnelly et al. (2017), Australia (66) | ITS | Alcohol | 2 designated LEPs:   - Kings Cross and Sydney CBD - Proximal (Pyrmont, Surry Hills) and distal (Newtown, Double Bay, Bondi Beach, Coogee) areas | 1. 1:30 AM lockout  2. 3:00 AM cessation of alcohol service  3. Prohibition on the granting of any new liquor licences  4. Ban on takeaway alcohol after 10PM  5. Extension of ‘banning orders’ on ‘troublemaker’ patrons.  6. Ban on ‘shots’ and any ready-to-drink beverages.  7. Introduction of RBL fee. | ARIMA  Police recorded non-domestic assaults January 2009 – September 2016 | **Criminal Justice:**  *Assault*  Significant reductions post intervention in both Kings Cross (49%) and Sydney CBD (13%). Geographical displacement of non-domestic assaults evident in both the PDA (2%) and the DDA (up 17%); However, there was net effect of reduced assaults in the combined Kings Cross and Sydney CBD Precincts (930 fewer non-domestic assaults). |
| S | Fell et al. (2017), USA (67) | Before and after | Alcohol | Intervention communities: 1. Monroe County, NY (control site, Onondaga County, NY), 2. Cleveland, Ohio (control site, Toledo, Ohio).  30 problem bars per community identified through POLD data and calls for service data. 10/30 problem bars per intervention community were randomly assigned to receive the intervention and matched with 10 problem bars from control sites. | A responsible beverage service and enforcement program, including:  1. Collection of places of last drink data for drivers arrested for alcohol-related offenses to determine problem venues  2. Letter writing & bar serving assessments by alcohol beverage control (ABC) officers to raise awareness and cooperation among problem bars,  3. RBS training,  4. Stepped-up alcohol law enforcement. | Descriptive statistics  Pre/post regression analysis (undefined)  8 different components in this study - each with spans of time.  August 2008−December 2009 | **Health:**  *Road traffic accidents*  No statistically significant changes in pre-post or between intervention and control bars.  **Criminal Justice:**  *Driving offences*  Monroe County, NY: Significant decline in proportion of 21−34 yr olds arrested for DUI post-intervention (53% to 45%, *p=*.002); compared to a non-significant increase in control site (49% to 51%, *p=*.65).  **Behavioural:**  *Self-reported drink driving*  Monroe County, NY: Significant increase compared to control site first post period - second post period only (β=1.04, *p=*.0035). Significant decrease reported from pre- intervention to first post-intervention (β=1.76 *p=*.0001).  Cleveland, Ohio - Short-term positive effect (pre-post intervention, β=−1.33, *p=*.002). Decrease in proportion of drivers who reported being intoxicated before driving in Cleveland relative to control site, in the 1st follow up period (β=−0.68, *p=*.055), but not the 2^nd^ follow up period (β=−0.40, *p=*.268). |
| W | Ford et al.  (2018), NZ (68) | Cross-sectional - one group | Alcohol | NZ-wide  Licensed premises patrons that present to EDs  2013: N=3,400 ED attendees  2017: N=3,721 ED attendees | Sale and Supply of Alcohol Act 2012 (SSAA), included:  1. Trading hour limits of 8am-4am for on-licenses, and 7am-11pm for off-licenses, and a provision enabling territorial authorities to develop a local alcohol policy through public consultations.  2. New RBL fees  3. Small changes to licensing procedure.  4. Requirement to consider the impact of alcohol outlets on amenity and good order near location.  5. Make one-way door policies legally enforceable. | Chi-square tests for  categorical outcomes  Mann-Whitney U  tests or t-tests for continuous outcomes.  Three-week waves of data collection in 2013 and 2017 | **Health:**  *Hospitalisations*  2013−2017 there was a non-significant (p=.41) reduction in the proportion of alcohol-related ED attendees, from 253/3400 (7.4%) to 258/3721 (6.9%). |
| W | Fulde et al. (2015), Australia (69) | Before and after | Alcohol | 2 designated LEPs:   - Kings Cross and Sydney CBD, as well as proximal (Pyrmont, Surry Hills) and distal (Newtown, Double Bay, Bondi Beach, Coogee) | 1. 1:30 AM lockout  2. 3:00 AM cessation of alcohol service  3. Prohibition on the granting of any new liquor licences  4. Ban on takeaway alcohol after 10.00pm.  5. Extension of ‘banning orders’ on ‘troublemaker’ patrons.  6. Ban on ‘shots’ and any ready-to-drink beverages.  7. Introduction of RBL fee.  Other initiatives introduced in Sydney during the 1^st^ year lockout introductions:  1. Visibly increased police presence and monitoring.  2. Introduction of ID scanning on entry into some venues; and sharing of information by venues.  3. Volunteer nocturnal patrols. | ED presentations for alcohol related injuries | **Health:**  *ED Admissions*  Significant decrease in 12 months pre licensing regulations vs. 12 months post.  Number of seriously injured presentations during HAT=relative reduction of 24% (p<0.05) |
| M | George et al. (2018), USA (70) | Non-randomized control trial | Alcohol | LEPs | A three-component community prevention intervention:  1. RSA  2. Onsite law enforcement  3. Media Campaign | Structured content analysis (descriptive)  2-tailed T-test  2011−2014 | **Criminal Justice:**  *Driving Offences*  Declines in:  - monthly percentage of drivers arrested for DUI (30% to 8%);  - alcohol-involved crashes in the 12-month postintervention period; and  - DUI crashes following 12 months of intensive work with licensed establishments in contrast to the DUI crashes for the rest of South Carolina. |
| S | Hoffman et al. (2017), Australia (71) | Retrospective cohort | Alcohol | Licensed entertainment venues in Newcastle, NSW CBD  N=152 patient attendances  74% males | Close of business reduced from 5 a.m. to 3 a.m. (and by subsequent negotiation to 3.30 a.m.) and the admission of new patrons (lockouts) was limited to 1 a. m. (and by subsequent negotiation to 1.30 a.m.). | Segmented Regressions (negative binomial and Poisson distributions)  1 January 2003− 31 December 2015  Intervention in 2008. | **Health:**  *Hospitalisations*  Oral and maxillofacial admissions increased significantly at 14% per annum pre- intervention, then decreased at a rate of 21% per annum post-intervention.  Pre-post rates were found to be significantly different (RR 0.69, P<0.001; i.e., 31% relative RR reduction). A significant change between estimated numbers for pre- and post-intervention was also seen (RR 1.87, p=0.002). |
| M | Hughes et al. (2018), Australia (72) | Other – Qualitative, focus groups | Alcohol | 2 designated LEPs:  Kings Cross/Potts Point and Newtown.  *Focus groups:*  Participants n=21 (11 Kings Cross/Potts Points, 10 Newtown) | 1. 1:30 AM lockout  2. 3:00 AM cessation of alcohol service  3. Prohibition on the granting of any new liquor licences  4. Ban on takeaway alcohol after 10PM  5. Extension of ‘banning orders’ on ‘troublemaker’ patrons.  6. Ban on ‘shots’ and any ready-to-drink beverages.  7. Introduction of RBL fee. | Thematic Analysis | **Behavioural:**  *Aggression*  Reduction in violence, aggression, “fights” in Kings Cross, but reported increase in violence in Newtown. Tentative evidence of displacement effect. |
| S | Kypri et al. (2011), Australia (73) | ITS | Alcohol | LEP in Newcastle CBD, NSW, with Hamilton as control site | Newcastle CBD intervention:  1. Earlier closing (3am) and lockout (1am), relaxed to 3:30 and 1:30 months later.  2. Licensees required to adopt management plans, and subject to compliance audits  3. A dedicated RSA officer from 11pm  4. No shots after 10pm  5. Cessation of alcohol service 30 min before closing  6. No drink stockpiling  7. Adopting a shared radio procedure  8. Staff had to be notified of the conditions  Hamilton intervention:  1. No earlier closing times but lockout was implemented. | Negative binomial regressions  January 2001–September 2009  Pre-intervention: January 2001–March 2008  Post intervention: April 2008–September 2009 | **Criminal Justice:**  *Assaults*  In Newcastle CBD, recorded assaults fell from 99/quarter pre-intervention to 68/quarter post-intervention (IRR=0.66, 95% CI: 0.55–0.80). In Hamilton, assault rates were 23.4 (pre) and 26/quarter (post); (IRR: 1.02, 95% CI: 0.79–1.31). The relative reduction attributable to the intervention was 37% (IRR=0.63, 95% CI: 0.47–0.81), equivalent to preventing approximately 33 assault incidents/quarter. |
| S | Kypri et al. (2014), Australia (74) | ITS | Alcohol | LEP in Newcastle CBD, NSW, with Hamilton as control site | Newcastle CBD intervention:  1. Earlier closing (3am) and lockout (1am), relaxed to 3:30 and 1:30 months later.  2. Licensees required to adopt management plans, and subject to compliance audits,  3. A dedicated RSA officer from 11pm until closing,  4. No shots after 10pm  5. Cessation of service of alcohol 30 min before closing  6. No drink stockpiling,  7. Adopting a shared radio procedure  8. Staff had to be notified of the conditions  Hamilton intervention:  1. No earlier closing times but lockout was implemented | Negative binomial regressions  January 2001–March 2013  Pre-intervention: January 2001–March 2008  Post-intervention:   1. April 2008–September 2009 2. October 2009–March 2013. | **Criminal Justice:**  *Assaults*  In Newcastle CBD, recorded assaults fell from 99/quarter pre-intervention to 68 per quarter post-intervention period 1 (IRR=0.67, 95% CI: 0.55–0.82); and 71/quarter (IRR=0.68, 95% CI: 0.55–0.85) in post-intervention period 2. In Hamilton, assault rates were 23/quarter (pre), 24/quarter (post 1) and 22/quarter (post 2). |
| S | Kypri et al. (2020), Australia (75) | ITS | Alcohol | 2 designated LEPs:  Kings Cross and Sydney CBD, as well as surrounding areas (Pyrmont, Surry Hills, Newtown, Double Bay, Bondi Beach, Coogee), to study displacement | The NSW ‘lock-out-laws’ included:  1. 1:30 AM lockout  2. 3:00 AM cessation of alcohol service  3. Prohibition on the granting of any new liquor licences  4. Ban on takeaway alcohol after 10PM  5. Extension of ‘banning orders’ on ‘troublemaker’ patrons.  6. Ban on ‘shots’ and any ready-to-drink beverages.  7. Introduction of RBL fee. | ITS  Poisson regression framework  24^th^ January 2009–23^rd^ February 2019 | **Criminal Justice:**  *Assaults*  Following introduction of liquor regulations, assaults fell 38% in Kings Cross (IRR for step change=0.62, 95% CI=0.49, 0.79) and 10% in the CBD (IRR=0.90, 95% CI=0.80, 0.99). Continued decline evident in Kings Cross (IRR for slope=0.990, 95% CI=0.982, 0.998) and later an increase in neighbouring areas (IRR for slope=1.006, 95% CI=1.001, 1.011) and earlier in the evenings in both Kings Cross and adjacent areas.  Geographical displacement assaults evident, however, there was net effect of reduced assaults in the combined Kings Cross and CBD Precincts (627 assaults over 60 months post-intervention, i.e., 10 fewer per month) |
| S | Livingston et al.  (2021), Australia (76) | ITS | Alcohol | Queensland LEPs | TAFV policy stages:  1. Restricting alcohol sales to 3 am in designated precincts (July 2016)  2. Limiting annual number of extended trading permits (i.e., trading until 5 am). (February 2017)  3. Mandatory networked ID scanners (July 2017) | ARIMA/SARIMA models | **Health:**  *Hospitalisations*  State-wide, there was a significant decline in ED injury presentations following the introduction of mandatory ID scanners in July 2017 (β=-0.21, 95% CI=-0.39, -0.04, *p=*0.02), estimated to be a 4.1% reduction in monthly high-alcohol hours attendances. No other significant effects of intervention stages across Brisbane or Statewide, for hospitalisations or ED presentations. |
| S | Menéndez et al. (2015), Australia (77) | ITS | Alcohol | 2 designated LEPs:  Kings Cross and Sydney CBD, as well as proximal (Pyrmont, Surry Hills) and distal (Newtown, Double Bay, Bondi Beach, Coogee) | The NSW ‘lock-out-laws’, included:  1. 1:30 AM lockout  2. 3:00 AM cessation of alcohol service  3. Prohibition on the granting of any new liquor licences  4. Ban on takeaway alcohol after 10PM  5. Extension of ‘banning orders’ on ‘troublemaker’ patrons.  6. Ban on ‘shots’ and any ready-to-drink beverages.  7. Introduction of RBL fee. | Structural time series  January 2009– September 2014 | **Criminal Justice:**  *Assault*  Statistically significant reductions in assaults in Kings Cross (32%), Sydney CBD (40%), and rest of NSW (9%). |
| S | Menendez et al. (2015), Australia (78) | ITS | Alcohol | 2 designated LEPs:  Kings Cross and Sydney CBD, as well as proximal (Pyrmont, Surry Hills) and distal (Newtown, Double Bay, Bondi Beach, Coogee) | The NSW ‘lock-out-laws’, included:  1. 1:30 AM lockout  2. 3:00 AM cessation of alcohol service  3. Prohibition on the granting of any new liquor licences  4. Ban on takeaway alcohol after 10PM  5. Extension of ‘banning orders’ on ‘troublemaker’ patrons.  6. Ban on ‘shots’ and any ready-to-drink beverages.  7. Introduction of RBL fee. | Structural time series  Serious assaults (ABH and GBH)  1^st^ January 1996–31^st^ December 2013 | **Criminal Justice:**  *Assault*  Significant reduction of 31.27% of ABH [parameter estimate 0.38 with 95% confidence interval (CI)=0.65, –0.10)]. 39.70% reduction in GBH over a shorter period July 2008-July 2012 (parameter estimate 0.51 with 95% CI=0.69, – 0.33). |
| S | Menendez et al. (2017), Australia (79) | ITS | Alcohol | 2 designated LEPs:  Kings Cross and Sydney CBD, as well as proximal (Pyrmont, Surry Hills) and distal (Newtown, Double Bay, Bondi Beach, Coogee) | The NSW ‘lock-out-laws’, included:  1. 1:30 AM lockout  2. 3:00 AM cessation of alcohol service  3. Prohibition on the granting of any new liquor licences  4. Ban on takeaway alcohol after 10PM  5. Extension of ‘banning orders’ on ‘troublemaker’ patrons.  6. Ban on ‘shots’ and any ready-to-drink beverages.  7. Introduction of RBL fee. | Structural time series  2009–2015  Daily time periods:   - 6pm–1.29am - 1.30am–2.59am - 3am–6am | **Criminal Justice:**  *Assault*  Following the introduction of liquor regulations, assaults reduced by 45% in Kings Cross (β=-0.599, 95% CI=-1.107, -0.091) and 22% in Sydney CBD (β=0.260, 95% CI=-0.397, –0.123)  In Kings Cross reductions were observed across all daily time periods; in Sydney CBD reductions only observed in the 2^nd^ and 3^rd^ time periods.  No evidence of displacement of assaults to neighbouring entertainment precincts. |
| S | Miller et al. (2011), Australia (80) | ITS | Alcohol | LEP | Intervention included:  1. Night-Watch Radio Program: Connection of security staff via radio with relevant personnel.  2. ID scanners  3. Just Think: Local celebrities endorsing safe drinking patterns.  4. Maximum police visibility during high-risk hours.  5. Improved radio contact between police and licensees.  6. Safe Streets Taskforce: Increase police visibility.  7. Undercover police at licensed venues | ARIMA  2005–2009 | **Health:**  *Hospitalisation*  Intervention not associated with reduced alcohol-related attendances at the ED.  The time-series analyses indicated that ID scanners (z=2.66, p<0.001) and the Just Think awareness campaign (z=4.21, p<0.001) were significant predictors of increases in alcohol-related injury presentation rates to the ED.  Other interventions had no significant effect on ED admissions. None of the interventions reduced ED admissions*.* |
| S | Miller et al. (2014), Australia (81) | ITS | Alcohol | LEPs in Newcastle, NSW | **Geelong:**   1. ID scanners. 2. Increased policing. 3. CCTV. 4. Taxi ranks. 5. RBL   **Newcastle**  1. Lockout laws.  2. Alcoholic drink restrictions.  3. RSA. | ARIMA/SARIMA models  2005–2011 | **Health:**  *Hospitalisation*  Significant reductions in injury-related presentations during high-alcohol risk times (344 attendances per year, p<0.001). None of the interventions deployed in Geelong were associated with reductions in ED presentations. |
| W | Munn et al. (2016), Canada (82) | Descriptive case report | Alcohol and illicit drugs | 7-day Shambhala outdoor music festival | Intervention included:  1. Mobile outreach teams.  2. Distribution of AOD educational materials.  3. Drug checking facilities.  4. A dedicated women’s space, and a “Sanctuary” area that provided non-medical peer support for overwhelmed guests.  5. Medical and harm reduction services marquees. | Descriptive statistics | **Health:**  *Ambulance attendances*  High patient presentation rate (20.8%),  Low ambulance transfer rate (0.194)  Low percentage of patients transferred (0.93%)  High acuity transfers prevented (53)  Dedicated on-site harm reduction and medical services minimise the effects of a >13-fold increase in population on local health infrastructure. 13 patients were transported off-site by ambulance. The cumulative ambulance transfer rate=0.194 transfers/1,000 cumulative attendees. Cumulative percentage of patients transported by ambulance =0.93%. Of the 102 highest acuity encounters, 53 received care on site, averting a visit to hospital, reducing ambulance transfers and ED impact. |
| W | Navarro et al. (2013), Australia (83) | Randomized controlled trial | Alcohol | Licensed entertainment venues in regional NSW. | 1. Letter from mayor to licensees asking to brief security staff and ensure service of alcohol requirements met.  2. Local media awareness in the community.  3. Police visibility during the problem weekend.  4. Feedback on problem incidents the weekend after through media and stakeholder meetings. | Generalised estimating equations, with a difference-in-difference base model  1^st^ January 2001–31^st^ December 2009.  Intervention: 23^rd^ May 2008–31^st^ December 2009 | **Criminal Justice:**  *Assault*  There was no effect on assaults (excluding sexual assaults, measured separately).  *Sexual assault*  The intervention had “a small, but statistically significant, effect on alcohol-related sexual assaults: a 64% reduction in the experimental communities which is equivalent to five fewer alcohol-related sexual offences”. |
| M | Norström et al.  (2013), Sweden (84) | ITS | Alcohol | LEPs | 1.Community coalition steering group.  2.Staff training in responsible beverage service.  3. Stricter enforcement of existing alcohol laws. | ARIMA modelling  January 1994–September 2000 | **Criminal Justice:**  *Assault*  Estimated 29% reduction in assaults. When using the alternative control area, the estimated intervention effect drops to 24%. If no control area is included, the effect is down to 21%. Seasonal differencing of the data also tended to lower the estimated intervention effect. |
| S | Norström et al.  (2018), Sweden (85) | ITS | Alcohol | Six nightclubs in Visby, Sweden | 1. Opening hours extended by 1-hour, postponing closing time from 2 to 3 a.m.  2. RSA Training.  3. Intensified cooperation between  police and nightclubs. | Violence rates during the intervention period (week 24–week 33, 2014) were compared with the violence rate in the corresponding period in Visby in 2010–2013. | **Criminal Justice:**  *Assault*  Effect was strongly statistically significant with a reduction of 3.336 reported assaults at night per week compared to the pre-intervention period - a decrease of 71%. of police-reported assaults that had occurred at night (midnight–6 a.m.) |
| W | Paschall et al. (2021), Mexico (86) | Case series | Alcohol | Licensed premises in LEPs | Introduction of restricted bar opening hours - 2am close instead of 4am close AND introduction of a 10PM cut-off for alcohol sales by off-premise stores | Descriptive statistics from stakeholder surveys  Monthly observation/mystery shopper qual data  Data was collected on a monthly basis from September 2018–January 2019, May 2019, and July 2019–February 2020. | **Health:**  *General Injuries*  Bar owners reported significant reduction in customers needing medical help (13.2% vs 0%) from 2018 to 2019, but bartenders or security staff did not experience this. ED and ambulance personnel also reported a significant reduction in drunk or injured customers - mean of 0.32 to 0.18.  **Behavioural:**  *Aggression*  Bar owners reported significant reduction in physical fights between customers (15.8% to 0%), less property damage (12.8% to 0%) and less need for police because of riots (15.8% to 0%). No significant change for bartenders or security staff. ED and ambulance personnel also reported significant reduction in physical fights between customers (mean of 0.35 to 0.23 between 2018 and 2019). |
| S | Pliakas et al. (2018), UK (87) | ITS | Alcohol | LEPs  One Local Authority area in London  N=752 alcohol license applications | Introduction of 7 cumulative impact zones (CIZ), including  closing times for businesses applying for new/variation alcohol licences, including:    1. Off-licences -11pm.  2. Night clubs - 1 am Sund to Thurs, 2 am Fri and Sat.  3. Restaurants, cafes, and bars - 11 pm Sunday to Thursday, midnight Friday and Saturday.  4. Hot food and drink from takeaways - midnight Sunday to Thursday, 1am Friday and Saturday.  5. 24h sales of alcohol to hotel residents. | ITS analysis  2008–2014 | **Criminal Justice:**  *General crime, public order offense*  A significant initial decline in overall crime rates (CIZs=−12.2%, 95% CI=−18.0%, −6.1%; non CIZs=−8.0%, 95% CI=−14.0%, −1.6%) were only partially reversed by small, longer-term increases.  Immediate impacts on anti-social behaviour were not statistically significant and increased over the longer term both in CIZs and non-CIZs and across the Local Authority.  **Health:**  *Ambulance attendances*  No significant impacts on alcohol-related ambulance call-out rates. |
| S | Skardhamar et al.  (2016), Norway (88) | ITS | Alcohol | Licensed premises in Oslo city centre (intervention site) and two control areas with a high density of licensed premises | SALUTT included all the key components from STAD (see Norström et al., 2013)  In addition:  1. Places more emphasis on dialogue relative to sanctions, and a less central role played by the police (given police in Norway are not in charge of licensing and control).  2. The SALUTT area was smaller than that of Stockholm; restricted to an area where these problems were prevalent. | ARIMA models  1st January 2008–21st August 2015 | **Criminal Justice:**  *Violence*  The estimate in the comparison  with the first control site suggests a negative point estimate (−0.22), with a very high p-value, and therefore no statistical significance of effect on violence.  The estimate in the comparison with the second control site suggests a similar point estimate (−0.15), which negligible statistical significance (p=0.73).  Thus, The SALUTT programme had no statistically significant effect on violence. |
| S | Taylor et al. (2021b), Australia (89) | Other - Data Linkage | Alcohol | Queensland- 9 NEPs across eight QLD cities: Airlie Beach CBD; Bundaberg CBD; Fortitude Valley; Inner West Brisbane; Ipswich CBD; Mackay CBD; Rockhampton CBD; Surfers Paradise CBD; Toowoomba CBD. | TAFV policy stages:  1. Restricting alcohol sales to 3 am in designated precincts.  2. Limiting annual number of extended trading permits (i.e., trading until 5 am).  3. Mandatory networked ID scanners. | Multilevel modelling:   - Poisson family regression   January 2010–July 2018 | **Criminal Justice:**  *Assaults*  Lower outlet density and earlier trading hours is associated with decreased assault; this association is stronger in precincts with trading hours ending at 5am compared to 3am (Incidence rate ratio (IRR)=1.01, p=0.03).  Venues closing before 12am was associated with reduced numbers of assaults (IRR=0.97, p=0.04), while venues closing between 12:01am-3am and 3:01am-5am were associated with increased assaults (IRR=1.02, p<0.01; IRR=1.01, p=0.02). |
| S | Trolldal et al. (2013), Sweden (90) | Before and after | Alcohol | 237 municipalities, with a control area in the central part of Stockholm | A multi-component Responsible Beverage Service (RBS) programme, consisting of:  1. RBS training.  2. Community coalition steering group.  3. Supervision to ensure fidelity to the RBS training | A fixed-effects panel data regression model:   - Least-square dummy variable model for longitudinal data   1996–2009 | **Criminal Justice:**  *Assaults*  Each extension of the programme, by one component, associated with a significant 3.1% reduction in assaults [95% confidence interval (CI): −0.058 to −0.004].  The community coalition steering group had the most significant effect on assaults (95% CI: −0.153 to −0.032).  No significant effect was found regarding RBS training, or premises supervision. |
| M | Wiggers et al. (2021), Australia (91) | Before and after | Alcohol | Newcastle LEP, New South Wales | The Newcastle CBD intervention:  1. Closing (3am) and lockout (1am), relaxed to 3:30 and 1:30 months later.  2. Licensees to adopt management plans, and compliance audit.  3. A dedicated RSA officer from 11pm until closing.  4. No shots after 10pm.  5. Cessation of alcohol service 30 min before closing.  6. No drink stockpiling.  7. Radio procedures and all staff to be notified of the conditions. | Descriptive statistics:   - Logistic regression analysis - Chi-square and P-value tests   March–May 2010 and May–June 2013 | **Criminal Justice:**  *Violence*  The reduction in violence wasn’t significant for any of the groups. Significant reduction in the number of participants who reported witnessing or being involved in physical violence (n=73 to n=47) and a reduction in the number of participants who reported the alcohol-involvement in physical violence (n=71 to n=38).  *General Crime*  Significant decrease in proportion of general participants who said that 70% or more of crime in the precinct is alcohol-related (2010: 46.5%; 2013: 37.5%; *p*=0.009). This trend was also observed amongst night-time visitors (2010: 37.1%; 2013: 27.4%; *p*=0.05). Significant decrease in proportion of participants that stated they would never walk alone after dark in the precinct (2010: 69%; 2013: 59.2%, OR 0.59, *p=*0.002).  **Behavioural:**  *Risky Consumption Practices*  Significant decrease in proportion of participants who agreed that alcohol misuse was a problem in the precinct (2010: 89.9%; 2013: 84.9%; OR 0.58; *p=*0.02). This trend was also identified in visitors to the precinct (2010: 86.5%; 2013: 74.5%, OR 0.38, *p=*0.006) and those that reported consuming alcohol at risky levels (2010: 87.6%; 2013:77.4%, OR 0.43, *p=*0.009). |
| S | Xu et al. (2012), USA (92) | ITS | Alcohol | On premises outlets and other licensed venues in New Orleans | A series of policies decreed in 1997:  1. Increased license fee.  2. Additional enforcement staff.  3. Expanded powers for the alcohol license board. | Multilevel spatiotemporal change-point/hierarchical models  1994–2004 | **Criminal Justice:**  *Assaults*  There was a slight reduction in the positive association between on-sale alcohol outlet density and crime rate, however the effect was not statistically significant. |
| S | Zhang et al. (2015), USA (93) | ITS | Alcohol | Licensed entertainment premises in Buckhead compared with 2 other cluster areas in Atlanta (Midtown and Downtown) | 1. Alcohol outlet density restrictions  2. Restricting the hours when alcohol could be sold  3. Enforcing laws prohibiting alcohol sales to minors. | Multilevel regression  Preintervention period 1997–2002, postintervention period 2003–2007 | **Criminal Justice:**  *Violence*  During the preintervention period, the impact of exposure to alcohol outlets on exposure to violent crime was 2 to 4 times greater in Buckhead (regression coefficient [RC]=0.84) than in either Midtown (RC=0.18) or Downtown (RC=0.32).  During the postintervention period, the impact of exposure to alcohol outlets on exposure to violent crime was about 2 to 5 times greater in Buckhead (RC=0.65) than in either Midtown (RC=0.12) or Downtown (RC=0.27). |
| **Staff and Venue Intervention** | | | | | | | |
| W | Charlebois et al.  (2017), USA (94) | Non-randomized control trial | Alcohol | 4 gay bars in San Francisco, California | Intervention included:  1. Freely accessible water.  2. In-bar media (large posters) messaging campaign promoting the use of water.  3. Normative feedback of patron BAC with handheld breathalyser. | Descriptive statistics and:   - Pearson χ 2 - Fisher’s Exact test   Pre-intervention - Sep–Nov 2012. Post Intervention- Feb 2013–Aug 2014 | **Behavioural:**  *Risky consumption practices*  30% of intervention bar participants had BAC% levels over the legal driving limit (0.08%) compared to 43% of control bar participants, p<0.001.  78% of intervention bar participants were above the AUDIT-C cut-off for hazardous drinking compared to 87% in control bars, p<0.001. |
| M | Farrimond et al.  (2018), United Kingdom (95) | Qualitative Interviews and Focus groups | Alcohol | LEPs in Torquey and Weymouth  Sample: bar staff, security staff and security company  *Interviews*  Bar staff (n=12)  Security staff (n=18)  *2 x Focus groups*  Security staff (n=22)  Security company managers (n=3) | Voluntary adoption on the doors of venues of hand-held breathalysers (to deter excessive drunkenness and pre-drinking) | Thematic analysis  Torquay: December 2014  Weymouth: September−December 2016 | **Behavioural:**  *Aggression*  Conflicting views on the effectiveness of intervention to reduce aggression. Some security staff reported the intervention reduced/disrupted aggression at door, but others reported it was not useful. |
| S | Ham et al.  (2022), Colombia (96) | Randomized controlled trial | Alcohol | Neighbourhood bars in 4 localities in Bogota | Intervention included:  1. Provide bartenders with standardized practices that promote RSA.  2. Train bartenders on defusing conflicts that may result in alcohol-related violence. | Descriptive statistics and difference-in-difference estimations  January 2014–February 2019 | **Criminal Justice:**  *Assault*  No statistically significant changes in the number of reported brawls after the Good Drinks program for different specifications, procedures, treatment definitions, or compliance and treatment differences. |
| S | Moore et al.  (2017), UK (97) | Randomized controlled trial | Alcohol | Licensed entertainment venues e.g., public houses, nightclubs, hotels in Wales | The SMILE intervention:  1. Risk audit by EHPs to identify known risks of violence  2. Follow-up audit in premises where serious risks had been identified.  3. EHPs administer advice to staff on how to reduce risks, incl. online materials, educational videos, and related material. | Descriptive statistics | **Criminal Justice:**  *Assault*  The intervention resulted in an increase in police recorded violence [hazard ratio (HR)=1.34, 95% confidence interval 1.20 to 1.51]-compared to normal practice. Exploratory analyses suggested that reduced violence was associated with greater intervention dose (follow-up visits)- an effect constant across the follow-up period. |
| W | Zawisza et al.  (2020), USA (98) | Other – Observational Study | Alcohol | LEP in Little Rock, Arkansas | Implementation of effective place managers and capable guardians (e.g., police, bouncers, door attendants and barricades) | Qualitative observations  6-week period in the fall of 2012 on Friday nights/Saturday mornings | **Behavioural:**  *Aggression*  The intervention significantly limited the opportunities for aggression. Only 3 instances of aggression witnessed during observation period. |

| **Patron Survey and Assessment Feedback** | | | | | | | |
| --- | --- | --- | --- | --- | --- | --- | --- |
| M | Baldin et al.  (2018), Brazil (99) | Randomized controlled trial | Alcohol | 31 nightclubs in the city of São Paulo | Street-intercept survey including:  1. Nightclub patrons’ alcohol consumption, and the risks associated with amount consumed.  2. Risk behaviours  3. Alcohol expenses.  4. Drink driving.  5. Risk classification of AUDIT.  Intervention group received personalised normative feedback on alcohol related risks, and tips to reduce related harm | Generalised linear model (with ITT analysis)  Descriptive statistics:   - Pearson’s chi-square test - Fisher’s exact test   2013 | **Behavioural:**  *Risky consumption practices*  Reduction in weekly binge drinking of 38% (p=0.026) at 6-month follow-up for intervention group. No significant effect was observed for the control group (p=0.062). |
| W | Monezi et al.  (2017), Brazil (100) | Before and after | Alcohol | Pubs, nightclubs, and restaurants from 30 randomly selected locations from three main areas of São Paulo Center, South and West. | Patron survey included modules on:  1. How alcohol affects the Brazilian traffic law regarding alcohol consumption.  2. How alcohol affects driving capacity.  3. How alcohol can increase the risk of car accidents.  4. The factors that can increase BAC.  5. Strategies to reduce the effects of alcohol. | Z Score  X^2^ test for categorical variables  Mann Whitney test  ANOVA | **Criminal Justice:**  *Driving offenses*  The survey intervention showed some positive indication, but there wasn’t a significant reduction observed in the use of alcohol before driving.  Participants reported a reduction in drunk driving episodes. |

***Abbreviations:*** ABH – Aggravated bodily harm; ARIMA - Autoregressive Integrated Moving Average; AUDIT - Alcohol Use Disorders Identification Test; BAC – Blood Alcohol Concentration; CBD – Central Business District; CI – Confidence Interval; CIA – Clean indoor air policy; CIZ – Cumulative Impact Zone; CIZ – Cumulative Impact Zone; DDA – Distal displacement area; DUI – Driving under the influence; ED - Emergency Department; EHP - Environmental Health Practitioners; GBH – Grievous bodily harm; GOVT – Govt; HAH – High Alcohol Hours (6pm Friday-6am Sunday) Fulde et al., 2015 calls it HAT; INCL. - Including; IRR – Incident Rate Ratio; ITS – Interrupted Time Series; ITT – Intention-to-treat; LED – Licensed Entertainment Precinct; MPA – Minimum purchase age; NEP – Night-time entertainment precinct; NTE – Night-time economy; NY – New York; NZ- New Zealand; PDA – Proximal displacement area; OVI – Operating a vehicle while impaired; RBL – Risk Based Licensing; RBS The Swedish Responsible Beverage Service Program; RSA Responsible Service of Alcohol; RTM – Risk Terrain Modelling; RTMDx – Risk Terrain Modelling Diagnostics; SARIMA - Autoregressive Integrated Moving Average; SALUTT - STAD - Stockholm prevents alcohol and drug problems; SALUTT sammen lager vi utelivet tryggere” (together we make night life safer); SNP – Safe Night Precincts; TAFV – Tackling Alcohol Fuelled Violence Policy; UK – United Kingdom; USA – United States of America

References

1. Measham FC. Drug safety testing, disposals and dealing in an English field: Exploring the operational and behavioural outcomes of the UK's first onsite 'drug checking' service. Int J Drug Policy. 2019;67:102-7.

2. Measham F, Turnbull G. Intentions, actions and outcomes: A follow up survey on harm reduction practices after using an English festival drug checking service. Int J Drug Policy. 2021;95:Article 103270.

3. Curtis A, Farmer C, Harries T, Mayshak R, Coomber K, Guadagno B, et al. Do patron bans act as a deterrent to future anti-social offending? An analysis of banning and offending data from Victoria, Australia. Policing Soc. 2022;32(2):234-47.

4. Grigg J, Barratt MJ, Lenton S. Drug detection dogs at Australian outdoor music festivals: Deterrent, detection and iatrogenic effects. Int J Drug Policy. 2018;60:89-95.

5. Hickey S, McIlwraith F, Bruno R, Matthews A, Alati R. Drug detection dogs in Australia: More bark than bite? Drug Alcohol Rev. 2012;31(6):778-83.

6. Malins P. Drug dog affects: Accounting for the broad social, emotional and health impacts of general drug detection dog operations in Australia. Int J Drug Policy. 2019;67:63-71.

7. Rowe SC, Wiggers J, Wolfenden L, Francis JL, Freund M. Evaluation of an educational policing strategy to reduce alcohol-related crime associated with licensed premises. Can J Public Health. 2012;103(7 Suppl 1):eS8-14.

8. Taylor N, Coomber K, Zahnow R, Ferris J, Mayshak R, Miller PG. The prospective impact of 10‐day patron bans on crime in Queensland's largest entertainment precincts. Drug Alcohol Rev. 2021;40(5):771-8.

9. Archer JRH, Beaumont PO, May D, Dargan PI, Wood DM. Clinical survey assessing the appropriate management of individuals with acute recreational drug toxicity at a large outdoor festival event. J Subst Use. 2012;17(4):356-62.

10. Dutch MJ, Austin KB. Hospital in the field: Prehospital management of GHB intoxication by medical assistance teams. Prehosp Disaster Med. 2012;27(5):463-7.

11. Friedman NMG, O'Connor EK, Munro T, Goroff D. Mass-gathering medical care provided by a collegiate-based first response service at an annual college music festival and campus-wide celebration. Prehosp Disaster Med. 2019;34(1):98-103.

12. Lund A, Turris SA. Mass-gathering medicine: Risks and patient presentations at a 2-day electronic dance music event. Prehosp Disaster Med. 2015;30(3):271-8.

13. Wood DM, Beaumont PO, May D, Dargan PI. Recreational drug use presentations during a large outdoor festival event: Reduction in hospital emergency department transfer where medical physicians are present. J Subst Use. 2010;15(6):434-41.

14. Carvalho MC, de Sousa MP, Frango P, Dias P, Carvalho J, Rodrigues M, et al. Crisis intervention related to the use of psychoactive substances in recreational settings--evaluating the Kosmicare Project at Boom Festival. Curr Drug Abuse Rev. 2014;7(2):81-100.

15. Doran CM, Wadds P, Shakeshaft A, Tran DA. Impact and return on investment of the Take Kare Safe Space program—a harm reduction strategy implemented in Sydney, Australia. Int J Environ Res Public Health. 2021;18(22):Article 12111.

16. Garius L, Ward B, Teague K, Tseloni A. Evaluating harm-reduction initiatives in a night-time economy and music festival context. Crime and Fear in Public Places: Routledge; 2020. p. 362-78.

17. Taylor N, Coomber K, Curtis A, Mayshak R, Harries T, Ferris J, et al. The impact of street service care on frontline service utilisation during high‐alcohol use hours in one night‐time entertainment precinct in Australia. Drug Alcohol Rev. 2020;39(1):21-8.

18. Ward BM, O'Sullivan B, Buykx P. Evaluation of a local government "shelter and van" intervention to improve safety and reduce alcohol-related harm. BMC Public Health. 2018;18(1):Article 1370.

19. Curtis A, Droste N, Coomber K, Guadagno B, Mayshak R, Hyder S, et al. The impact of twenty four-hour public transport in Melbourne, Australia: An evaluation of alcohol-related harms. J Stud Alcohol Drugs. 2019;80(3):314-8.

20. Curtis A, Droste N, Coomber K, Guadagno B, Mayshak R, Hyder S, et al. Off the rails-Evaluating the nightlife impact of Melbourne, Australia's 24-h public transport trial. Int J Drug Policy. 2019;63:39-46.

21. Jackson CK, Owens EG. One for the road: Public transportation, alcohol consumption, and intoxicated driving. J Public Econ. 2011;95(1-2):106-21.

22. Kazbour RR, Bailey JS. An analysis of a contingency program on designated drivers at a college bar. J Appl Behav Anal. 2010;43(2):273-7.

23. Miller TR, Courser M, Shamblen SR, Lange JE, Tippetts AS, Ringwalt C. Efficacy and cost-effectiveness of subsidized ridesharing as a drunk driving intervention in Columbus, OH. Accid Anal Prev. 2020;146:Article 105740.

24. Rivara FP, Boisvert D, Relyea-Chew A, Gomez T. Last Call: Decreasing drunk driving among 21-34-year-old bar patrons. Int J Inj Contr Saf Promot. 2012;19(1):53-61.

25. Bassols NM, Castello JV. Bar opening hours, alcohol consumption and workplace accidents. Labour Econ. 2018;53:172-81.

26. Bernat DH, Maldonado-Molina M, Hyland A, Wagenaar AC. Effects of smoke-free laws on alcohol-related car crashes in California and New York: Time series analyses from 1982 to 2008. Am J Public Health. 2013;103(2):214-20.

27. Briggs S, Petrov A, Peterson S. Unanticipated consequences: The impact of a smoke-free law on assaults around bars. Criminal Justice Review. 2014;39(3):272-89.

28. Brown R, Evans E. Four years after the Licensing Act 2003: A case study of Hartlepool town centre. Safer Communities. 2011;10(1):39-46.

29. Burgason KA, Drawve G, Brown TC, Eassey J. Close only counts in alcohol and violence: Controlling violence near late night alcohol establishments using a routine activities approach. J Crim Justice. 2017;50:62-8.

30. Curtis A, Bowe SJ, Coomber K, Graham K, Chikritzhs T, Kypri K, et al. Risk-based licensing of alcohol venues and emergency department injury presentations in two Australian states. Int J Drug Policy. 2019;70:99-106.

31. de Andrade D, Homel R, Townsley M. Trouble in paradise: The crime and health outcomes of the Surfers Paradise licensed venue lockout. Drug Alcohol Rev. 2016;35(5):564-72.

32. de Goeij MC, Veldhuizen EM, Buster MC, Kunst AE. The impact of extended closing times of alcohol outlets on alcohol‐related injuries in the nightlife areas of Amsterdam: A controlled before‐and‐after evaluation. Addiction. 2015;110(6):955-64.

33. Forsyth AJM. The impact of the Scottish ban on smoking in public places upon nightclubs and their patrons. J Subst Use. 2012;17(3):203-17.

34. Green CP, Heywood JS, Navarro M. Did liberalising bar hours decrease traffic accidents? J Health Econ. 2014;35:189-98.

35. Gruenewald PJ, Treno AJ, Ponicki WR, Huckle T, Yeh LC, Casswell S. Impacts of New Zealand's lowered minimum purchase age on context-specific drinking and related risks. Addiction. 2015;110(11):1757-66.

36. Humphreys DK, Eisner MP, Wiebe DJ. Evaluating the impact of flexible alcohol trading hours on violence: An interrupted time series analysis. PLoS One. 2013;8(2):e55581.

37. Humphreys DK, Eisner MP. Do flexible alcohol trading hours reduce violence? A theory-based natural experiment in alcohol policy. Soc Sci Med. 2014;102:1-9.

38. Khurana S, Mahajan K. Public safety for women: Is regulation of social drinking spaces effective? J Dev Stud. 2022;58(1):164-82.

39. Kirby S, Hewitt L. The impact of the Licensing Act 2003 on drinking habits, offences of crime and disorder, and policing in England's newest city. Safer Communities. 2011;10(1):31-8.

40. Klein EG, Forster JL, Toomey TL, Broder-Oldach B, Erickson DJ, Collins NM. Did a local clean indoor air policy increase alcohol-related crime around bars and restaurants? Tob Control. 2013;22(2):113-7.

41. Mazerolle L, White G, Ransley J, Ferguson P. Violence in and around entertainment districts: A longitudinal analysis of the impact of late-night lockout legislation. Law & Policy. 2012;34(1):55-79.

42. Miller P, Coomber K, Sonderlund A, McKenzie S. The long-term effect of lockouts on alcohol-related emergency department attendances within Ballarat, Australia. Drug Alcohol Rev. 2012;31(4):370-6.

43. Miller PG, Curtis A, Graham K, Kypri K, Hudson K, Chikritzhs T. Understanding risk-based licensing schemes for alcohol outlets: A key informant perspective. Drug Alcohol Rev. 2020;39(3):267-77.

44. Moore SC, Murphy S, Moore SN, Brennan I, Byrne E, Shepherd J, et al. An exploratory randomised controlled trial of a premises-level intervention to reduce alcohol-related harm including violence in the United Kingdom. BMC Public Health. 2012;12:Article 412.

45. Nepal S, Kypri K, Attia J, Evans TJ, Chikritzhs T, Miller P. Effects of a risk-based licensing scheme on the incidence of alcohol-related assault in Queensland, Australia: A quasi-experimental evaluation. Int J Environ Res Public Health. 2019;16(23):Article 4637.

46. Palk GRM, Davey JD, Freeman JE. The impact of a lockout policy on levels of alcohol-related incidents in and around licensed premises. Police Pract Res. 2010;11(1):5-15.

47. Palk G, Davey J, Freeman J, Morgan H. Perspectives on the effectiveness of the late night liquor trading lockout legislative provision. Crim Justice Policy Rev. 2012;23(4):465-92.

48. Ragnarsdóttir T, Kjartansdóttir Á, Kristinsdóttir I, Theódórsdóttir S, Kristjánsson M, Davídsdóttir S. Alcohol-related mishaps on weekends in Reykjavík. Nordisk Alkohol Nark. 2011;28(1):83-96.

49. Randerson S, Casswell S, Huckle T. Changes in New Zealand's alcohol environment following implementation of the Sale and Supply of Alcohol Act (2012). N Z Med J. 2018;131(1476):14-23.

50. Rossow I, Norstrom T. The impact of small changes in bar closing hours on violence. The Norwegian experience from 18 cities. Addiction. 2012;107(3):530-7.

51. Taylor N, Coomber K, Mayshak R, Zahnow R, Ferris J, Miller P. The impact of liquor restrictions on serious assaults across Queensland, Australia. Int J Environ Res Public Health. 2019;16(22):Article 4362.

52. Tesch F, Hohendorf L. Do changes in bar opening hours influence violence in the night? Evidence from 13 Bavarian towns. J Drug Issues. 2018;48(2):295-306.

53. Tomedi LE, Roeber J, Xuan Z, Kanny D, Brewer RD, Naimi TS. Enhanced enforcement of laws to reduce alcohol overservice among licensed establishments in new mexico, 2004-2008. Prev Chronic Dis. 2018;15:E151.

54. Young-Wolff KC, Hyland AJ, Desai R, Sindelar J, Pilver CE, McKee SA. Smoke-free policies in drinking venues predict transitions in alcohol use disorders in a longitudinal U.S. sample. Drug Alcohol Depend. 2013;128(3):214-21.

55. Athanasopoulos G, Sarafidis V, Weatherburn D, Miller R. Longer-term impacts of trading restrictions on alcohol-related violence: Insights from New South Wales, Australia. Addiction. 2022;117(5):1304-11.

56. Brannstrom L, Trolldal B, Menke M. Spatial spillover effects of a community action programme targeting on-licensed premises on violent assaults: Evidence from a natural experiment. J Epidemiol Community Health. 2016;70(3):226-30.

57. Chamlin MB, Scott SE. Extending the hours of operation of alcohol serving establishments: An assessment of an innovative strategy to reduce the problems arising from the after-hours consumption of alcohol. Crim Justice Policy Rev. 2014;25(4):432-49.

58. Coomber K, de Andrade D, Puljević C, Ferris J, Livingston M, Taylor N, et al. The impact of liquor legislation changes on police‐recorded serious assault in Queensland, Australia. Drug Alcohol Rev. 2021;40(5):717-27.

59. Curtis A, Coomber K, Droste N, Hyder S, Palmer D, Miller PG. Effectiveness of community-based interventions for reducing alcohol-related harm in two metropolitan and two regional sites in Victoria, Australia. Drug Alcohol Rev. 2017;36(3):359-68.

60. de Andrade D, Coomber K, Livingston M, Taylor N, Moayeri F, Miller PG, et al. The impact of late‐night alcohol restrictions on ambulance call‐outs in entertainment precincts. Drug Alcohol Rev. 2021;40(5):708-16.

61. de Vocht F, Heron J, Angus C, Brennan A, Mooney J, Lock K, et al. Measurable effects of local alcohol licensing policies on population health in England. J Epidemiol Community Health. 2016;70(3):231-7.

62. de Vocht F, Heron J, Campbell R, Egan M, Mooney JD, Angus C, et al. Testing the impact of local alcohol licencing policies on reported crime rates in England. J Epidemiol Community Health. 2017;71(2):137-45.

63. de Vocht F, Tilling K, Pliakas T, Angus C, Egan M, Brennan A, et al. The intervention effect of local alcohol licensing policies on hospital admission and crime: A natural experiment using a novel Bayesian synthetictime-series method. J Epidemiol Community Health. 2017;71(9):912-8.

64. de Vocht F, McQuire C, Brennan A, Egan M, Angus C, Kaner E, et al. Evaluating the causal impact of individual alcohol licensing decisions on local health and crime using natural experiments with synthetic controls. Addiction. 2020;115(11):2021-31.

65. Devilly GJ, Hides L, Kavanagh DJ. A big night out getting bigger: Alcohol consumption, arrests and crowd numbers, before and after legislative change. PLoS One. 2019;14(6):e0218161.

66. Donnelly N, Poynton S, Weatherburn D. The effect of lockout and last drinks laws on non-domestic assaults in Sydney: An update to September 2016. Crime & Justice Bulletin. 2017;201:1-12.

67. Fell JC, Fisher DA, Yao J, McKnight AS. Evaluation of a responsible beverage service and enforcement program: Effects on bar patron intoxication and potential impaired driving by young adults. Traffic Inj Prev. 2017;18(6):557-65.

68. Ford K, Foulds J, Coleman O, Ardagh M, Pearson S, Droste N, et al. Alcohol-related emergency department attendances after the introduction of the Sale and Supply of Alcohol Act 2012. N Z Med J. 2018;131(1483):40-9.

69. Fulde GWO, Smith M, Forster SL. Presentations with alcohol-related serious injury to a major Sydney trauma hospital after 2014 changes to liquor laws. Med J Aust. 2015;203(9):Article 366.

70. George MD, Bodiford A, Humphries C, Stoneburner KA, Holder HD. Media and education effect on impaired driving associated with alcohol service. J Drug Educ. 2018;48(3-4):86-102.

71. Hoffman GR, Palazzi K, Boateng BKO, Oldmeadow C. Liquor legislation, last drinks, and lockouts: The Newcastle (Australia) solution. Int J Oral Maxillofac Surg. 2017;46(6):740-5.

72. Hughes CE, Weedon-Newstead AS. Investigating displacement effects as a result of the Sydney, NSW alcohol lockout legislation. Drugs Ed Prev Policy. 2018;25(5):386-96.

73. Kypri K, Jones C, McElduff P, Barker D. Effects of restricting pub closing times on night-time assaults in an Australian city. Addiction. 2011;106(2):303-10.

74. Kypri K, McElduff P, Miller P. Restrictions in pub closing times and lockouts in Newcastle, Australia five years on. Drug Alcohol Rev. 2014;33(3):323-6.

75. Kypri K, Livingston M. Incidence of assault in Sydney, Australia, throughout 5 years of alcohol trading hour restrictions: Controlled before‐and‐after study. Addiction. 2020;115(11):2045-54.

76. Livingston M, Coomber K, de Andrade D, Taylor N, Ferris J, Puljević C, et al. Assessing the impact of Queensland's late‐night alcohol restrictions using health system data. Drug Alcohol Rev. 2021;40(5):698-707.

77. Menéndez P, Weatherburn D, Kypri K, Fitzgerald J. Lockouts and last drinks: The impact of the January 2014 liquor licence reforms on assaults in NSW, Australia. Crime & Justice Bulletin. 2015;183:1-12.

78. Menendez P, Tusell F, Weatherburn D. The effects of liquor licensing restriction on alcohol-related violence in NSW, 2008-13. Addiction. 2015;110(10):1574-82.

79. Menendez P, Kypri K, Weatherburn D. The effect of liquor licensing restrictions on assault: A quasi-experimental study in Sydney, Australia. Addiction. 2017;112(2):261-8.

80. Miller P, Sonderlund A, Coomber K, Palmer D, Gillham K, Tindall J, et al. Do community interventions targeting licensed venues reduce alcohol-related emergency department presentations? Drug Alcohol Rev. 2011;30(5):546-53.

81. Miller P, Curtis A, Palmer D, Busija L, Tindall J, Droste N, et al. Changes in injury-related hospital emergency department presentations associated with the imposition of regulatory versus voluntary licensing conditions on licensed venues in two cities. Drug Alcohol Rev. 2014;33(3):314-22.

82. Munn MB, Lund A, Golby R, Turris SA. Observed benefits to on-site medical services during an annual 5-day electronic dance music event with harm reduction services. Prehosp Disaster Med. 2016;31(2):228-34.

83. Navarro HJ, Shakeshaft A, Doran CM, Petrie DJ. Does increasing community and liquor licensees' awareness, police activity, and feedback reduce alcohol-related violent crime? A benefit-cost analysis. Int J Environ Res Public Health. 2013;10(11):5490-506.

84. Norstrom T, Trolldal B. Was the STAD programme really that successful? Nordisk Alkohol Nark. 2013;30(3):171-8.

85. Norström T, Ramstedt M, Svensson J. Extended opening hours at nightclubs in Visby: An evaluation of a trial in the summer of 2014. Nordisk Alkohol Nark. 2018;35(5):388-96.

86. Paschall MJ, Miller TR, Grube JW, Fisher DA, Ringwalt CL, Kaner E, et al. Compliance with a law to reduce alcoholic beverage sales and service in Zacatecas, Mexico. Int J Drug Policy. 2021;97:Article 103352.

87. Pliakas T, Egan M, Gibbons J, Ashton C, Hart J, Lock K. Increasing powers to reject licences to sell alcohol: Impacts on availability, sales and behavioural outcomes from a novel natural experiment evaluation. Prev Med. 2018;116:87-93.

88. Skardhamar T, Fekjaer SB, Pedersen W. If it works there, will it work here? The effect of a multi-component responsible beverage service (RBS) programme on violence in Oslo. Drug Alcohol Depend. 2016;169:128-33.

89. Taylor N, Livingston M, Coomber K, Mayshak R, Zahnow R, Ferris J, et al. The combined impact of higher-risk on-license venue outlet density and trading hours on serious assaults in night-time entertainment precincts. Drug Alcohol Depend. 2021;223:Article 108720.

90. Trolldal B, Brannstrom L, Paschall MJ, Leifman H. Effects of a multi-component responsible beverage service programme on violent assaults in Sweden. Addiction. 2013;108(1):89-96.

91. Wiggers J, Tindall J, Hodder RK, Gillham K, Kingsland M, Lecathelinais C. Public opinion and experiences of crime two and five years following the implementation of a targeted regulation of licensed premises in Newcastle, Australia. Drug Alcohol Rev. 2021;40(3):489-98.

92. Xu Y, Yu Q, Scribner R, Theall K, Scribner S, Simonsen N. Multilevel spatiotemporal change-point models for evaluating the effect of an alcohol outlet control policy on changes in neighborhood assaultive violence rates. Spat Spatiotemporal Epidemiol. 2012;3(2):121-8.

93. Zhang X, Hatcher B, Clarkson L, Holt J, Bagchi S, Kanny D, et al. Changes in density of on-premises alcohol outlets and impact on violent crime, Atlanta, Georgia, 1997-2007. Prev Chronic Dis. 2015;12:E84.

94. Charlebois E, Plenty A, Lin J, Ayala A, Hecht J. Impact of a structural intervention to address alcohol use among gay bar patrons in San Francisco: The PACE study. AIDS Behav. 2017;21:193-202.

95. Farrimond H, Boyd K, Fleischer D. Reconfiguring the violent encounter? Preloading, security staff and breathalyser use in the night-time economy. Int J Drug Policy. 2018;56:108-15.

96. Ham A, Maldonado D, Weintraub M, Camacho AF, Gualtero D. Reducing alcohol‐related violence with bartenders: A behavioral field experiment. J Policy Anal Manage. 2022;41(3):731-61.

97. Moore SC, Alam MF, Heikkinen M, Hood K, Huang C, Moore L, et al. The effectiveness of an intervention to reduce alcohol-related violence in premises licensed for the sale and on-site consumption of alcohol: A randomized controlled trial. Addiction. 2017;112(11):1898-906.

98. Zawisza TT, Burgason KA. A donnybrook in downtown? Observations of controlling aggression and the use of effective place management in a southern entertainment area. Crime Prev Community Saf. 2020;22(1):1-16.

99. Baldin YC, Sanudo A, Sanchez ZM. Effectiveness of a web-based intervention in reducing binge drinking among nightclub patrons. Revista de saude publica. 2018;52:Article 2.

100. Monezi Andrade AL, Scatena A, De Micheli D. Evaluation of a preventive intervention in alcoholic and non-alcoholic drivers - A pilot study. SMAD: Revista Eletrônica Saúde Mental Álcool e Drogas. 2017;13(4):205-12.
