# Supplementary material for: A systematic review of interventions that impact alcohol and other drug-related harms in licensed entertainment settings and outdoor music festivals
Source: Harm Reduct J. 2024 Feb 21;21:47. doi: 10.1186/s12954-024-00949-4 (PMC10882826; doi:10.1186/s12954-024-00949-4)
Supplement: Supplementary file 6 — Additional file 6a: Health Outcome Effects. 6b: Criminal Justice Outcome Effects. 6c: Behavioural Outcome Effects. [file 12954_2024_949_MOESM6_ESM.docx]

**Additional File 6a. Health Outcome Effects**

| Author(s) | **Hospitalisations** | **Injuries** | **Road Traffic Accidents** | **Ambulance attendance** | **Mental Health** | **Death** | **Other** |
| --- | --- | --- | --- | --- | --- | --- | --- |
| *Laws and regulations* | | | | | | | |
| Bassols 2018 (1) | **+** | **+** |  |  |  |  |  |
| Bernat 2013 (2) |  |  | **=** |  |  | **=** |  |
| Brown 2011 (3) |  |  |  | **-** |  |  |  |
| Curtis 2019 (4) | **=** |  |  |  |  |  |  |
| de Andrade 2016 (5) |  |  |  | **=** |  |  |  |
| de Goeij 2015 (6) |  |  |  | **-** |  |  |  |
| Green 2014 (7) |  |  | **+** |  |  |  |  |
| Miller 2012 (8) | **=** |  |  |  |  |  |  |
| Ragnarsdóttir 2011 (9) | **-** |  |  |  |  |  |  |
| Young-Wolff 2013 (10) |  |  |  |  |  |  | **+** |
| *Multicomponent intervention* | | | | | | | |
| Curtis 2017 (11) | **=** |  |  |  |  |  |  |
| de Andrade 2021 (12) |  |  |  | **+** |  |  |  |
| de Vocht 2016 (13) | **+** |  |  |  |  |  |  |
| de Vocht 2017 (14) | **+** |  |  |  |  |  |  |
| de Vocht 2020 (15) | **=** |  |  | **=** |  |  |  |
| Fell 2017 (16) |  |  | **=** |  |  |  |  |
| Ford 2018 (17) | **=** |  |  |  |  |  |  |
| Fulde 2015 (18) | **+** |  |  |  |  |  |  |
| Hoffman 2017 (19) | **+** |  |  |  |  |  |  |
| Livingston 2021 (20) | **+** |  |  |  |  |  |  |
| Miller 2011 (21) | **-** |  |  |  |  |  |  |
| Miller 2014 (22) | **+** |  |  |  |  |  |  |
| Munn 2016 (23) |  |  |  | **+** |  |  |  |
| Paschall 2021 (24) |  | **+/=** |  |  |  |  |  |
| Pliakas 2018 (25) |  |  |  | **=** |  |  |  |
| Rivara 2012 (26) |  |  | **+** |  |  |  |  |
| *Drug checking/pill testing* | | | | | | | |
| Measham 2019 (27) | + |  |  |  |  |  |  |
| *Transport Intervention* | | | | | | | |
| Curtis 2019 (28) |  |  | +/- | = |  |  |  |
| Curtis 2019 (29) |  | = |  |  |  |  |  |
| Miller T 2020 (30) |  |  | = |  |  |  |  |
| *Venue licensing policy* | | | | | | | |
| Miller P 2020 (31) |  |  |  |  |  |  | = |
| *Medical service* | | | | | | | |
| Archer 2012 (32) |  |  |  | + |  |  |  |
| Dutch 2012 (33) | + |  |  | + |  |  |  |
| Friedman 2019 (34) |  |  |  | + |  |  |  |
| Lund 2015 (35) |  |  |  | + |  |  |  |
| Wood 2010 (36) | + |  |  |  |  |  |  |
| *Chill/safe space and roaming support services* | | | | | | | |
| Carvalho 2014 (37) |  |  |  |  | + |  |  |
| Doran 2021 (38) |  |  | + |  |  |  |  |
| Taylor 2020 (39) | = |  |  | = |  |  |  |
| Ward 2018 (40) | = |  |  |  |  |  |  |

**Additional File 6b. Criminal Justice Outcome Effects**

| Author(s) | Assault (excl sexual assault) | Violence^1^ | Driving Offences | Sexual assault and related offences | General Crime | Public order offence | Liquor Offenses | Drug Offenses | Other Offences |
| --- | --- | --- | --- | --- | --- | --- | --- | --- | --- |
| *Laws and regulations* | | | | | | | | | |
| Briggs 2014 (41) | + |  |  |  |  |  |  |  |  |
| Brown 2011 (3) | + |  |  |  |  | +/- |  |  | - |
| de Andrade 2016 (5) |  | = |  |  | = |  |  |  |  |
| Forsyth 2012 (42) |  | +/- |  | - |  |  |  |  |  |
| Humphreys 2013 (43) | = |  |  |  |  |  |  |  |  |
| Humphreys 2014 (44) | = |  |  |  |  |  |  |  |  |
| Khurana 2022 (45) |  | = |  | + |  |  |  |  |  |
| Kirby 2011 (46) |  |  |  |  | + |  |  |  |  |
| Klein 2013 (47) | = |  |  | = | = | = | = |  |  |
| Mazerolle 2012 (48) | + |  |  |  |  |  |  |  |  |
| Palk 2010 (49) | = |  | = | + | = | + | + |  |  |
| Palk 2012 (50) |  |  | + | + | = | + |  |  | + |
| Ragnarsdóttir 2011 (9) | - |  |  |  |  |  |  |  |  |
| Rossow 2012 (51) | - |  |  |  |  |  |  |  |  |
| Taylor 2019 (52) | = |  |  |  |  |  |  |  |  |
| Tesch 2018 (53) |  | = |  |  |  |  |  |  |  |
| *Staff and venue intervention* | | | | | | | | | |
| Ham 2022 (54) | = |  |  |  |  | + |  |  | = |
| Moore 2017 (55) | - |  |  |  |  |  |  |  |  |
| *Patron Survey and Assessment Feedback* | | | | | | | | | |
| Monezi 2017 (56) |  |  | = |  |  |  |  |  |  |
| *Multicomponent intervention* | | | | | | | | | |
| Athanasopoulos 2022 (57) | + |  |  |  |  |  |  |  |  |
| Brännström 2016 (58) | + |  |  |  |  |  |  |  |  |
| Burgason 2017 (59) |  | + |  |  |  |  |  |  |  |
| Chamlin 2014 (60) |  |  |  |  |  | = |  |  |  |
| Coomber 2021 (61) | + |  |  |  |  |  |  |  |  |
| Curtis 2017 (11) | = |  |  |  |  |  |  |  |  |
| de Vocht 2017 (62) | + |  |  | + |  | + |  |  |  |
| de Vocht 2017 (14) | + | + |  | + |  | = |  |  |  |
| de Vocht 2020 (15) | = |  |  | = | = | + |  |  | + |
| Devilly 2019 (63) | = |  |  |  |  | = |  | - |  |
| Donnelly 2017 (64) | + |  |  |  |  |  |  |  |  |
| Fell 2017 (16) |  |  | + |  |  |  |  |  |  |
| George 2018 (65) |  |  | + |  |  |  |  |  |  |
| Kypri 2011 (66) | + |  |  |  |  |  |  |  |  |
| Kypri 2014 (67) | + |  |  |  |  |  |  |  |  |
| Kypri 2020 (68) | + |  |  |  |  |  |  |  |  |
| Menéndez 2015 (69) | + |  |  |  |  |  |  |  |  |
| Menéndez 2015 (70) | + |  |  |  |  |  |  |  |  |
| Menéndez 2017 (71) | + |  |  |  |  |  |  |  |  |
| Navarro 2013 (72) | = |  |  | + |  |  |  |  |  |
| Norström 2013 (73) | + |  |  |  |  |  |  |  |  |
| Norström 2018 (74) | + |  |  |  |  |  |  |  |  |
| Paschall 2021 (24) |  | +/= |  |  |  |  |  |  |  |
| Pliakas 2018 (25) |  |  |  |  | + | +/- |  |  |  |
| Randerson 2018 (75) |  |  | + |  |  |  |  |  |  |
| Skardhamar 2016 (76) |  | = |  |  |  |  |  |  |  |
| Taylor 2021 (77) | + |  |  |  |  |  |  |  |  |
| Trolldal 2013 (78) | + |  |  |  |  |  |  |  |  |
| Wiggers 2021 (79) |  | + |  |  | + |  |  |  |  |
| Xu 2012 (80) | = |  |  |  |  |  |  |  |  |
| Zhang 2015 (81) |  | + |  |  |  |  |  |  |  |
| *Transport intervention* | | | | | | | | | |
| Curtis 2019 (28) | - |  |  |  |  |  |  |  |  |
| Jackson 2011 (82) |  |  | = |  |  |  | = |  |  |
| Kazbour 2010 (83) |  |  | + |  |  |  |  |  |  |
| Miller T 2020 (30) |  |  | = |  |  |  |  |  |  |
| *Venue licensing policy* | | | | | | | | | |
| Moore 2012 (84) | = |  |  |  |  |  |  |  |  |
| Nepal 2019 (85) | = |  |  |  |  |  |  |  |  |
| *Policing strategy* | | | | | | | | | |
| Curtis 2022 (86) | = | = |  |  | = | - |  | = | = |
| Hickey 2012 (87) |  |  |  |  |  |  |  | = | = |
| Rowe 2012 (88) |  |  |  |  |  |  |  |  | + |
| Taylor 2021 (89) | = |  |  |  |  | = |  |  |  |
| *Chill/safe space and roaming support services* | | | |  |  |  |  |  |  |
| Doran 2021 (38) | + |  |  | + |  |  |  |  | + |
| Garius 2020 (90) | - |  |  | - |  |  |  |  |  |
| Taylor 2020 (39) | +/- |  |  |  |  |  |  |  |  |
| Ward 2018 (40) | = | - |  |  | = | = |  |  |  |

*Notes:* ^1^ May (not) meet the legal threshold of assault

**Additional File 6c. Behavioural Outcome Effects**

| Author(s) | Risky Consumption Practices | Overcrowding | Aggression | Using Public Transport/Ride-hailing |
| --- | --- | --- | --- | --- |
| *Drug checking / Pill testing* | | | | |
| Measham 2021 (91) | + |  |  |  |
| *Laws and regulations* | | | | |
| Forsyth 2012 (42) | - | + |  |  |
| Gruenewald 2015 (92) | - |  |  |  |
| Hughes 2018 (93) |  |  | + |  |
| Palk 2012 (50) |  | - |  |  |
| Tomedi 2018 (94) | + |  |  |  |
| *Multicomponent intervention* | | | | |
| Chamlin 2014 (60) |  |  |  | - |
| Fell 2017 (16) | - |  |  |  |
| Wiggers 2021 (79) | + |  |  |  |
| *Policing strategy* | | | | |
| Farrimond 2018 (95) |  |  | +/- |  |
| Grigg 2018 (96) | - |  |  |  |
| Hickey 2012 (87) | - |  |  |  |
| Malins 2019 (97) | - |  |  |  |
| *Patron Survey and Assessment Feedback* | | | | |
| Baldin 2018 (98) | + |  |  |  |
| *Staff and venue intervention* | | | | |
| Charlebois 2017 (99) | + |  |  |  |
| Zawisza 2020 (100) |  |  | + |  |
| *Transport Intervention* | | | | |
| Curtis 2019 (29) |  |  | - | + |
| Miller T 2020 (30) |  |  |  | + |

References

1. Bassols NM, Castello JV. Bar opening hours, alcohol consumption and workplace accidents. Labour Econ. 2018;53:172-81.

2. Bernat DH, Maldonado-Molina M, Hyland A, Wagenaar AC. Effects of smoke-free laws on alcohol-related car crashes in California and New York: Time series analyses from 1982 to 2008. Am J Public Health. 2013;103(2):214-20.

3. Brown R, Evans E. Four years after the Licensing Act 2003: A case study of Hartlepool town centre. Safer Communities. 2011;10(1):39-46.

4. Curtis A, Bowe SJ, Coomber K, Graham K, Chikritzhs T, Kypri K, et al. Risk-based licensing of alcohol venues and emergency department injury presentations in two Australian states. Int J Drug Policy. 2019;70:99-106.

5. de Andrade D, Homel R, Townsley M. Trouble in paradise: The crime and health outcomes of the Surfers Paradise licensed venue lockout. Drug Alcohol Rev. 2016;35(5):564-72.

6. de Goeij MC, Veldhuizen EM, Buster MC, Kunst AE. The impact of extended closing times of alcohol outlets on alcohol‐related injuries in the nightlife areas of Amsterdam: A controlled before‐and‐after evaluation. Addiction. 2015;110(6):955-64.

7. Green CP, Heywood JS, Navarro M. Did liberalising bar hours decrease traffic accidents? J Health Econ. 2014;35:189-98.

8. Miller P, Coomber K, Sonderlund A, McKenzie S. The long-term effect of lockouts on alcohol-related emergency department attendances within Ballarat, Australia. Drug Alcohol Rev. 2012;31(4):370-6.

9. Ragnarsdóttir T, Kjartansdóttir Á, Kristinsdóttir I, Theódórsdóttir S, Kristjánsson M, Davídsdóttir S. Alcohol-related mishaps on weekends in Reykjavík. Nordisk Alkohol Nark. 2011;28(1):83-96.

10. Young-Wolff KC, Hyland AJ, Desai R, Sindelar J, Pilver CE, McKee SA. Smoke-free policies in drinking venues predict transitions in alcohol use disorders in a longitudinal U.S. sample. Drug Alcohol Depend. 2013;128(3):214-21.

11. Curtis A, Coomber K, Droste N, Hyder S, Palmer D, Miller PG. Effectiveness of community-based interventions for reducing alcohol-related harm in two metropolitan and two regional sites in Victoria, Australia. Drug Alcohol Rev. 2017;36(3):359-68.

12. de Andrade D, Coomber K, Livingston M, Taylor N, Moayeri F, Miller PG, et al. The impact of late‐night alcohol restrictions on ambulance call‐outs in entertainment precincts. Drug Alcohol Rev. 2021;40(5):708-16.

13. de Vocht F, Heron J, Angus C, Brennan A, Mooney J, Lock K, et al. Measurable effects of local alcohol licensing policies on population health in England. J Epidemiol Community Health. 2016;70(3):231-7.

14. de Vocht F, Tilling K, Pliakas T, Angus C, Egan M, Brennan A, et al. The intervention effect of local alcohol licensing policies on hospital admission and crime: A natural experiment using a novel Bayesian synthetictime-series method. J Epidemiol Community Health. 2017;71(9):912-8.

15. de Vocht F, McQuire C, Brennan A, Egan M, Angus C, Kaner E, et al. Evaluating the causal impact of individual alcohol licensing decisions on local health and crime using natural experiments with synthetic controls. Addiction. 2020;115(11):2021-31.

16. Fell JC, Fisher DA, Yao J, McKnight AS. Evaluation of a responsible beverage service and enforcement program: Effects on bar patron intoxication and potential impaired driving by young adults. Traffic Inj Prev. 2017;18(6):557-65.

17. Ford K, Foulds J, Coleman O, Ardagh M, Pearson S, Droste N, et al. Alcohol-related emergency department attendances after the introduction of the Sale and Supply of Alcohol Act 2012. N Z Med J. 2018;131(1483):40-9.

18. Fulde GWO, Smith M, Forster SL. Presentations with alcohol-related serious injury to a major Sydney trauma hospital after 2014 changes to liquor laws. Med J Aust. 2015;203(9):Article 366.

19. Hoffman GR, Palazzi K, Boateng BKO, Oldmeadow C. Liquor legislation, last drinks, and lockouts: The Newcastle (Australia) solution. Int J Oral Maxillofac Surg. 2017;46(6):740-5.

20. Livingston M, Coomber K, de Andrade D, Taylor N, Ferris J, Puljević C, et al. Assessing the impact of Queensland's late‐night alcohol restrictions using health system data. Drug Alcohol Rev. 2021;40(5):698-707.

21. Miller P, Sonderlund A, Coomber K, Palmer D, Gillham K, Tindall J, et al. Do community interventions targeting licensed venues reduce alcohol-related emergency department presentations? Drug Alcohol Rev. 2011;30(5):546-53.

22. Miller P, Curtis A, Palmer D, Busija L, Tindall J, Droste N, et al. Changes in injury-related hospital emergency department presentations associated with the imposition of regulatory versus voluntary licensing conditions on licensed venues in two cities. Drug Alcohol Rev. 2014;33(3):314-22.

23. Munn MB, Lund A, Golby R, Turris SA. Observed benefits to on-site medical services during an annual 5-day electronic dance music event with harm reduction services. Prehosp Disaster Med. 2016;31(2):228-34.

24. Paschall MJ, Miller TR, Grube JW, Fisher DA, Ringwalt CL, Kaner E, et al. Compliance with a law to reduce alcoholic beverage sales and service in Zacatecas, Mexico. Int J Drug Policy. 2021;97:Article 103352.

25. Pliakas T, Egan M, Gibbons J, Ashton C, Hart J, Lock K. Increasing powers to reject licences to sell alcohol: Impacts on availability, sales and behavioural outcomes from a novel natural experiment evaluation. Prev Med. 2018;116:87-93.

26. Rivara FP, Boisvert D, Relyea-Chew A, Gomez T. Last Call: Decreasing drunk driving among 21-34-year-old bar patrons. Int J Inj Contr Saf Promot. 2012;19(1):53-61.

27. Measham FC. Drug safety testing, disposals and dealing in an English field: Exploring the operational and behavioural outcomes of the UK's first onsite 'drug checking' service. Int J Drug Policy. 2019;67:102-7.

28. Curtis A, Droste N, Coomber K, Guadagno B, Mayshak R, Hyder S, et al. The impact of twenty four-hour public transport in Melbourne, Australia: An evaluation of alcohol-related harms. J Stud Alcohol Drugs. 2019;80(3):314-8.

29. Curtis A, Droste N, Coomber K, Guadagno B, Mayshak R, Hyder S, et al. Off the rails-Evaluating the nightlife impact of Melbourne, Australia's 24-h public transport trial. Int J Drug Policy. 2019;63:39-46.

30. Miller TR, Courser M, Shamblen SR, Lange JE, Tippetts AS, Ringwalt C. Efficacy and cost-effectiveness of subsidized ridesharing as a drunk driving intervention in Columbus, OH. Accid Anal Prev. 2020;146:Article 105740.

31. Miller PG, Curtis A, Graham K, Kypri K, Hudson K, Chikritzhs T. Understanding risk-based licensing schemes for alcohol outlets: A key informant perspective. Drug Alcohol Rev. 2020;39(3):267-77.

32. Archer JRH, Beaumont PO, May D, Dargan PI, Wood DM. Clinical survey assessing the appropriate management of individuals with acute recreational drug toxicity at a large outdoor festival event. J Subst Use. 2012;17(4):356-62.

33. Dutch MJ, Austin KB. Hospital in the field: Prehospital management of GHB intoxication by medical assistance teams. Prehosp Disaster Med. 2012;27(5):463-7.

34. Friedman NMG, O'Connor EK, Munro T, Goroff D. Mass-gathering medical care provided by a collegiate-based first response service at an annual college music festival and campus-wide celebration. Prehosp Disaster Med. 2019;34(1):98-103.

35. Lund A, Turris SA. Mass-gathering medicine: Risks and patient presentations at a 2-day electronic dance music event. Prehosp Disaster Med. 2015;30(3):271-8.

36. Wood DM, Beaumont PO, May D, Dargan PI. Recreational drug use presentations during a large outdoor festival event: Reduction in hospital emergency department transfer where medical physicians are present. J Subst Use. 2010;15(6):434-41.

37. Carvalho MC, de Sousa MP, Frango P, Dias P, Carvalho J, Rodrigues M, et al. Crisis intervention related to the use of psychoactive substances in recreational settings--evaluating the Kosmicare Project at Boom Festival. Curr Drug Abuse Rev. 2014;7(2):81-100.

38. Doran CM, Wadds P, Shakeshaft A, Tran DA. Impact and return on investment of the Take Kare Safe Space program—a harm reduction strategy implemented in Sydney, Australia. Int J Environ Res Public Health. 2021;18(22):Article 12111.

39. Taylor N, Coomber K, Curtis A, Mayshak R, Harries T, Ferris J, et al. The impact of street service care on frontline service utilisation during high‐alcohol use hours in one night‐time entertainment precinct in Australia. Drug Alcohol Rev. 2020;39(1):21-8.

40. Ward BM, O'Sullivan B, Buykx P. Evaluation of a local government "shelter and van" intervention to improve safety and reduce alcohol-related harm. BMC Public Health. 2018;18(1):Article 1370.

41. Briggs S, Petrov A, Peterson S. Unanticipated consequences: The impact of a smoke-free law on assaults around bars. Criminal Justice Review. 2014;39(3):272-89.

42. Forsyth AJM. The impact of the Scottish ban on smoking in public places upon nightclubs and their patrons. J Subst Use. 2012;17(3):203-17.

43. Humphreys DK, Eisner MP, Wiebe DJ. Evaluating the impact of flexible alcohol trading hours on violence: An interrupted time series analysis. PLoS One. 2013;8(2):e55581.

44. Humphreys DK, Eisner MP. Do flexible alcohol trading hours reduce violence? A theory-based natural experiment in alcohol policy. Soc Sci Med. 2014;102:1-9.

45. Khurana S, Mahajan K. Public safety for women: Is regulation of social drinking spaces effective? J Dev Stud. 2022;58(1):164-82.

46. Kirby S, Hewitt L. The impact of the Licensing Act 2003 on drinking habits, offences of crime and disorder, and policing in England's newest city. Safer Communities. 2011;10(1):31-8.

47. Klein EG, Forster JL, Toomey TL, Broder-Oldach B, Erickson DJ, Collins NM. Did a local clean indoor air policy increase alcohol-related crime around bars and restaurants? Tob Control. 2013;22(2):113-7.

48. Mazerolle L, White G, Ransley J, Ferguson P. Violence in and around entertainment districts: A longitudinal analysis of the impact of late-night lockout legislation. Law & Policy. 2012;34(1):55-79.

49. Palk GRM, Davey JD, Freeman JE. The impact of a lockout policy on levels of alcohol-related incidents in and around licensed premises. Police Pract Res. 2010;11(1):5-15.

50. Palk G, Davey J, Freeman J, Morgan H. Perspectives on the effectiveness of the late night liquor trading lockout legislative provision. Crim Justice Policy Rev. 2012;23(4):465-92.

51. Rossow I, Norstrom T. The impact of small changes in bar closing hours on violence. The Norwegian experience from 18 cities. Addiction. 2012;107(3):530-7.

52. Taylor N, Coomber K, Mayshak R, Zahnow R, Ferris J, Miller P. The impact of liquor restrictions on serious assaults across Queensland, Australia. Int J Environ Res Public Health. 2019;16(22):Article 4362.

53. Tesch F, Hohendorf L. Do changes in bar opening hours influence violence in the night? Evidence from 13 Bavarian towns. J Drug Issues. 2018;48(2):295-306.

54. Ham A, Maldonado D, Weintraub M, Camacho AF, Gualtero D. Reducing alcohol‐related violence with bartenders: A behavioral field experiment. J Policy Anal Manage. 2022;41(3):731-61.

55. Moore SC, Alam MF, Heikkinen M, Hood K, Huang C, Moore L, et al. The effectiveness of an intervention to reduce alcohol-related violence in premises licensed for the sale and on-site consumption of alcohol: A randomized controlled trial. Addiction. 2017;112(11):1898-906.

56. Monezi Andrade AL, Scatena A, De Micheli D. Evaluation of a preventive intervention in alcoholic and non-alcoholic drivers - A pilot study. SMAD: Revista Eletrônica Saúde Mental Álcool e Drogas. 2017;13(4):205-12.

57. Athanasopoulos G, Sarafidis V, Weatherburn D, Miller R. Longer-term impacts of trading restrictions on alcohol-related violence: Insights from New South Wales, Australia. Addiction. 2022;117(5):1304-11.

58. Brannstrom L, Trolldal B, Menke M. Spatial spillover effects of a community action programme targeting on-licensed premises on violent assaults: Evidence from a natural experiment. J Epidemiol Community Health. 2016;70(3):226-30.

59. Burgason KA, Drawve G, Brown TC, Eassey J. Close only counts in alcohol and violence: Controlling violence near late night alcohol establishments using a routine activities approach. J Crim Justice. 2017;50:62-8.

60. Chamlin MB, Scott SE. Extending the hours of operation of alcohol serving establishments: An assessment of an innovative strategy to reduce the problems arising from the after-hours consumption of alcohol. Crim Justice Policy Rev. 2014;25(4):432-49.

61. Coomber K, de Andrade D, Puljević C, Ferris J, Livingston M, Taylor N, et al. The impact of liquor legislation changes on police‐recorded serious assault in Queensland, Australia. Drug Alcohol Rev. 2021;40(5):717-27.

62. de Vocht F, Heron J, Campbell R, Egan M, Mooney JD, Angus C, et al. Testing the impact of local alcohol licencing policies on reported crime rates in England. J Epidemiol Community Health. 2017;71(2):137-45.

63. Devilly GJ, Hides L, Kavanagh DJ. A big night out getting bigger: Alcohol consumption, arrests and crowd numbers, before and after legislative change. PLoS One. 2019;14(6):e0218161.

64. Donnelly N, Poynton S, Weatherburn D. The effect of lockout and last drinks laws on non-domestic assaults in Sydney: An update to September 2016. Crime & Justice Bulletin. 2017;201:1-12.

65. George MD, Bodiford A, Humphries C, Stoneburner KA, Holder HD. Media and education effect on impaired driving associated with alcohol service. J Drug Educ. 2018;48(3-4):86-102.

66. Kypri K, Jones C, McElduff P, Barker D. Effects of restricting pub closing times on night-time assaults in an Australian city. Addiction. 2011;106(2):303-10.

67. Kypri K, McElduff P, Miller P. Restrictions in pub closing times and lockouts in Newcastle, Australia five years on. Drug Alcohol Rev. 2014;33(3):323-6.

68. Kypri K, Livingston M. Incidence of assault in Sydney, Australia, throughout 5 years of alcohol trading hour restrictions: Controlled before‐and‐after study. Addiction. 2020;115(11):2045-54.

69. Menéndez P, Weatherburn D, Kypri K, Fitzgerald J. Lockouts and last drinks: The impact of the January 2014 liquor licence reforms on assaults in NSW, Australia. Crime & Justice Bulletin. 2015;183:1-12.

70. Menendez P, Tusell F, Weatherburn D. The effects of liquor licensing restriction on alcohol-related violence in NSW, 2008-13. Addiction. 2015;110(10):1574-82.

71. Menendez P, Kypri K, Weatherburn D. The effect of liquor licensing restrictions on assault: A quasi-experimental study in Sydney, Australia. Addiction. 2017;112(2):261-8.

72. Navarro HJ, Shakeshaft A, Doran CM, Petrie DJ. Does increasing community and liquor licensees' awareness, police activity, and feedback reduce alcohol-related violent crime? A benefit-cost analysis. Int J Environ Res Public Health. 2013;10(11):5490-506.

73. Norstrom T, Trolldal B. Was the STAD programme really that successful? Nordisk Alkohol Nark. 2013;30(3):171-8.

74. Norström T, Ramstedt M, Svensson J. Extended opening hours at nightclubs in Visby: An evaluation of a trial in the summer of 2014. Nordisk Alkohol Nark. 2018;35(5):388-96.

75. Randerson S, Casswell S, Huckle T. Changes in New Zealand's alcohol environment following implementation of the Sale and Supply of Alcohol Act (2012). N Z Med J. 2018;131(1476):14-23.

76. Skardhamar T, Fekjaer SB, Pedersen W. If it works there, will it work here? The effect of a multi-component responsible beverage service (RBS) programme on violence in Oslo. Drug Alcohol Depend. 2016;169:128-33.

77. Taylor N, Livingston M, Coomber K, Mayshak R, Zahnow R, Ferris J, et al. The combined impact of higher-risk on-license venue outlet density and trading hours on serious assaults in night-time entertainment precincts. Drug Alcohol Depend. 2021;223:Article 108720.

78. Trolldal B, Brannstrom L, Paschall MJ, Leifman H. Effects of a multi-component responsible beverage service programme on violent assaults in Sweden. Addiction. 2013;108(1):89-96.

79. Wiggers J, Tindall J, Hodder RK, Gillham K, Kingsland M, Lecathelinais C. Public opinion and experiences of crime two and five years following the implementation of a targeted regulation of licensed premises in Newcastle, Australia. Drug Alcohol Rev. 2021;40(3):489-98.

80. Xu Y, Yu Q, Scribner R, Theall K, Scribner S, Simonsen N. Multilevel spatiotemporal change-point models for evaluating the effect of an alcohol outlet control policy on changes in neighborhood assaultive violence rates. Spat Spatiotemporal Epidemiol. 2012;3(2):121-8.

81. Zhang X, Hatcher B, Clarkson L, Holt J, Bagchi S, Kanny D, et al. Changes in density of on-premises alcohol outlets and impact on violent crime, Atlanta, Georgia, 1997-2007. Prev Chronic Dis. 2015;12:E84.

82. Jackson CK, Owens EG. One for the road: Public transportation, alcohol consumption, and intoxicated driving. J Public Econ. 2011;95(1-2):106-21.

83. Kazbour RR, Bailey JS. An analysis of a contingency program on designated drivers at a college bar. J Appl Behav Anal. 2010;43(2):273-7.

84. Moore SC, Murphy S, Moore SN, Brennan I, Byrne E, Shepherd J, et al. An exploratory randomised controlled trial of a premises-level intervention to reduce alcohol-related harm including violence in the United Kingdom. BMC Public Health. 2012;12:Article 412.

85. Nepal S, Kypri K, Attia J, Evans TJ, Chikritzhs T, Miller P. Effects of a risk-based licensing scheme on the incidence of alcohol-related assault in Queensland, Australia: A quasi-experimental evaluation. Int J Environ Res Public Health. 2019;16(23):Article 4637.

86. Curtis A, Farmer C, Harries T, Mayshak R, Coomber K, Guadagno B, et al. Do patron bans act as a deterrent to future anti-social offending? An analysis of banning and offending data from Victoria, Australia. Policing Soc. 2022;32(2):234-47.

87. Hickey S, McIlwraith F, Bruno R, Matthews A, Alati R. Drug detection dogs in Australia: More bark than bite? Drug Alcohol Rev. 2012;31(6):778-83.

88. Rowe SC, Wiggers J, Wolfenden L, Francis JL, Freund M. Evaluation of an educational policing strategy to reduce alcohol-related crime associated with licensed premises. Can J Public Health. 2012;103(7 Suppl 1):eS8-14.

89. Taylor N, Coomber K, Zahnow R, Ferris J, Mayshak R, Miller PG. The prospective impact of 10‐day patron bans on crime in Queensland's largest entertainment precincts. Drug Alcohol Rev. 2021;40(5):771-8.

90. Garius L, Ward B, Teague K, Tseloni A. Evaluating harm-reduction initiatives in a night-time economy and music festival context. Crime and Fear in Public Places: Routledge; 2020. p. 362-78.

91. Measham F, Turnbull G. Intentions, actions and outcomes: A follow up survey on harm reduction practices after using an English festival drug checking service. Int J Drug Policy. 2021;95:Article 103270.

92. Gruenewald PJ, Treno AJ, Ponicki WR, Huckle T, Yeh LC, Casswell S. Impacts of New Zealand's lowered minimum purchase age on context-specific drinking and related risks. Addiction. 2015;110(11):1757-66.

93. Hughes CE, Weedon-Newstead AS. Investigating displacement effects as a result of the Sydney, NSW alcohol lockout legislation. Drugs Ed Prev Policy. 2018;25(5):386-96.

94. Tomedi LE, Roeber J, Xuan Z, Kanny D, Brewer RD, Naimi TS. Enhanced enforcement of laws to reduce alcohol overservice among licensed establishments in new mexico, 2004-2008. Prev Chronic Dis. 2018;15:E151.

95. Farrimond H, Boyd K, Fleischer D. Reconfiguring the violent encounter? Preloading, security staff and breathalyser use in the night-time economy. Int J Drug Policy. 2018;56:108-15.

96. Grigg J, Barratt MJ, Lenton S. Drug detection dogs at Australian outdoor music festivals: Deterrent, detection and iatrogenic effects. Int J Drug Policy. 2018;60:89-95.

97. Malins P. Drug dog affects: Accounting for the broad social, emotional and health impacts of general drug detection dog operations in Australia. Int J Drug Policy. 2019;67:63-71.

98. Baldin YC, Sanudo A, Sanchez ZM. Effectiveness of a web-based intervention in reducing binge drinking among nightclub patrons. Revista de saude publica. 2018;52:Article 2.

99. Charlebois E, Plenty A, Lin J, Ayala A, Hecht J. Impact of a structural intervention to address alcohol use among gay bar patrons in San Francisco: The PACE study. AIDS Behav. 2017;21:193-202.

100. Zawisza TT, Burgason KA. A donnybrook in downtown? Observations of controlling aggression and the use of effective place management in a southern entertainment area. Crime Prev Community Saf. 2020;22(1):1-16.
